# A Novel and Highly Regioselective Synthesis of New Carbamoylcarboxylic Acids from Dianhydrides

**DOI:** 10.1155/2014/725981

**Published:** 2014-01-06

**Authors:** Adrián Ochoa-Terán, Jesús Estrada-Manjarrez, Marisela Martínez-Quiroz, Marco A. Landey-Álvarez, Eleazar Alcántar Zavala, Georgina Pina-Luis, Hisila Santacruz Ortega, Luis Enrique Gómez-Pineda, José-Zeferino Ramírez, Daniel Chávez, Julio Montes Ávila, Victoria Labastida-Galván, Mario Ordoñez

**Affiliations:** ^1^Centro de Graduados e Investigación, Instituto Tecnológico de Tijuana, 22510 Tijuana, BC, Mexico; ^2^Departamento de Ingeniería Bioquímica, Instituto Tecnológico de Culiacán, 80220 Culiacán, SIN, Mexico; ^3^Facultad de Ciencias Químico Biológicas, Universidad Autónoma de Sinaloa, 80010 Culiacán, SIN, Mexico; ^4^Departamento de Polímeros y Materiales, Universidad de Sonora, 83000 Hermosillo, SON, Mexico; ^5^Centro de Investigaciones Químicas, Universidad Autónoma del Estado de Morelos, 62209 Cuernavaca, MOR, Mexico

## Abstract

A regioselective synthesis has been developed for the preparation of a series of *N*,*N*′-disubstituted 4,4′-carbonylbis(carbamoylbenzoic) acids and *N*,*N*′-disubstituted bis(carbamoyl) terephthalic acids by treatment of 3,3′,4,4′-benzophenonetetracarboxylic dianhydride (**1**) and 1,2,4,5-benzenetetracarboxylic dianhydride (**2**) with arylalkyl primary amines (**A-N**). The carbamoylcarboxylic acid derivatives were synthesized with good yield and high purity. The specific reaction conditions were established to obtain carbamoyl and carboxylic acid functionalities over the thermodynamically most favored imide group. Products derived from both anhydrides **1** and **2** were isolated as pure regioisomeric compounds under innovative experimental conditions. The chemo- and regioselectivity of products derived from dianhydrides were determined by NMR spectroscopy and confirmed by density functional theory (DFT). All products were characterized by NMR, FTIR, and MS.

## 1. Introduction

Some *N*-aryl 2-carbamoylbenzoic acids, commonly known as phthalamic acids, are auxin transport inhibitors in plants [1–4]. The activity of these phytotropins is directly related to the carboxylic acid group attached to an aromatic ring in the ortho position of a second aromatic ring through a conjugated or a planar system of atoms. The spatial requirement for a high activity is the specific distance between the two aromatic ring centers (7.3 Å). Other biological effects of this molecule in plants include the ability to inhibit the polar transport of auxins, to abolish the apical dominance effect, and to prevent the geo- and phototropic response. In particular the *N*-1-naphthyl-2-carbamoylbenzoic acid (NACBA) is known as a potent inhibitor of auxins transport and geotropic curvature. It also has the capacity to bind to fractions containing plasma membrane vesicles from maize coleoptiles.

Recently, the synthesis of *N*-aryl-2-carbamoylbenzoic acids under microwave assisted conditions from phthalic anhydride and aryl or heterocyclic amines in the absence of solvent with yields 51–99% has been reported [5]. Significant research findings show that these *N*-arylphthalamic acids induce hyperlipidemia in Swiss white mice and also an increase in body weight of the animals, in contrast with the hypolipidemic activity showed by their phthalimide analogues [6].

In addition, 2-carbamoylbenzoic acids and structurally related compounds have shown attractive pharmaceutical and agricultural applications as novel anti-inflammatory, immunomodulatory, antiproliferatory, antithrombotic, and antituberculosis agents, as well as plant growth regulators [7–10]. Recently, the synthesis of compounds containing a carbamoylbenzoic acid functionality, showing BMP 2 stimulation and osteoblast differentiation has been reported [11]. As well, these compounds have industrial applications as surfactants, emulsifiers, and conditioning agents in shampoos, and as part of antiperspirant formulations, among others [12, 13]. However, to the best of our knowledge, there are not reports in the literature regarding the synthesis, properties, or applications of carbamoylbenzoic acids bearing *N*-alkyl or *N*-alkylaryl substituents.

**Figure 1:**
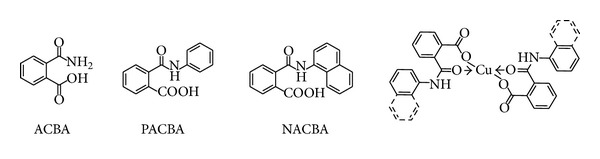
Carbamoybenzoic acids as monotopic ligands in organometallic complexes.

Lingaiah and coworkers have prepared organometallic complexes using 2-carbamoylbenzoic acid (ACBA), *N*-phenyl-2-carbamoylbenzoic acid (PACBA), and *N*-naphthyl-2-carbamoylbenzoic (NACBA) as monotopic ligands with Cu^2+^ (Figure 1) [14]. They found a stoichiometric 1 : 2 metal-ligand ratio in these complexes. In addition, they demonstrated that the interaction between ligands and metallic center is through an ionic metal-heteroatom bond with the carboxylate group and a coordinated bond with the amide carbonyl group. However, they have not been reported catalytic applications of these organometallic complexes.

**Scheme 1:**
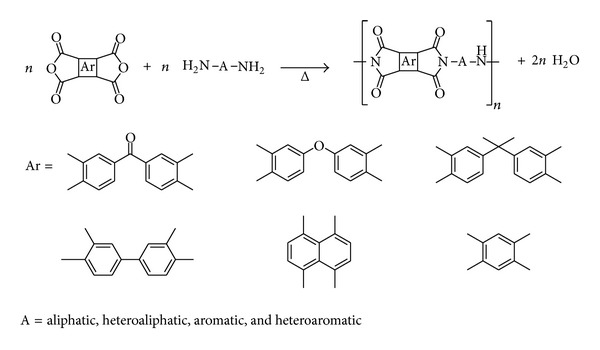
Synthesis of polyimides from aromatic dianhydrides.

Traditionally, aromatic dianhydrides are used as starting materials in the synthesis of water soluble polymers as polyimides (Scheme 1) [15–21]. These compounds are distinguished as high performance polymers due to their excellent chemical, mechanical, and dielectric resistance in a broad temperature interval. In the aerospace and electronic industry, polyimides are mainly used to form films and moldings. Other applications as adhesives, gas separation membranes, composite matrices, coatings, and foams are rapidly increasing [22–25].

**Scheme 2:**
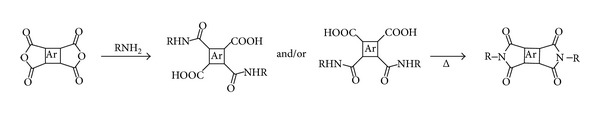
Synthesis of diimides from aromatic dianhydrides.

Aromatic dianhydrides, especially naphthalene and benzophenone dianhydrides, are commonly used in the synthesis of aromatic diimide monomers. These compounds have been extensively studied due to their interesting physicochemical properties and their applications [26–29]. However, the bis(carbamoylcarboxylic) acid intermediates have not received much attention due to the difficulty to obtain them in a chemoselective way and with high purity (Scheme 2) [30]. As above mentioned, these molecules are potentially bioactive compounds and ligands in organometallics and materials chemistry.

In this work, specific experimental conditions were implemented for the chemo- and regioselective synthesis and achievement of a series of aromatic bis(carbamoylcarboxylic) acids derived from benzophenone and benzene dianhydrides with good yields.

## 2. Results and Discussion

### 2.1. Synthesis and Characterization

**Figure 2:**
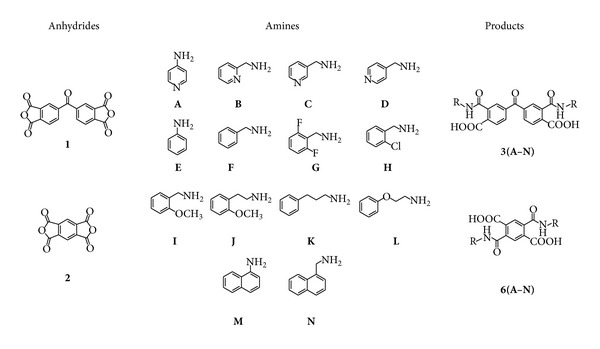
Substrates used for the synthesis of bis(carbamoylcarboxylic) acids.

The synthesis of bis(carbamoylcarboxylic) acids was developed using two different core dianhydride substrates (Figure 2), such as 3,3′,4,4′-benzophenonetetracarboxylic dianhydride (**1**) (formally two phthalic anhydrides linked by a carbonyl group) and 1,2,4,5-benzenetetracarboxylic dianhydride (**2**) (two anhydride groups with a benzene ring bridge). The selected primary amines contain phenyl, pyridyl, or naphthyl aromatics rings, attached directly to the amine group or separated by alkyl or heteroalkyl chains. Also, fluorine, chlorine, and methoxy substituents are present in some aromatic rings. The combination of different core structures and substituents leads to obtaining product diversity with a wide potential range of chemical, spectroscopic, and biological properties.

It is well known, that the synthesis of this type of compounds occurs by a nucleophilic attack of amine group to the anhydride ring to obtain the corresponding bis(carbamoylbenzoic) or bis(carbamoyl) terephthalic acid structure. Thus, reactions were performed in THF, Toluene, or EtOH as solvent depending of reactant solubility. Most of the products were isolated by filtration and suspended into a 5% HCl aqueous solution to eliminate the amine in excess.

The 4,4′-carbonyl bis(2-carbamoylbenzoic) acids **3**(**A-N**) were obtained in yields of 60 to >98% and high purity (Table 1). In their FTIR spectra, was observed the following: at 3390–3100 cm^−1^ the vibration corresponding to N–H and at 1670–1630 cm^−1^ the vibrations corresponding to the ketone, carboxylic acid, and amide carbonyl stretching. The signal corresponding to the amide hydrogen was not observable at low field in ^1^H NMR spectra, but a broad singlet at 7.60 to 4.00 ppm that disappeared when a D_2_O drop was added was assigned to this hydrogen. The HDO formed by the exchange of the NH hydrogen appeared at 4.05 to 3.65 ppm (see Supplementary Material available online at http://dx.doi.org/10.1155/2014/725981). There are signals belonging to the aromatic substituent on the amide group and three more at 8.54 to 8.06 ppm, 8.34 to 7.95 ppm, and 7.87 to 6.70 ppm for aromatic benzophenone core. In the ^13^C NMR spectra, the signals at 199.8 to 193.4 ppm, 168.3 to 167.1 ppm, and 168.1 to 167.0 ppm corresponding to ketone, carboxylic, and amide carbonyl groups are observable. The quasimolecular ions were not detected in ESIMS on neither negative nor positive modes. In EIMS adducts M^+^-2H_2_O that correspond to the diimide analogs formed by dehydration of two carbamoylcarboxylic acids units were detected.

The bis(carbamoyl) terephthalic acids **6**(**A-N**) were obtained in low to moderate yields (12 to 48%) when the reaction was carried out at room temperature in THF or EtOH. But when the mixture was refluxed in THF or Toluene the yields increase considerably (Table 1). All products were separated from the reaction mixture by filtration and washed with an aqueous HCl solution and obtained in high purity. When the filtrate was evaporated a solid product identified as the diimide analog was obtained in very low amounts.

Also, in their FTIR spectra was observed the following: at 3390–3100 cm^−1^ the vibration corresponding to N–H and at 1670–1630 cm^−1^ the vibrations corresponding to the carboxylic acid and amide carbonyl stretching. The ^1^H NMR spectra showed one signal for the amide group hydrogen as a triplet or singlet between 10.60 and 8.53 ppm. A singlet signal between 8.91 and 7.65 ppm that integrates for the two hydrogens at the aromatic core structure was found. In the ^13^C NMR spectra, two signals at 171.5 to 166.6 ppm and 168.1 to 165.8 ppm corresponding to carboxylic and amide carbonyl groups are observable. In EIMS adducts M^+^-2H_2_O that correspond to the diimide analogs formed by dehydration of two carbamoylcarboxylic acids units were detected.

### 2.2. Chemo- and Regioselectivity in the Ring-Opening of Dianhydrides

**Scheme 3:**
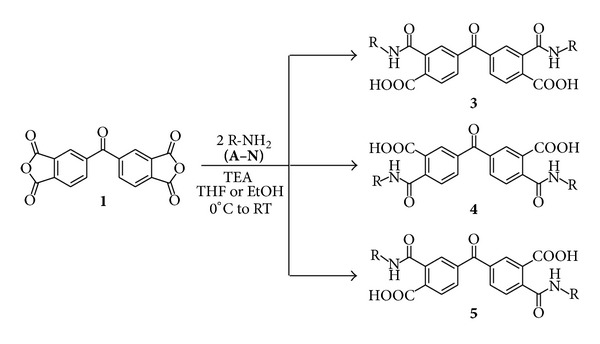
Plausible regioisomers obtained on the synthesis of 4,4′-carbonyl bis(2-carbamoylbenzoic) acids.

During the reaction of benzophenonetetracarboxylic dianhydride (**1**) with primary amines, it is possible to obtain three regioisomers (Scheme 3). Regioisomers **3**(**A-N**) and **4**(**A-N**) have symmetric structures and regioisomers **5**(**A-N**) are nonsymmetrical. The simplicity of the NMR signals showed that only one of the possible regioisomers was obtained. Based on the low signals number, the nonsymmetrical compounds **5**(**A-N**) were discarded.

**Figure 3:**
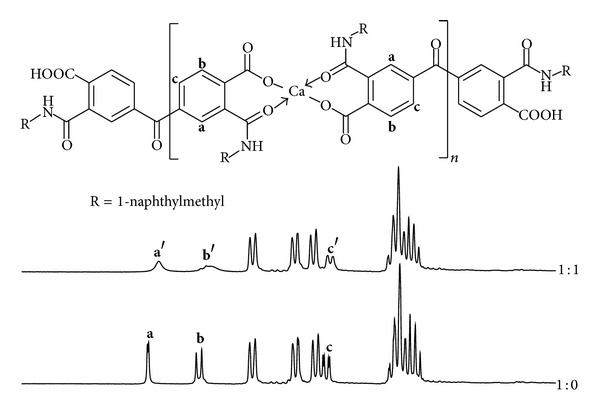
NMR titration of **3N** with Ca^2+^ ions in DMSO-*d*_6_.

To elucidate which regioisomer was favored under these conditions, an NMR titration of the 1-naphthylmethyl derivative (**3N**) was consummated using Ca^2+^ to form a complex in solution. As shown in Figure 3, the three signals of benzophenone core (**a**, **b**, and **c**) were affected in their chemical shift and shape when one equivalent of Ca^2+^ was added, indicating coordination between the molecule and the metal ion through the amide carbonyl and the ionic bond with the carboxylate group [14]. In the complex this part of the molecule becomes more rigid and relaxing times of **a**, **b**, and **c** hydrogens increase until the same level of acquisition time of the NMR instrument resulting in the broadening of the signals near to the coordination site [31–34]. Here the signal with higher broadening was the doublet at 8.30 ppm (signal **b**) which is expected to be more affected by the coordination in regioisomer **3** than in **4**, when carboxylate group coordinates with Ca^2+^. From this experimental result, regioisomers **4** were discarded.

**Scheme 4:**
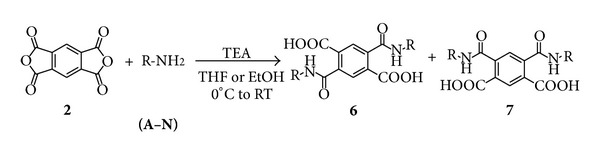
Plausible regioisomers obtained on the synthesis of bis(carbamoyl) terephthalic acids.

Meanwhile, as depicted in Scheme 4, 1,2,4,5-benzenetetracarboxylic dianhydride (**2**) was reacted with a series of primary amines and was expected to obtain a mixture of two regioisomeric products. If the amine attacks the carbonyl groups at opposite side of molecule, antisymmetric terephthalic acids **6**(**A-N**) could be obtained, while if the amine reacts with the carbonyl groups on the same side, symmetric terephthalic acids **7**(**A-N**) would be expected.

The ^1^H NMR spectra showed one signal for the amide group hydrogen as a triplet or singlet indicating the amide and carboxylic acid functionalities in these molecules. Only one singlet signal that integrates for the two hydrogens at the aromatic core structure was found indicating that the two hydrogens have identical chemical environment, which can be possible only in the antisymmetric regioisomers **6**(**A-N**) because both hydrogens are in ortho position to a carboxylic and amide group, while significant differences should be present in the symmetric regioisomer due to one hydrogen is in ortho position to two amide groups and the other one is in ortho position to two carboxylic acids. When reaction was performed in EtOH as solvent at room temperature, some compounds as **6F**/**7F** were isolated as a regioisomeric mixture. In the ^1^H NMR spectra of this sample two triplet appeared at 10.72 and 10.55 ppm assigned to amide hydrogen. But more importantly, a singlet signal at 7.88 ppm corresponding to the two aromatic core hydrogens of **6F** (antisymmetric regioisomer) and two singlet signals at 8.04 ppm and 7.73 ppm assigned to each aromatic core hydrogen of **7F** (symmetric regioisomer) were found. In products were the aromatic substituent is attached directly to the amide group, as compounds **6E**/**7E** and **6 M**/**7 M**, the NMR spectra shown both regioisomers perfectly identified because is present a singlet that corresponds to the two aromatic hydrogens of benzene core in products **6** and two singlet signals corresponding to each hydrogen of the benzene core in products **7**. Additionally, there are singlet signals at low field for the NH amide with different chemical shift (see Supplementary Material).

### 2.3. Computational Modeling in the Ring-Opening of Dianhydrides

**Figure 4:**
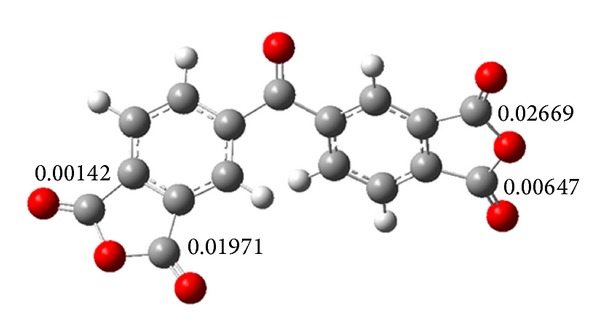
The Fukui function calculated for atoms involved in the ring-opening of dianhydride **1**.

As is well documented, chemical reactivity of a molecule can be described by Density Functional Theory (DFT) method, and the local quantity or Fukui function provides information about the reactive behavior of atoms forming a molecule. The Fukui function is defined as

(1) $$\begin{array}{c} f_{k}^{+}=q_{k}\left(N+1\right)-q_{k}\left(k\right), \end{array}$$

where *f*_*k*_^+^ represents the measure reactivity toward a nucleophilic reagent and *q*_*k*_ is the electronic population of atom *k* in the molecule [35]. The electronic population is evaluated in terms of the electrostatic potential (ESP) single point calculation [36]. A high value of *f*_*k*_^+^ indicates that atom *k* is highly reactive when compared to other atoms in the molecule. First, the geometry of dianhydride **1** was optimized and confirmed through the frequency calculation.  Figure 4 shows *f*_*k*_^+^ calculated for atoms involved in the ring opening. Based on Fukui values, the anhydride carbonyls attached at 3 and 3′ positions have higher probability for a nucleophilic attack. Therefore, we can assume that regioisomers **3**(**A-N**) are favored in the reaction with amines. This result is remarkably in agreement with the NMR titration.

**Figure 5:**
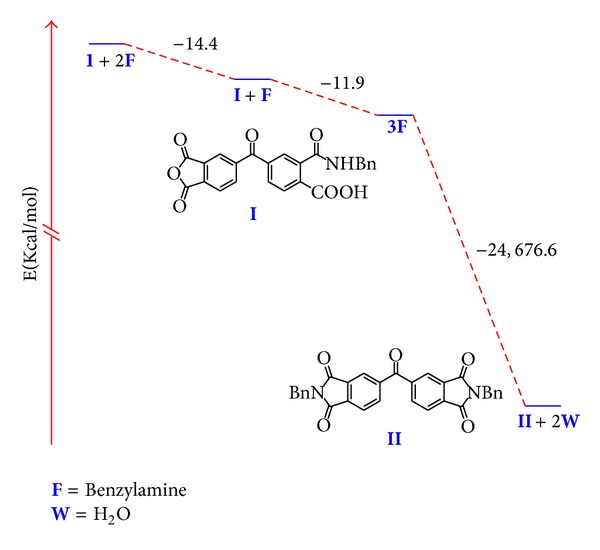
Energy diagram for the reaction pathway of dianhydride **1 **and benzylamine.

The energy diagram for the reaction pathway showed that benzophenone dianhydride has high energy, and is therefore a highly reactive compound (Figure 5). Conversion to the bis(carbamoylcarboxylic) functionality is an exothermic process. But it was more important to find that formation of diimide analog **II** is even more exothermic, which means that at experimental conditions used in the reaction, formation of diimide analogs are favorable too. However, the insolubility of products in the reaction media allowed isolating them in good yields.

**Figure 6:**
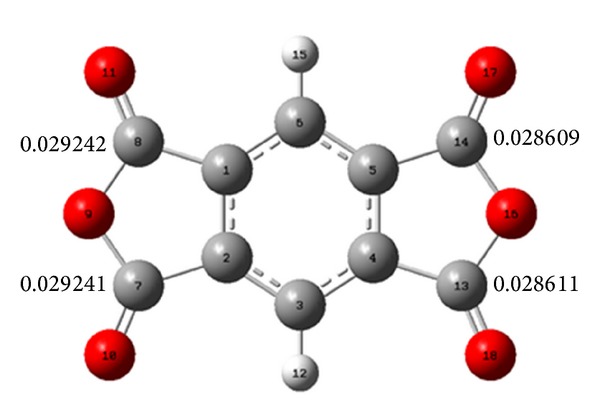
The Fukui function calculated for atoms involved in the ring-opening of dianhydride **2**.

As well as dianhydride **1**, Fukui values were calculated in dianhydride **2** finding that antisymmetric carbonyls are the most suitable sites for nucleophilic attack (Figure 6).

**Figure 7:**
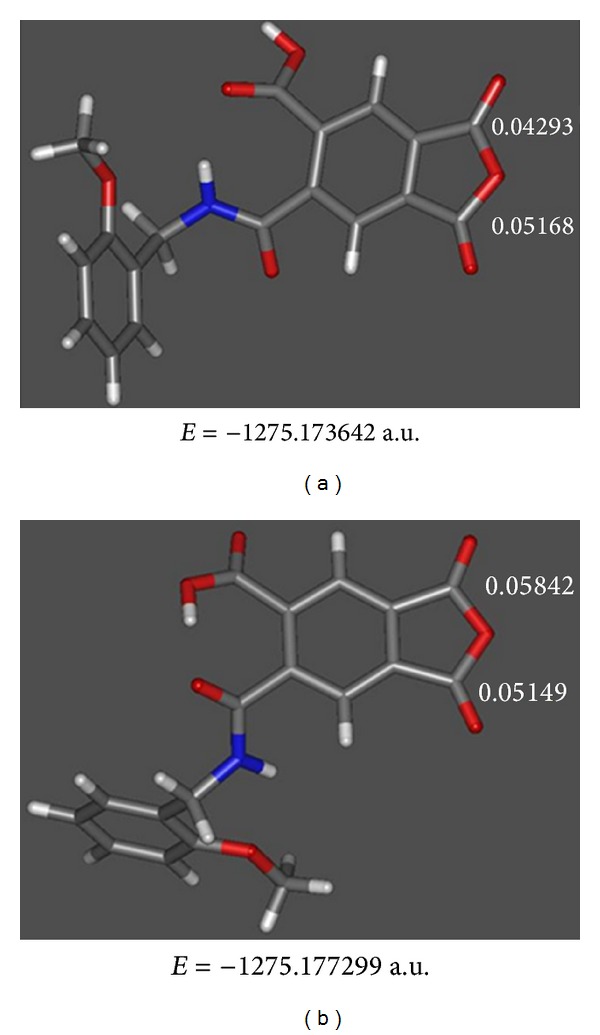
Optimized geometries of a monocarbamoylcarboxylic acid intermediate indicating Fukui function values. (a) No intramolecular hydrogen bond. (b) Intramolecular hydrogen bond.

Moreover, Fukui functions of monocarbamoylcarboxylic acid derived from 2-methoxybenzylamine was calculated. In Figure 7 the structures of the two most stable geometries for this intermediate are shown. In the first one, Fukui function defines the same side carbonyl as the most suitable site for the nucleophilic attack; but in the second, where an intramolecular hydrogen bond stabilizes the molecule in a 2.3 kcal/mol, Fukui function defines the opposite side carbonyl as the most favored for the reaction with the amine. Intramolecular hydrogen bond between carboxylic hydrogen and amide carbonyl determinate the geometry as well as the reactivity of this intermediate.

This result is also in agreement with regioselectivity observed in different solvents. Nonprotic solvent as THF or Toluene favors the intramolecular hydrogen bond and as consequence the antisymmetric regioisomer formation. A protic solvent as EtOH competes in the hydrogen bond formation modifying the molecule geometry and reactivity.

**Figure 8:**
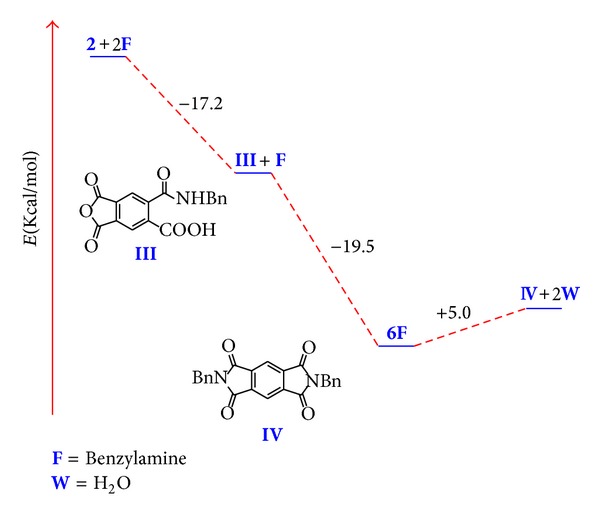
Energy diagram for the reaction pathway of dianhydride **2 **and benzylamine.

The energy diagram for the reaction pathway is in agreement with the experimental conditions that favor the observed chemoselectivity and high yields. The synthesis of monocarbamoylcarboxylic intermediate **III** is an exothermic process as well as the opening of the second anhydride ring. On the contrary diimide analog **IV **formation is an endothermic process; therefore the heating in the reaction favors diimide formation only in small amounts (Figure 8). Nevertheless, bis(carbamoyl) terephthalic acids are obtained in good yields due to their insolubility in the reaction media.

## 3. Conclusions

In this work 28 bis(carbamoylcarboxylic) type compounds derived from two different dianhydrides and fourteen primary amines were successfully synthesized and characterized. Controlled reaction conditions were established to obtain these compounds over diimide analogs with moderate to quantitative yields. The chemo- and regioselectivity found in products derived from dianhydrides were determined by NMR spectroscopy and supported by theoretical calculations at DFT level. We believe these products have potential uses as bioactive molecules, and as components in industrial formulations or using monotopic and ditopic ligands in the synthesis of organometallic complexes. Currently, the research group is exploring the above mentioned capabilities of the new synthesized compounds.

## 4. Experimental Section

### 4.1. General Information

All reagents were purchased in the highest quality available and were used without further purification. Infrared spectra (FTIR) were recorded on a spectrophotometer. Nuclear magnetic resonance ^1^H (at 200 MHz) and ^13^C (at 50 MHz) spectra were recorded on a 200 MHz spectrometer in DMSO-*d*_6_ or D_2_O with TMS as internal standard. Electrospray ionization mass spectra (ESIMS) were obtained with an ion trap, and the intensities are reported as a percentage relative to the base peak after the corresponding *m*/*z* value. Electron impact mass spectra (EIMS) with direct insertion were performed on mass spectrometer. High resolution mass spectra (HRMS) were obtained with FAB (gly) or chemical ionization (CH_4_) on positive or negative modes.

### 4.2. General Procedure for the Synthesis of 4,4′-Carbonylbis(carbamoylbenzoic) Acids

To a solution 1,2,4,5-benzenetetracarboxylic dianhydride (**1**) (100 mg, 0.31 mmol) in 30 mL of THF or EtOH at 0°C was added the corresponding primary amine (0.68 mmol, 2.2 equiv.) and the reaction mixture was stirred at RT for 3 h. The solid product was separated by filtration and then put into a HCl 5% aqueous solution in order to eliminate the unreactive amine. The solid was recovered by filtration and washed tin ethyl acetate and methylene chloride. 

*4,4*′*-Carbonyl Bis(2-(pyridin-4-ylcarbamoyl)benzoic Acid)* ***(3A)***. White solid. 148 mg, 0.29 mmol, 92%. Mp 340–342°C FTIR: 3220, 3098, 2927, 1663, 1650, 1599, 1455, 1366 cm^−1^. MS (EI) *m*/*z*: 474 (M^+^-2H_2_O, 2), 398 (90), 354 (23), 322 (10), 278 (87), 103 (100), 75 (95). ^1^H NMR (DMSO-*d*_6_, 200 MHz): *δ* 8.52 (d, *J* = 2.0 Hz, 2H), 8.33 (d, *J* = 8.2 Hz, 2H), 8.09 (d, *J* = 7.0 Hz, 4H), 7.93 (brs, 2H), 7.87 (dd, *J* = 8.2, 2.0 Hz, 2H), 6.77 (d, *J* = 7.0 Hz, 4H), 4.60 (brs, 2H). ^13^C NMR (DMSO-*d*_6_, 50 MHz): *δ* 194.7, 167.3, 167.2, 159.7, 140.3, 138.5, 137.9, 135.0, 133.9, 133.1, 131.0, 108.9. HRMS (CI−) Calculated for C_27_H_14_N_4_O_5_ (M-2H_2_O)^−^ 474.0964; Found 474.0961. 

*4,4*′*-Carbonyl Bis(2-(pyridin-2-ylmethylcarbamoyl)benzoic Acid)* ***(3B)***. White solid. 102 mg, 0.19 mmol, 60%. Mp 138–140°C. FTIR: 3321, 2048, 2860, 1656, 1641, 1595, 1543, 1354 cm^−1^. MS (EI) *m*/*z*: 502 (M^+^-2H_2_O, 2), 412 (55), 367 (22), 321 (25), 278 (100), 174 (50). ^1^H NMR (DMSO-*d*_6_, 200 MHz): *δ* 8.56 (s, 2H), 8.54 (d, *J* = 2.0 Hz, 2H), 8.34 (d, *J* = 8.2 Hz, 2H), 7.87 (dd, *J* = 8.2, 2.0 Hz, 2H), 7.81 (dd, *J* = 7.6, 1.8 Hz, 4H), 7.45 (d, *J* = 7.8 Hz, 2H), 7.31 (dd, *J* = 6.2, 5.0 Hz, 2H), 4.01 (s, 4H). ^13^C NMR (DMSO-*d*_6_, 50 MHz): *δ* 194.7, 167.2, 167.1, 157.5, 148.8, 138.5, 137.8, 136.9, 135.0, 133.9, 133.0, 130.9, 122.6, 121.8, 44.8. HRMS (CI−) Calculated for C_29_H_18_N_4_O_5_ (M-2H_2_O)^−^ 502.1277; Found 502.1280. 

*4,4*′*-Carbonyl Bis(2-(pyridin-3-ylmethylcarbamoyl)benzoic Acid)* ***(3C)***. White solid. 108 mg, 0.20 mmol, 63%. Mp 116–118°C. FTIR: 3387, 3043, 2967, 2868, 1649, 1635, 1602, 1538, 1388, 1358 cm^−1^. MS (EI) *m*/*z*: 502 (M^+^-2H_2_O, 3), 411 (10), 320 (15), 174 (100). ^1^H NMR (DMSO-*d*_6_, 200 MHz): *δ* 8.55 (s, 2H), 8.52 (d, *J* = 1.8 Hz, 2H), 8.33 (d, *J* = 8.0 Hz, 2H), 7.86 (dd, *J* = 8.0, 1.8 Hz, 4H), 7.80 (td, *J* = 7.6, 1.8 Hz, 2H), 7.45 (d, *J* = 7.6 Hz, 4H), 7.30 (t, *J* = 7.6, 2H), 5.18 (brs, 2H), 4.01 (s, 4H). ^13^C NMR (DMSO-*d*_6_, 50 MHz): *δ* 194.7, 167.2, 167.1, 157.8, 148.7, 138.5, 137.8, 136.8, 135.0, 133.9, 133.0, 130.8, 122.4, 121.7, 44.9. HRMS (CI−) Calculated for C_29_H_18_N_4_O_5_ (M-2H_2_O)^−^ 502.1277; Found 502.1280. 

*4,4*′*-Carbonyl Bis(2-(pyridin-4-ylmethylcarbamoyl)benzoic Acid)* ***(3D)***. White solid. 167 mg, 0.31 mmol, >98%. Mp 178–180°C. FTIR: 3134, 3032, 2906, 2811, 1660, 1633, 1604, 1517, 1382, 1364 cm^−1^. MS (EI) *m*/*z*: 502 (M^+^-2H_2_O, 3), 412 (10), 321 (15), 278 (100), 174 (60). ^1^H NMR (DMSO-*d*_6_, 200 MHz): *δ* 8.54 (d, *J* = 6.0 Hz, 4H), 8.53 (d, *J* = 2.0 Hz, 1H), 8.33 (d, *J* = 8.2 Hz, 2H), 7.87 (dd, *J* = 8.2, 2.0 Hz, 2H), 7.40 (d, *J* = 6.0 Hz, 4H), 5.62 (brs, 2H), 3.93 (s, 4H). ^13^C NMR (DMSO-*d*_6_, 50 MHz): *δ* 194.6, 167.3, 167.2, 149.5, 147.7, 138.5, 137.8, 135.0, 133.9, 133.0, 130.8, 122.6, 42.7. HRMS (CI−) Calculated for C_29_H_18_N_4_O_5_ (M-2H_2_O)^−^ 502.1277; Found 502.1252. 

*4,4*′*-Carbonyl Bis(2-(phenylcarbamoyl)benzoic Acid)* ***(3E)***. White solid. 132 mg, 0.26 mmol, 83%. FTIR: 3354, 3036, 1705, 1657, 1599, 1495, 1230 cm^−1^. Mp 318–320°C. MS (EI) *m*/*z*: 472 (M^+^-2H_2_O, 3), 397 (40), 322 (30), 278 (100), 175 (25), 103 (60). ^1^H NMR (DMSO-*d*_6_, 200 MHz): *δ* 8.09 (d, *J* = 1.4 Hz, 2H), 7.95 (dd, *J* = 8.0, 1.4 Hz, 2H), 7.89 (t, *J* = 7.4 Hz, 4H), 7.60 (brs, 2H), 7.05 (dd, *J* = 8.4, 7.4 Hz, 4H), 6.63 (d, *J* = 7.8 Hz, 4H), 6.57 (t, *J* = 8.4 Hz, 2H). ^13^C NMR (DMSO-*d*_6_, 50 MHz): *δ* 193.5, 168.2, 167.4, 146.9, 137.8, 137.4, 132.5, 132.1, 130.1, 129.1 128.9, 117.0, 114.8. HRMS (CI−) Calculated for C_29_H_16_N_2_O_5_ (M-2H_2_O)^−^ 472.1059; Found 472.1048. 

*4,4*′*-Carbonyl Bis(2-(benzylcarbamoyl)benzoic Acid)* ***(3F)***. White solid. 140 mg, 0.26 mmol, 85%. Mp 156–158°C. MS (EI) *m*/*z*: 500 (M^+^-2H_2_O, 100), 423 (20), 409 (25), 208 (18), 91 (95). FTIR (KBr): 3032, 2938, 1655, 1243, 1084 cm^−1^. ^1^H NMR (DMSO-*d*_6_, 200 MHz): *δ* 8.52 (d, *J* = 2.0 Hz, 2H), 8.33 (d, *J* = 8.0 Hz, 2H), 7.86 (dd, *J* = 8.0, 2.0 Hz, 2H), 7.36 (m, 10H), 5.48 (brs, 2H), 3.95 (s, 4H). ^13^C NMR (DMSO-*d*_6_, 50 MHz): *δ* 194.7, 167.3, 167.2, 138.6, 137.8, 133.9, 132.9, 130.8, 128.5, 128.3, 127.8, 43.3. HRMS (CI−) Calculated for C_31_H_20_N_2_O_5_ (M-2H_2_O)^−^ 500.1372; Found 500.1378. 

*4,4*′*-Carbonyl Bis(2-(2,6-difluorobenzylcarbamoyl)benzoic Acid)* ***(3G)***. White solid. 189 mg, 0.31 mmol, >98%. Mp 146–148°C. FTIR: 3389, 3058, 1660, 1630, 1537, 1468, 1239 cm^−1^. MS (EI) *m*/*z*: 572 (M^+^-2H_2_O, 20), 447 (10), 320 (45), 375 (40), 127 (100). ^1^H NMR (DMSO-*d*_6_, 200 MHz): *δ* 8.52 (d, *J* = 2.0 Hz, 2H), 8.33 (d, *J* = 8.2 Hz, 2H), 7.86 (dd, *J* = 8.2, 2.0 Hz, 2H), 7.44 (qnt, *J* = 8.0 Hz, 4H), 7.12 (t, *J* = 8.0, 2H), 4.60 (brs, 2H), 3.90 (s, 4H). ^13^C NMR (DMSO-*d*_6_, 50 MHz): *δ* 194.6, 167.1, 167.0, 160.8 (dd, *J* = 246.1, 8.4 Hz), 138.4, 137.7, 134.9, 133.9, 133.0, 130.8, 130.4 (t, *J* = 10.4 Hz) 111.6 (d, *J* = 23.9 Hz), 111.5 (d, *J* = 22.5 Hz), 31.7 (t, *J* = 4 Hz). HRMS (CI−) Calculated for C_31_H_16_F_4_N_2_O_5_ (M-2H_2_O)^−^ 572.0995; Found 572.1003. 

*4,4*′*-Carbonyl Bis(2-(2-chlorobenzylcarbamoyl)benzoic Acid)* ***(3H)***. White solid. 188 mg, 0.31 mmol, >98%. Mp 122–124°C. FTIR: 3235, 3010, 2967, 1700, 1631, 1516, 1274 cm^−1^. MS (EI) *m*/*z*: 533 (M^+^-Cl-2H_2_O, 100), 427 (10), 249 (25), 125 (30). ^1^H NMR (DMSO-*d*_6_, 200 MHz): *δ* 8.52 (d, *J* = 2.0 Hz, 2H), 8.33 (d, *J* = 8.0 Hz, 2H), 7.86 (dd, *J* = 8.0, 2.0 Hz, 2H), 7.56 (dd, *J* = 7.2, 2.6 Hz, 2H), 7.47 (dd, *J* = 7.0, 2.4 Hz, 2H), 7.37 (m, 4H), 4.37 (brs, 2H), 3.97 (s, 4H). ^13^C NMR (DMSO-*d*_6_, 50 MHz): *δ* 194.6, 167.1, 167.0, 138.5, 137.8, 134.9, 133.9, 133.0, 132.4, 130.8, 129.7, 129.3, 129.2, 129.1, 127.3, 41.3. HRMS (CI−) Calculated for C_31_H_18_Cl_2_N_2_O_5_ (M-2H_2_O)^−^ 568.0593; Found 568.0622. 

*4,4*′*-Carbonyl Bis(2-(2-methoxybenzylcarbamoyl)benzoic Acid)* ***(3I)***. White solid. 185 mg, 0.31 mmol, >98%. Mp 186–188°C. FTIR: 3254, 2997, 1656, 1629, 1522, 1257, 1268 cm^−1^. MS (EI) *m*/*z*: 560 (M^+^-2H_2_O, 45), 439 (30), 333 (18), 121 (100). ^1^H NMR (DMSO-*d*_6_, 200 MHz): *δ* 8.52 (d, *J* = 2.0 Hz, 2H), 8.33 (d, *J* = 8.2, 2H), 7.85 (dd, *J* = 8.2, 2.0 Hz, 2H), 7.33 (d, *J* = 8.2 Hz, 2H), 7.28 (dd, *J* = 7.4, 1.7 Hz, 2H), 7.00 (d, *J* = 8.2 Hz, 2H), 6.94 (t, *J* = 7.4 Hz, 2H), 4.00 (brs, 2H), 3.83 (s, 2H), 3.80 (s, 4H). ^13^C NMR (DMSO-*d*_6_, 50 MHz): *δ* 194.7, 167.1, 167.0, 156.9, 138.5, 137.8, 135.0, 133.9, 133.0, 130.8, 129.1, 129.0, 126.2, 120.2, 110.5, 55.3, 39.1. HRMS (CI−) Calculated for C_33_H_24_N_2_O_7_ (M-2H_2_O)^−^ 560.1584; Found 560.1609. 

*4,4*′*-Carbonyl Bis(2-(2-methoxyphenethylcarbamoyl)benzoic Acid)* ***(3J)***. White solid. 186 mg, 0.30 mmol, 96%. Mp 112–114°C. FTIR: 3335, 2981, 1706, 1635, 1575, 1356 cm^−1^. MS (EI) *m*/*z*: 588 (M^+^-2H_2_O, 7), 455 (14), 278 (25), 134 (100). ^1^H NMR (DMSO-*d*_6_, 200 MHz): *δ* 8.47 (d, *J* = 1.8 Hz, 2H), 8.27 (d, *J* = 8.0 Hz, 2H), 7.83 (dd, *J* = 8.0, 1.8 Hz, 2H), 7.20 (dd, *J* = 8.0, 1.6 Hz, 2H), 7.13 (dd,*J* = 7.4, 1.6 Hz, 2H), 6.95 (d, *J* = 8.0 Hz, 2H), 6.86 (t, *J* = 7.4 Hz, 2H), 4.00 (brs, 2H), 3.76 (s, 6H), 2.78 (m, 8H). ^13^C NMR (DMSO-*d*_6_, 50 MHz): *δ* 194.8, 167.7, 167.5, 157.2, 137.7, 135.3, 133.7, 132.8, 132.7, 130.8, 130.1, 127.9, 126.3, 120.4, 110.8, 55.3, 48.7, 30.8. HRMS (CI−) Calculated for C_35_H_28_N_2_O_7_ (M-2H_2_O)^−^ 588.1897; Found 588.1866. 

*4,4*′*-Carbonyl Bis(2-(3-phenylpropylcarbamoyl)benzoic Acid)* ***(3K)***. Yellow oil. 116 mg, 0.20 mmol, 63%. FTIR: 3381, 3024, 1699, 1649, 1549, 1356, 1238 cm^−1^. MS (EI) *m*/*z*: 556 (M^+^-2H_2_O, 5), 439 (50), 335 (100), 278 (90). ^1^H NMR (DMSO-*d*_6_, 200 MHz): *δ* 8.42 (d, *J* = 1.8 Hz, 2H), 8.19 (d, *J* = 8.0 Hz, 2H), 7.80 (d, *J* = 8.0, 1.8 Hz, 2H), 7.30–7.15 (m, 10H), 4.85 (brs, 2H), 2.73 (t, *J* = 7.4 Hz, 4H), 2.59 (t, *J* = 7.4 Hz, 4H), 1.78 (qnt, *J* = 7.4 Hz, 4H). ^13^C NMR (DMSO-*d*_6_, 50 MHz): *δ* 194.8, 168.3, 168.1, 141.2, 139.6, 139.5, 137.5, 135.7, 133.2, 132.0, 130.5, 128.4, 128.2, 121.1, 125.9, 39.0, 32.0, 30.1. HRMS (CI−) Calculated for C_35_H_28_N_2_O_5_ (M-2H_2_O)^−^ 556.1998; Found 556.2010. 

*4,4*′*-Carbonyl Bis(2-(2-phenoxyethylcarbamoyl)benzoic Acid)* ***(3L)***. Yellow oil. 137 mg, 0.23 mmol, 74%. FTIR: 3378, 3040, 1714, 1641, 1491, 1361, 1238 cm^−1^. MS (EI) *m*/*z*: 560 (M^+^-2H_2_O, 6), 467 (35), 348 (100), 120 (30). ^1^H NMR (DMSO-*d*_6_, 200 MHz): *δ* 8.50 (d, *J* = 2.0 Hz, 2H), 8.31 (d, *J* = 8.0 Hz, 2H), 7.85 (dd, *J* = 8.0, 2.0 Hz, 2H), 7.29 (dd, *J* = 7.6, 7.2 Hz, 4H), 7.00-6.88 (m, 6H), 4.02 (t, *J* = 5.4 Hz, 4H), 3.98 (brs, 2H), 3.04 (brt, 4H). ^13^C NMR (DMSO-*d*_6_, 50 MHz): *δ* 194.8, 167.4, 167.3, 158.3, 138.7, 137.9, 135.1, 133.9, 133.0, 130.9, 129.6, 120.9, 114.6, 67.2, 39.8. HRMS (CI−) Calculated for C_33_H_24_N_2_O_7_ (M-2H_2_O)^−^ 560.1584; Found 560.1534. 

*4,4*′*-Carbonyl Bis(2-(naphthalen-1-ylcarbamoyl)benzoic Acid)* ***(3M)***. White solid. 189 mg, 0.31 mmol, >98%. Mp 198–200°C. FTIR: 3374, 2970, 1699, 1657, 1510, 1364, 1238 cm^−1^. MS (EI) *m*/*z*: 572 (M^+^-2H_2_O, 5), 447 (100), 278 (20), 228 (25), 127 (40). ^1^H NMR (DMSO-*d*_6_, 200 MHz): *δ* 8.06 (d, *J* = 1.6 Hz, 2H), 8.05-8.03 (m, 2H), 7.95 (dd, *J* = 8.0, 1.6 Hz, 2H), 7.84 (d, *J* = 7.8 Hz, 2H), 7.73 (dd, *J* = 7.0, 2.6 Hz, 2H), 7.75–7.46 (m, 4H), 7.21 (dd, *J* = 8.0, 7.4 Hz, 2H), 7.10 (d, *J* = 7.8, 2H), 6.70 (dd, *J* = 7.4, 1.4 Hz, 2H), 4.22 (brs, 2H). ^13^C NMR (DMSO-*d*_6_, 50 MHz): *δ* 193.4, 168.3, 167.4, 144.0, 137.8, 137.3, 134.1, 132.3, 132.2, 129.8, 128.8, 127.8, 126.7, 125.5, 123.8, 122.9, 122.3, 116.0, 108.0. HRMS (CI−) Calculated for C_37_H_20_N_2_O_5_ (M-2H_2_O)^−^ 572.1372; Found 572.1355. 

*4,4*′*-Carbonyl Bis(2-(naphthalen-1-ylmethylcarbamoyl)benzoic Acid)* ***(3N)***. White solid. 193 mg, 0.30 mmol, 98%. Mp 200–202°C. MS (EI) *m*/*z*: 600 (M^+^-2H_2_O, 70), 472 (30), 141 (100). FTIR: 3124, 3048, 2926, 1657, 1625, 1602, 1518 cm^−1^. ^1^H NMR (DMSO-*d*_6_, 200 MHz): *δ* 8.52 (d, *J* = 2.0 Hz; 2H), 8.32 (d, *J* = 8.2 Hz, 2H), 8.13 (d, *J* = 8.2, 2H), 7.97 (d, *J* = 8.0, Hz, 4H), 7.89 (d, *J* = 8.0 Hz, 2H), 7.86 (dd, *J* = 8.2, 2.0 Hz, 2H), 7.62–7.50 (m, 8H), 4.37 (s, 4H). ^13^C NMR (DMSO-*d*_6_, 50 MHz): *δ* 194.7, 167.1, 167.0, 138.5, 137.8, 135.0, 134.4, 133.9, 133.2, 133.0, 130.8, 128.5, 127.9, 126.3, 125.9, 125.8, 125.4, 123.4, 41.1. HRMS (CI−) Calculated for C_39_H_24_N_2_O_5_ (M-2H_2_O)^−^ 600.1685; Found 600.1639. 

### 4.3. General Procedure for the Synthesis of Bis(carbamoyl) Terephthalic Acids

*Method 1.* To a solution 1,2,4,5-benzenetetracarboxylic dianhydride (**2**) (100 mg, 0.46 mmol) in 30 mL of THF or EtOH at 0°C the corresponding primary amine was added (1.01 mmol, 2.2 equiv.) and the reaction mixture was stirred at RT for 3 h. The solid product was separated by filtration and then put into an HCl 5% aqueous solution in order to eliminate the unreactive amine. The solid was recovered by filtration and washed with ethyl acetate and methylene chloride. 

*Method 2.* To a solution 1,2,4,5-benzenetetracarboxylic dianhydride (**2**) (100 mg, 0.46 mmol) in 30 mL of THF or Toluene was added the corresponding primary amine (1.01 mmol, 2.2 equiv.) and the reaction mixture was refluxed for 4 h. The solid product was separated by filtration and then put into an HCl 5% aqueous solution in order to eliminate the unreactive amine. The solid was recovered by filtration and washed with ethyl acetate and methylene chloride. 

*2,5-Bis(pyridin-4-ylcarbamoyl)terephthalic Acid* ***(6A)***. White solid. 187 mg, 0.46 mmol, >98%. Mp 290–292°C. FTIR: 3302, 3097, 1701, 1640, 1565, 1490 cm^−1^. EIMS *m*/*z*: 370 (M^+^-2H_2_O, 11), 174 (100), 102 (50), 74 (40). MS (ESI−) *m*/*z*: 405 (M−H)^−^ MS/MS: 405 (2), 361 (5), 317 (100). ^1^H NMR (DMSO-*d*_6_, 200 MHz): *δ* 8.95 (s, 2H), 8.10 (d, *J* = 7.2 Hz, 4H), 7.86 (s, 2H), 6.76 (d, *J* = 7.2 Hz, 4H), 4.41 (brs, 2H). ^13^C NMR (DMSO-*d*_6_, 50 MHz): *δ* 168.3, 168.1, 158.1, 143.0, 134.1, 129.0, 127.6. HRMS (CI+) Calculated for C_20_H_11_N_4_O_4_ (M-2H_2_O+1) 371.0780; Found 371.0801. 

*2,5-Bis(pyridin-2-ylmethylcarbamoyl)terephthalic Acid* ***(6B)***. White solid. 136 mg, 0.31 mmol, 68%. Mp 218–220°C. FTIR: 3242, 3065, 2919, 1702, 1641, 1633, 1550, 1291, 1254 cm^−1^. MS (EI) *m*/*z*: 398 (M^+^-2H_2_O, 100), 353 (70), 308 (50), 263 (30). ^1^H NMR (D_2_O-DCl, 200 MHz): *δ* 8.74 (d, *J* = 6.6 Hz, 2H), 8.61 (t, *J* = 8.8 Hz, 2H), 8.15 (s, 2H), 8.12 (d, *J* = 8.8 Hz, 2H), 8.00 (t, *J* = 6.6 Hz, 2H), 4.96 (s, 4H). ^13^C NMR (D_2_O-DCl, 50 MHz): *δ* 171.5, 168.0, 152.1, 147.6, 141.1, 137.7, 132.7, 129.8, 126.3, 126.2, 41.2. HRMS (CI+) Calculated for C_22_H_15_N_4_O_4_ (M-2H_2_O+1) 399.1093; Found 399.1082. 

*2,5-Bis(pyridin-3-ylmethylcarbamoyl)terephthalic Acid* ***(6C)***. White solid. 178 mg, 0.42 mmol, 89%. Mp 264–266°C. FTIR: 3250, 3001, 2911, 1654, 1628, 1521, 1405, 1336 cm^−1^. MS (EI) *m*/*z*: 398 (M^+^-2H_2_O, 100), 238 (40), 208 (10). MS (ESI+) *m*/*z*: 435 (M+H)^+^. MS (ESI−) *m*/*z*: 433 (M−H)^−^ MS/MS 433(2), 390(15), 346(100). ^1^H NMR (D_2_O-DCl, 200 MHz): *δ* 8.82 (s, 2H), 8.72 (d, *J* = 6.0 Hz, 2H), 8.63 (dt, *J* = 8.2, 1.8 Hz, 2H), 8.08 (s, 2H), 8.06 (dd, *J* = 8.2, 6.0 Hz, 2H), 4.77 (s, 4H). ^13^C NMR (D_2_O-DCl, 50 MHz): *δ* 171.2, 167.9, 146.2, 140.2, 138.5, 137.9, 134.3, 132.5, 129.8, 127.5, 40.6. HRMS (CI+) Calculated for C_22_H_15_N_4_O_4_ (M-2H_2_O+1) 399.1093; Found 399.1093.

*2,5-Bis(pyridin-4-ylmethylcarbamoyl)terephthalic Acid* ***(6D)***. White solid. 164 mg, 0.38 mmol, 82%. Mp 296–298°C. FTIR: 3329, 3064, 1704, 1642, 1605, 1535, 1267 cm^−1^. EIMS *m*/*z*: 398 (M^+^-2H_2_O, 100), 238 (20), 208 (10). MS (ESI+) *m*/*z*: 435 (M+H)^+^. MS (ESI−) *m*/*z*: 433 (M−H)^−^. ^1^H NMR (D_2_O-DCl, 200 MHz): *δ* 8.75 (d, *J* = 6.8 Hz, 4H), 8.16 (s, 2H), 8.09 (d, *J* = 6.8, 4H), 4.89 (s, 4H). ^13^C NMR (D_2_O-DCl, 50 MHz): *δ* 171.3, 168.0, 159.7, 141.0, 137.9, 132.8, 129.8, 125.4, 43.1. HRMS (CI+) Calculated for C_22_H_11_N_4_O_4_ (M-2H_2_O+1) 399.1093; Found 399.1106.

*2,5-Bis(phenylcarbamoyl)terephthalic Acid* ***(6E)***. White solid. 118 mg, 0.29 mmol, 63%. Mp 346–348°C. FTIR: 3295, 3024, 1694, 1648, 1536, 1264 cm^−1^. MS (EI) *m*/*z*: 368 (M^+^-2H_2_O, 6), 293 (10), 174 (80), 93 (100). MS (ESI+) *m*/*z*: 427 (M+Na)^+^. MS (ESI−) *m*/*z*: 431 (M−H)^−^ MS/MS 431(2), 388(15), 344(100). ^1^H NMR (DMSO-*d*_6_, 200 MHz): *δ* 10.53 (s, 2H), 7.96 (s, 2H), 7.70 (d, *J* = 7.4 Hz, 4H), 7.36 (t, *J* = 7.4 Hz, 4H) 7.11 (t, *J* = 7.4 Hz, 2H). ^13^C NMR (DMSO-*d*_6_, 50 MHz): *δ* 166.6, 165.8, 141.2, 139.2, 139.1, 132.9, 128.7, 123.6, 119.6. HRMS (CI-) Calculated for C_22_H_12_N_4_O_4_ (M-2H_2_O)^−^ 368.0797; Found 368.0785. 

*2,5-Bis(benzylcarbamoyl)terephthalic Acid* ***(6F)***. White solid. 179 mg, 0.41 mmol, 90%. Mp 280–282°C. FTIR: 3272, 3033, 2905, 1694, 1643, 1544, 1301, 1266 cm^−1^. MS (EI) *m*/*z*: 396 (M^+^-2H_2_O, 10), 307 (20), 174 (100), 106 (100). MS (ESI+) *m*/*z*: 455 (M+Na)^+^. ^1^H NMR (DMSO-*d*_6_, 200 MHz): *δ* 13.46 (brs, 2H), 9.07 (t, *J* = 6.0 Hz, 2H), 7.79 (s, 2H), 7.33 (m, 10H), 4.46 (d, *J* = 6.0 Hz, 4H). ^13^C NMR (DMSO-*d*_6_, 50 MHz): *δ* 167.1, 167.0, 139.2, 138.5, 133.3, 128.2, 127.3, 126.7, 42.6. HRMS (CI+) Calculated for C_24_H_17_N_2_O_4_ (M-2H_2_O+1) 397.1188; Found 397.1214. 

*2,5-Bis(2,6-difluorobenzylcarbamoyl)terephthalic Acid* ***(6G)***. White solid. 186 mg, 0.37 mmol, 80%. Mp 260–262°C. FTIR: 3205, 3035, 1643, 1626, 1589, 1556, 1470, 1328 cm^−1^. MS (EI) *m*/*z*: 468 (M^+^-2H_2_O, 7), 343 (25), 174 (100), 142 (90), 102 (40). MS (ESI+) *m*/*z*: 526 (M+Na)^+^, 505 (M+H)^+^. MS (ESI−) *m*/*z*: 503 (M−H)^−^. ^1^H NMR (DMSO-*d*_6_, 200 MHz): *δ* 8.95 (t, *J* = 5.2 Hz, 2H), 7.65 (s, 2H), 7.40 (qnt, *J* = 7.2 Hz, 2H), 7.10 (t, *J* = 7.2 Hz, 4H), 4.47 (d, *J* = 5.2 Hz, 4H). ^13^C NMR (DMSO-*d*_6_, 50 MHz): *δ* 167.0, 166.8, 161.2 (dd, *J* = 245.5, 9.1 Hz), 138.2, 133.2, 130.3 (t, *J* = 10.2 Hz), 128.3 (d, *J* = 27.5 Hz), 112.0, 111.6 (t, *J* = 23.7 Hz), 31.2. HRMS (CI+) Calculated for C_24_H_13_F_4_N_2_O_4_ (M-2H_2_O+1) 469.0811; Found 469.0824. 

*2,5-Bis(2-chlorobenzylcarbamoyl)terephthalic Acid* ***(6H)***. White solid. 198 mg, 0.40 mmol, 86%. Mp 276–278°C. FTIR: 3265, 2880, 1662, 1638, 1515, 1396, 1333 cm^−1^. MS (EI) *m*/*z*: 429 (M^+^-2H_2_O-Cl, 20), 306 (100), 174 (50), 106 (60). MS (ESI−) *m*/*z*: 500 (M−H)^−^. ^1^H NMR (DMSO-*d*_6_, 200 MHz): *δ* 9.15 (t, *J* = 5.8 Hz, 2H), 7.85 (s, 2H), 7.62–7.24 (m, 8H), 4.51 (d, *J* = 5.8 Hz, 4H). ^13^C NMR (DMSO-*d*_6_, 50 MHz): *δ* 167.4, 167.2, 138.4, 136.0, 131.9, 130.1, 129.3, 128.8, 128.2, 127.5, 126.9, 40.6. HRMS (CI+) Calculated for C_24_H_15_Cl_2_N_2_O_4_ (M-2H_2_O+1) 465.0409; Found 465.0374. 

*2,5-Bis(2-methoxybenzylcarbamoyl)terephthalic Acid* ***(6I)***. White solid. 227 mg, 046 mmol, >98%. Mp 108–110°C FTIR: 3291, 3005, 1694, 1644, 1540, 1220 cm^−1^. MS (EI) *m*/*z*: 456 (M^+^-2H_2_O, 100), 135 (35), 121 (40), 91 (55). MS (ESI+) *m*/*z*: 493 (M+H)^+^. MS (ESI−) *m*/*z*: 491 (M−H)^−^ MS/MS 491 (3), 443 (25), 404 (100). ^1^H NMR (DMSO-*d*_6_, 200 MHz): *δ* 8.92 (t, *J* = 5.8 Hz, 2H), 7.81 (s, 2H), 7.37 (dd,*J* = 7.4, 1.4 Hz, 2H), 7.25 (td, *J* = 8.2, 1.6 Hz, 2H), 6.99 (d, *J* = 8.2 Hz, 2H), 6.93 (t, *J* = 7.4 Hz, 2H), 4.41 (d, *J* = 5.8 Hz, 4H), 3.82 (s, 6H). ^13^C NMR (DMSO-*d*_6_, 50 MHz): *δ* 167.3, 167.2, 156.6, 138.6, 133.4, 129.3, 127.7, 126.6, 120.2, 110.4, 55.4, 37.7. HRMS (CI+) Calculated for C_26_H_21_N_2_O_6_ (M-2H_2_O+1) 457.1400; Found 457.1404. 

*2,5-Bis(2-methoxyphenethylcarbamoyl)terephthalic Acid* ***(6J)***. White solid. 196 mg, 0.38 mmol, 82%. Mp 248–250°C FTIR: 3315, 3006, 2945, 1698, 1604, 1568, 1242 cm^−1^. MS (EI) *m*/*z*: 484 (M^+^-2H_2_O, 12), 174 (30), 134 (85), 91 (100). MS (ESI−) *m*/*z*: 519 (M−H)^−^, MS/MS 519 (2), 475 (42), 431 (100). ^1^H NMR (DMSO-*d*_6_, 200 MHz): *δ* 8.57 (t, *J* = 5.4 Hz, 2H), 7.67 (s, 2H), 7.23 (t, *J* = 8.4 Hz, 2H), 7.19 (d, *J* = 7.4 Hz, 2H), 6.97 (d, *J* = 8.4 Hz, 2H), 6.88 (t, *J* = 7.4 Hz, 2H), 3.80 (s, 6H), 3.40 (dt, *J* = 7.2, 5.4 Hz, 4H), 2.82 (t, *J* = 7.2 Hz, 4H). ^13^C NMR (DMSO-*d*_6_, 50 MHz): *δ* 167.2, 166.9, 157.3, 138.6, 133.2, 130.1, 128.3, 127.6, 127.3, 120.3, 110.7, 55.3, 40.8, 29.5. HRMS (CI+) Calculated for C_28_H_25_N_2_O_6_ (M-2H_2_O+1) 485.1713; Found 485.1676. 

*2,5-Bis(3-phenylpropylcarbamoyl)terephthalic Acid* ***(6K)***. White solid. 194 mg, 0.40 mmol, 86%. Mp 216–218°C. FTIR: 3338, 3065, 2943, 1699, 1615, 1595, 1206 cm^−1^. MS (EI) *m*/*z*: 452 (M^+^-2H_2_O, 25), 348 (23), 244 (20), 174 (40), 117 (100), 91 (65). MS (ESI+) *m*/*z*: 982 (2 M+Na)^+^, 511 (M+Na)^+^. MS (ESI−) *m*/*z*: 487 (M−H)^−^ MS/MS 487(4), 443(50), 399 (100). ^1^H NMR (DMSO-*d*_6_, 200 MHz): *δ* 13.33 (sa, 2H), 8.53 (t, *J* = 5.6, 2H), 7.73 (s, 2H), 7.31−7.18 (m, 10H), 3.23 (dt, *J* = 7.0, 5.8 Hz, 4H), 2.65 (dd, *J* = 8.2, 7.0 Hz, 4H), 1.81 (qnt, *J* = 7.6, 7.0 Hz, 4H). ^13^C NMR (DMSO-*d*_6_, 50 MHz): *δ* 167.1, 167.0, 141.9, 138.8, 133.1, 128.4, 128.3, 125.7, 39.5, 32.6, 30.7. HRMS (CI+) Calculated for C_28_H_25_N_2_O_4_ (M-2H_2_O+1) 453.1814; Found 453.1801. 

*2,5-Bis(2-phenoxyethylcarbamoyl)terephthalic Acid* ***(6L)***. White solid. 161 mg, 0.33 mmol, 71%. Mp 200–202°C. MS (ESI+) *m*/*z*: 515 (M+H)^+^. MS (ESI−) *m*/*z*: 491 (M−H)^−^. FTIR: 3330, 3069, 2943, 1704, 1625, 1586, 1218 cm^−1^. ^1^H NMR (DMSO-*d*_6_, 200 MHz): *δ* 13.37 (brs, 2H), 8.78 (t, *J* = 5.2 Hz, 2H), 7.75 (s, 2H), 7.29 (t, *J* = 7.2 Hz, 4H), 6.95–7.00 (m, 6H), 4.10 (t, *J* = 5.6 4H), 3.57 (q, *J* = 5.2 Hz, 4H). ^13^C NMR (DMSO-*d*_6_, 50 MHz): *δ* 167.5, 166.9, 158.4, 138.4, 133.1, 129.5, 128.3, 120.6, 114.5, 65.7, 39.1. HRMS (CI+) Calculated for C_26_H_25_N_2_O_8_-2H_2_O 457.1400; Found 457.1382. 

*2,5-Bis(naphthalen-1-ylcarbamoyl)terephthalic Acid* ***(6M)***. White solid. 194 mg, 0.39 mmol, 84%. Mp 362–364°C. FTIR: 3242, 3050, 1695, 1650, 1524, 1296 cm^−1^. EIMS *m*/*z*: 468 (M^+^-2H_2_O, 5), 343 (10), 174 (45), 143 (100), 115 (50). MS (ESI+) *m*/*z*: 505 (M+H)^+^. MS (ESI−) *m*/*z*: 503 (M−H)^−^ MS/MS 503 (1), 460 (10), 416 (100). ^1^H NMR (DMSO-*d*_6_, 200 MHz): *δ* 10.63 (s, 2H), 8.23 (d, *J* = 7.8 Hz, 2H), 8.18 (s, 2H), 7.98 (dd, *J* = 6.0, 3.0 Hz, 2H), 7.87 (d, *J* = 8.1 Hz, 2H), 7.79 (d, *J* = 7.0, Hz, 2H), 7.62–7.65 (m, 6H). ^13^C NMR (DMSO-*d*_6_, 50 MHz): *δ* 167.0, 166.8, 139.4, 133.8, 133.5, 132.9, 129.3, 129.2, 128.7, 127.8, 126.4, 125.9, 123.2, 123.1, 122.5. HRMS (CI+) Calculated for C_30_H_17_N_2_O_4_ (M-2H_2_O+1) 469.1188; Found 469.1217. 

*2,5-Bis(naphthalen-1-ylmethylcarbamoyl)terephthalic Acid* ***(6N)***. White solid. 240 mg, 0.45 mmol, 98%. Mp 270–272°C. FTIR: 3342, 3048, 2916, 1716, 1627, 1580, 1567, 1214 cm^−1^. MS (EI) *m*/*z*: 496 (M^+^-2H_2_O, 100), 248 (10), 141 (55), 115 (30). MS (ESI+) *m*/*z*: 555 (M+Na)^+^; MS (ESI-) *m*/*z*: 531 (M−H)^−^.^1^H NMR (DMSO-*d*_6_, 400 MHz): *δ* 13.42 (brs, 2H), 9.19 (t, *J* = 5.6 Hz, 2H), 8.16 (d, *J* = 8.4 Hz, 2H), 7.96 (d, *J* = 8.0 Hz, 2H), 7.87 (d, *J* = 8.4 Hz, 2H), 7.77 (s, 2H), 7.59 (t, *J* = 8.4 Hz, 4H), 7.53 (t, *J* = 8.0 Hz, 2H), 7.49 (t, *J* = 8.0 Hz, 2H), 4.91 (d, *J* = 5.6 Hz, 4H). ^13^C NMR (DMSO-*d*_6_, 50 MHz): *δ* 167.2, 166.9, 138.4, 134.2, 133.4, 133.2, 130.9, 128.5, 127.6, 126.3, 125.8, 125.7, 125.4, 123.6, 115.7, 40.9. HRMS (CI+) Calculated for C_32_H_21_N_2_O_4_ (M-2H_2_O+1) 497.1501; Found 497.1513.

## Supplementary Material

Supplementary Material: ^1^H and ^13^C NMR spectra of all compunds are included.

## Figures and Tables

**Table 1:** Yields for bis(carbamoylcarboxylic) acids.

Amine	Products	
	**3**	**6**
**A**	92	>98
**B**	60	68
**C**	63	89
**D**	>98	82
**E**	83	63
**F**	85	90
**G**	>98	80
**H**	>98	86
**I**	>98	>98
**J**	96	82
**K**	63	86
**L**	74	71
**M**	>98	84
**N**	98	98

## Computational Details

All geometry optimizations and frequency calculations were carried out with DFT method and the nonlocal correlation B3LYP with a 6-31+G(d,p) basis set using *Gaussian 03* package in the gas-phase [37, 38].
