# Supplementary material for: A Novel and Highly Regioselective Synthesis of New Carbamoylcarboxylic Acids from Dianhydrides
Source: ScientificWorldJournal. 2014 Jan 6;2014:725981. doi: 10.1155/2014/725981 (PMC3913102; doi:10.1155/2014/725981)
Supplement: Supplementary file 1 — Supplementary Material: 1H and 13C NMR spectra of all compunds are included. [file 725981.f1.pdf]

Supporting Information

A novel and highly regioselective synthesis of new carbamoylcarboxylic acids from dianhydrides

Adrián Ochoa-Teran[1]*, Jesús Estrada-Manjarrez[2], Marisela Martínez-Quiroz[1], Marco Antonio Landey-Álvarez[1], Eleazar Alcántar Zavala[3], Georgina Pina-Luis[1], Hisila Santacruz-Ortega[4], Luis Enrique Gómez-Pineda[1], José Z. Ramírez[4], Daniel Chávez[1], Julio Montes Ávila[3], Victoria Labastida-Galván[5] and Mario Ordoñez[5]

[1] Centro de Graduados e Investigación, Instituto Tecnológico de Tijuana. Tijuana, B. C., 22510, México.
[2] Departamento de Ingeniería Bioquímica, Instituto Tecnológico de Culiacán. Culiacán, Sin., 80220. México.
[3] Facultad de Ciencias Químico Biológicas, Universidad Autónoma de Sinaloa, Culiacán, Sin. 80010, México.
[4] Departamento de Polímeros y Materiales, Universidad de Sonora, Hermosillo, Son. 83000, México.
[5] Centro de Investigaciones Químicas, Universidad Autónoma del Estado de Morelos. Cuernavaca, Mor. 62209. México.

Content		Page No.

II.	Selected NMR spectra of compounds **3**	**S2**
III.	Selected NMR spectra of compounds **6**	**S20**

I. Selected NMR spectra of compounds 3

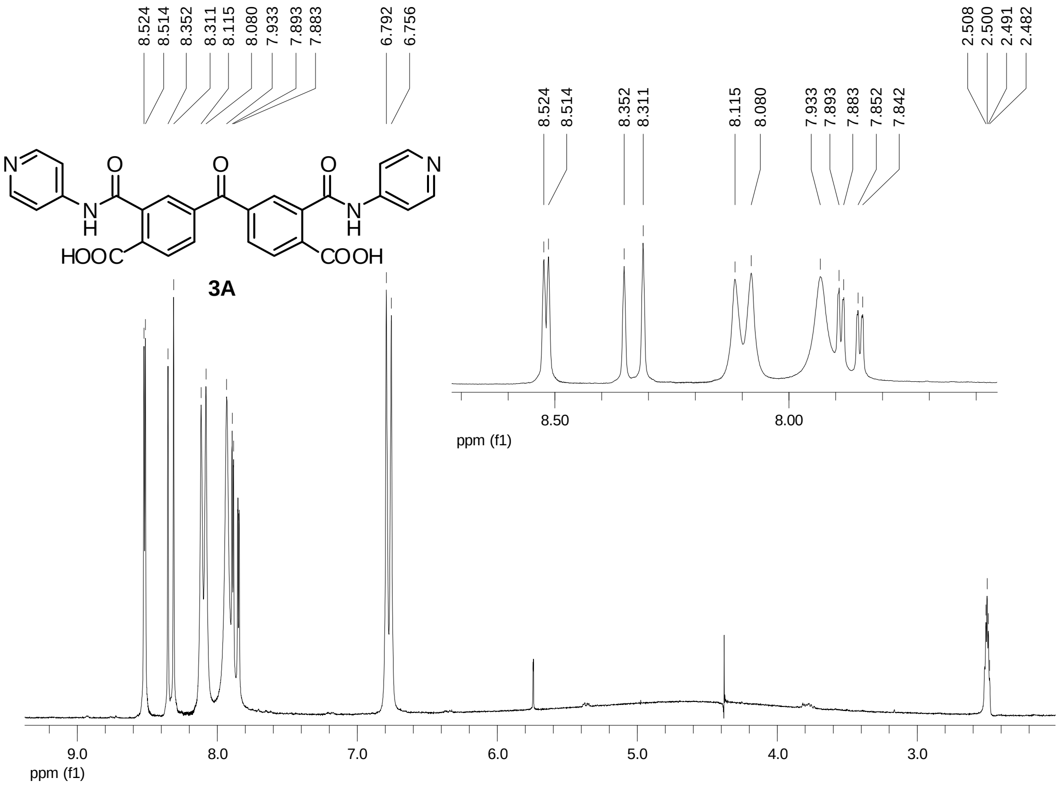

With two drops of D_2O

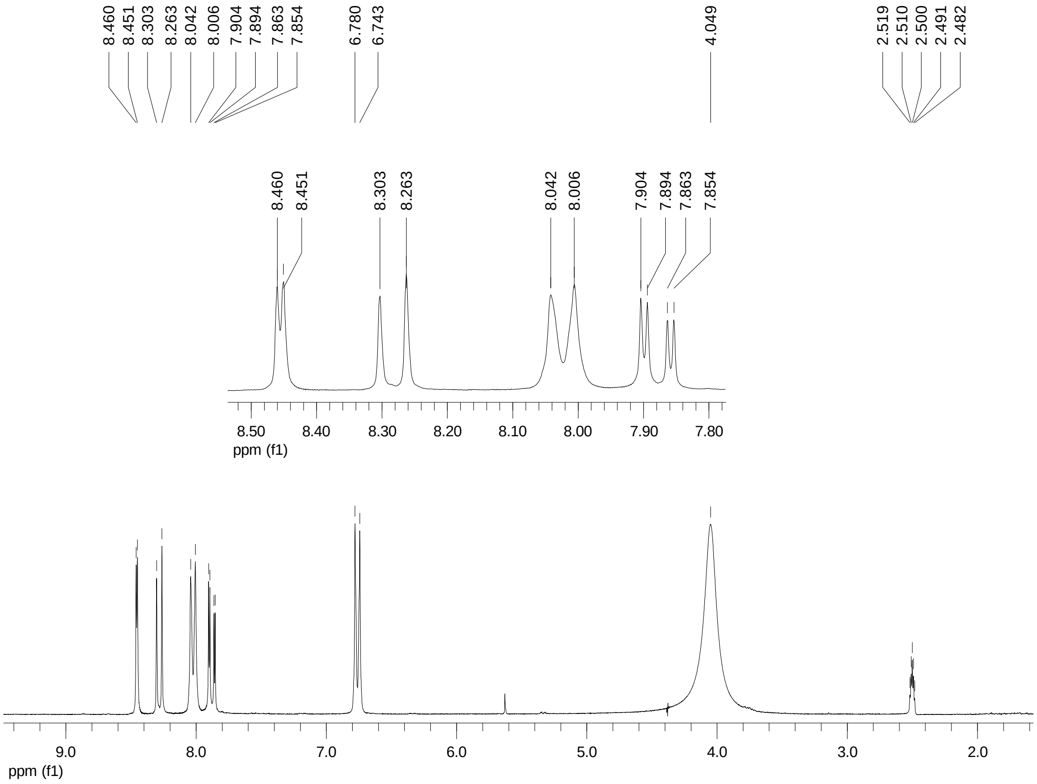

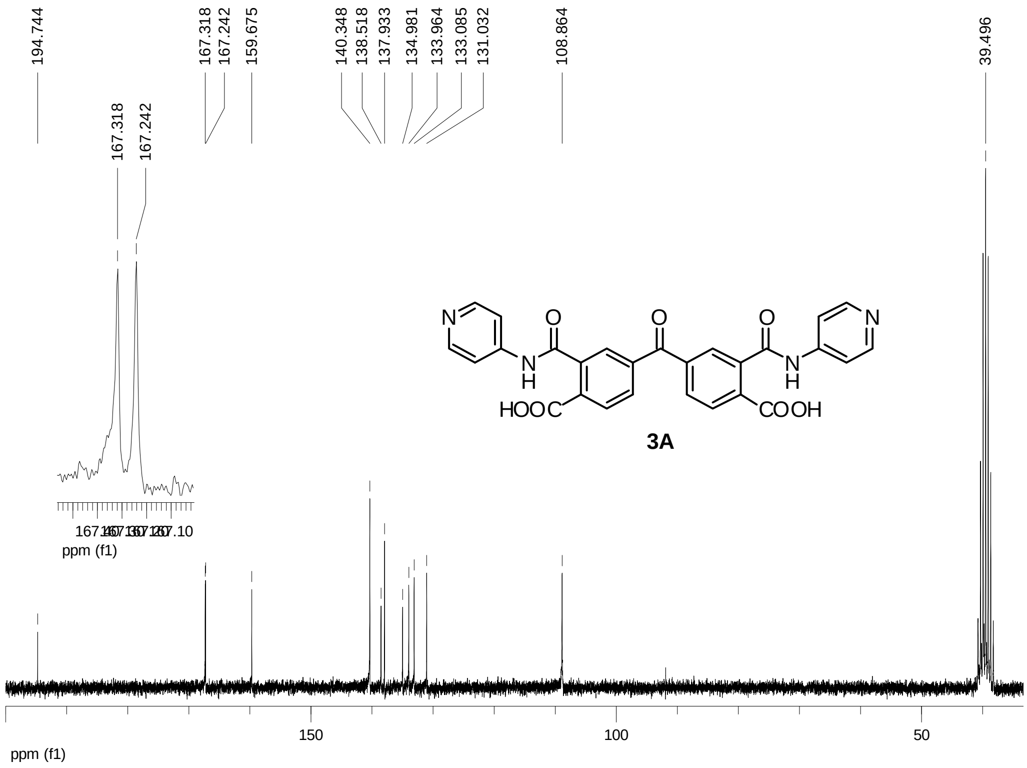
194.744
167.318
167.242
159.675
140.348
138.518
137.933
134.981
133.964
133.085
131.032
108.864
39.496
167.318
167.242
ppm (f1)
HOOC
COOH
3A
150
100
50
ppm (f1)

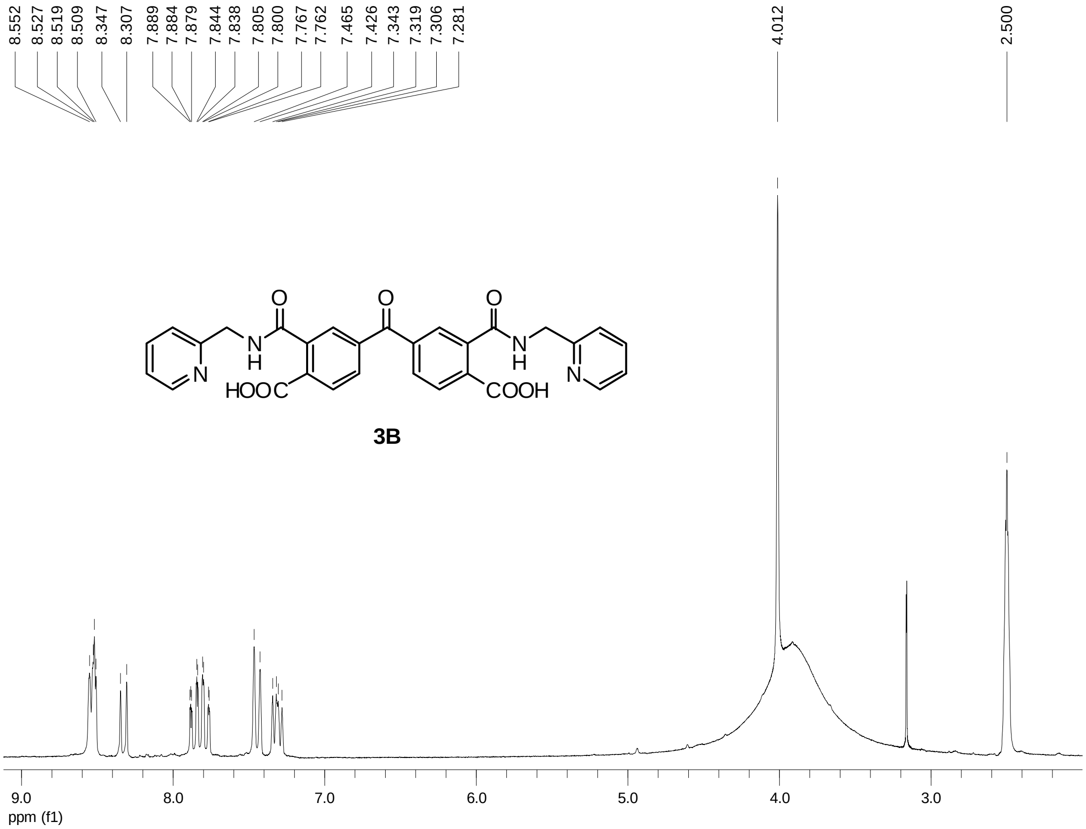
8.552 8.527 8.519 8.509 8.347 8.307 7.889 7.884 7.879 7.844 7.838 7.805 7.800 7.767 7.762 7.465 7.426 7.343 7.319 7.306 7.281
4.012
2.500
O O O
N H N H
N N
HOOC COOH
3B
9.0 8.0 7.0 6.0 5.0 4.0 3.0
ppm (f1)

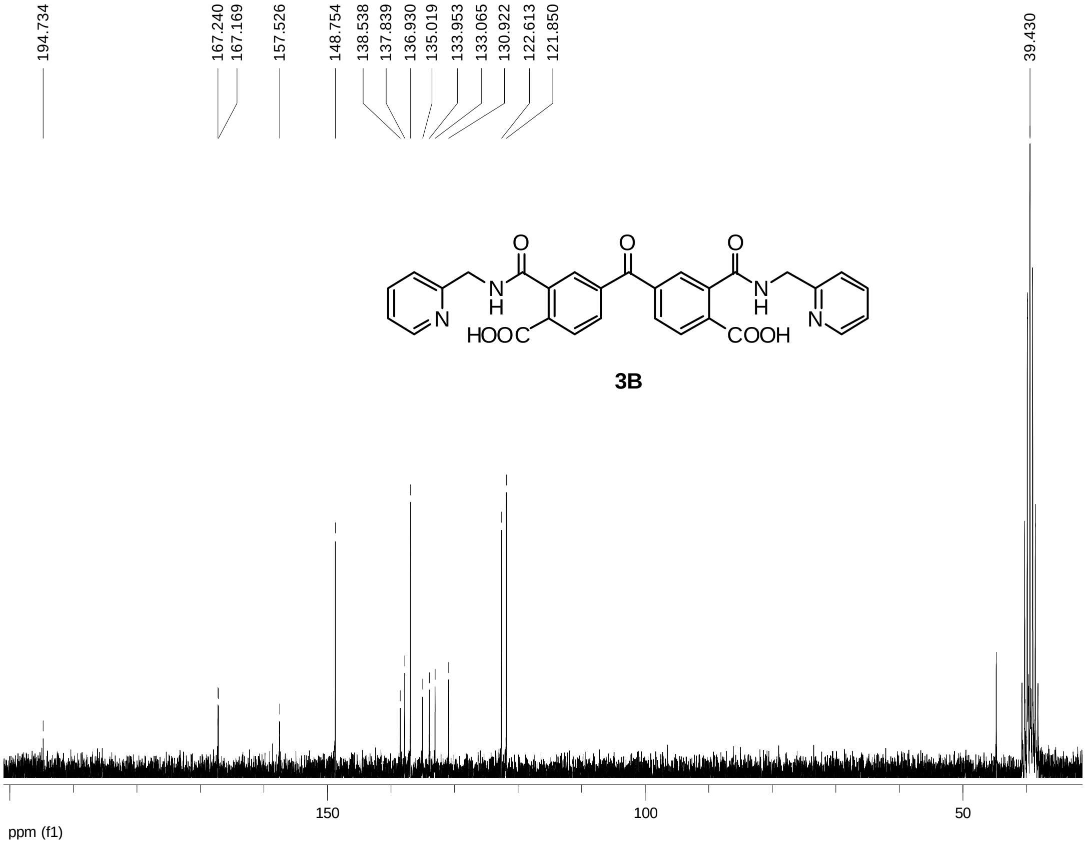
194.734
167.240 167.169
157.526
148.754 138.538 137.839 136.930 135.019 133.953 133.065 130.922 122.613 121.850
39.430
O O O
N H N H
N N
HOOC COOH
3B
150 100 50
ppm (f1)

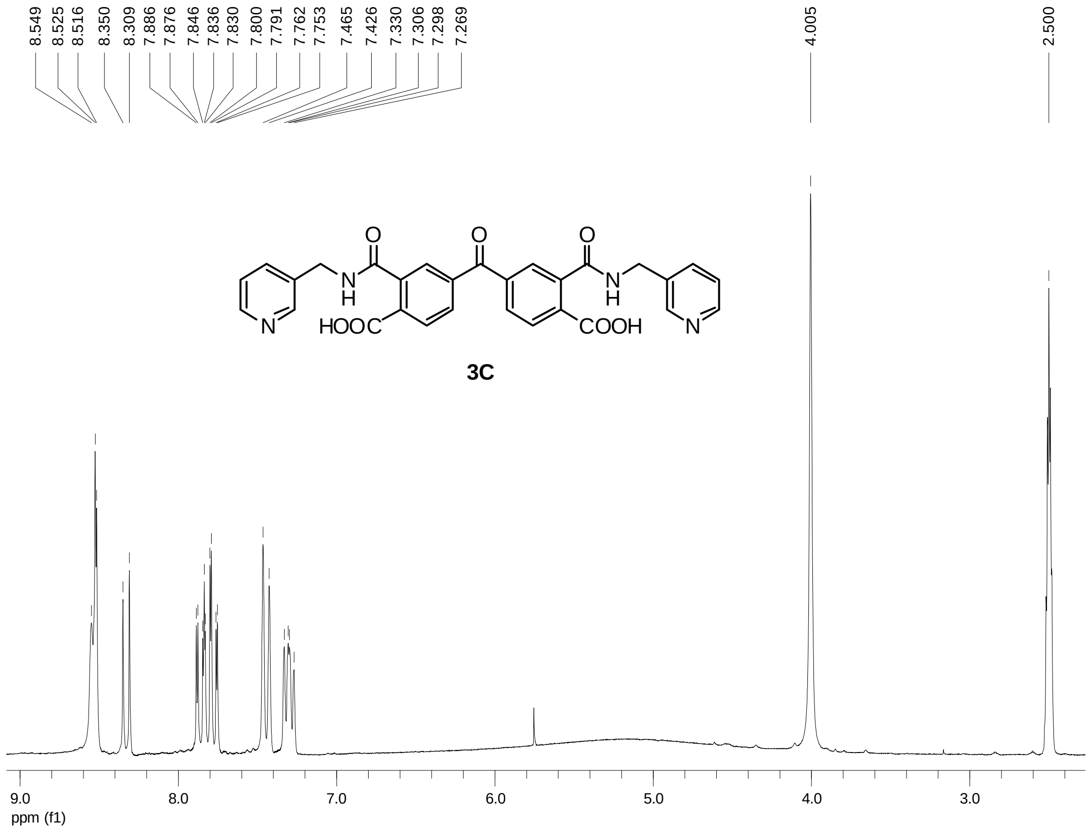
8.549
8.525
8.516
8.350
8.309
7.886
7.876
7.846
7.836
7.830
7.800
7.791
7.762
7.753
7.465
7.426
7.330
7.306
7.298
7.269
4.005
2.500
3C
9.0
8.0
7.0
6.0
5.0
4.0
3.0
ppm (f1)

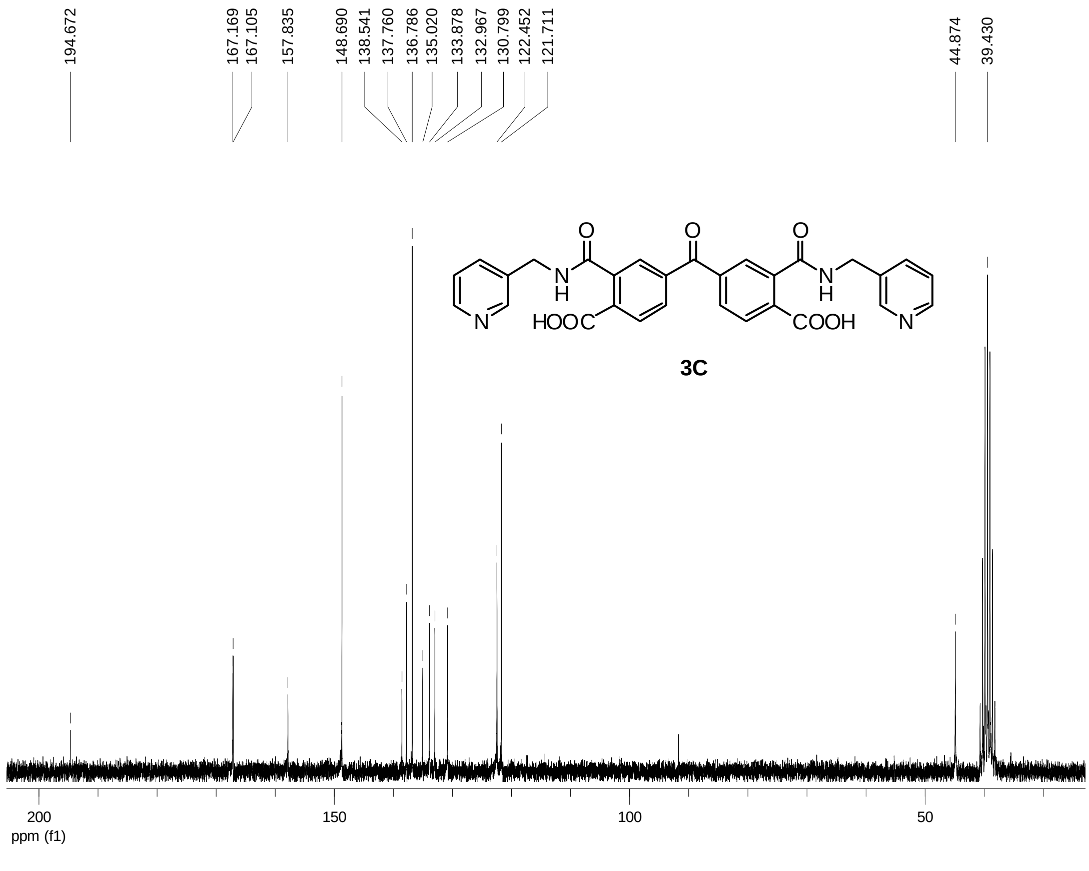
194.672
167.169
167.105
157.835
148.690
138.541
137.760
136.786
135.020
133.878
132.967
130.799
122.452
121.711
44.874
39.430
3C
200
150
100
50
ppm (f1)

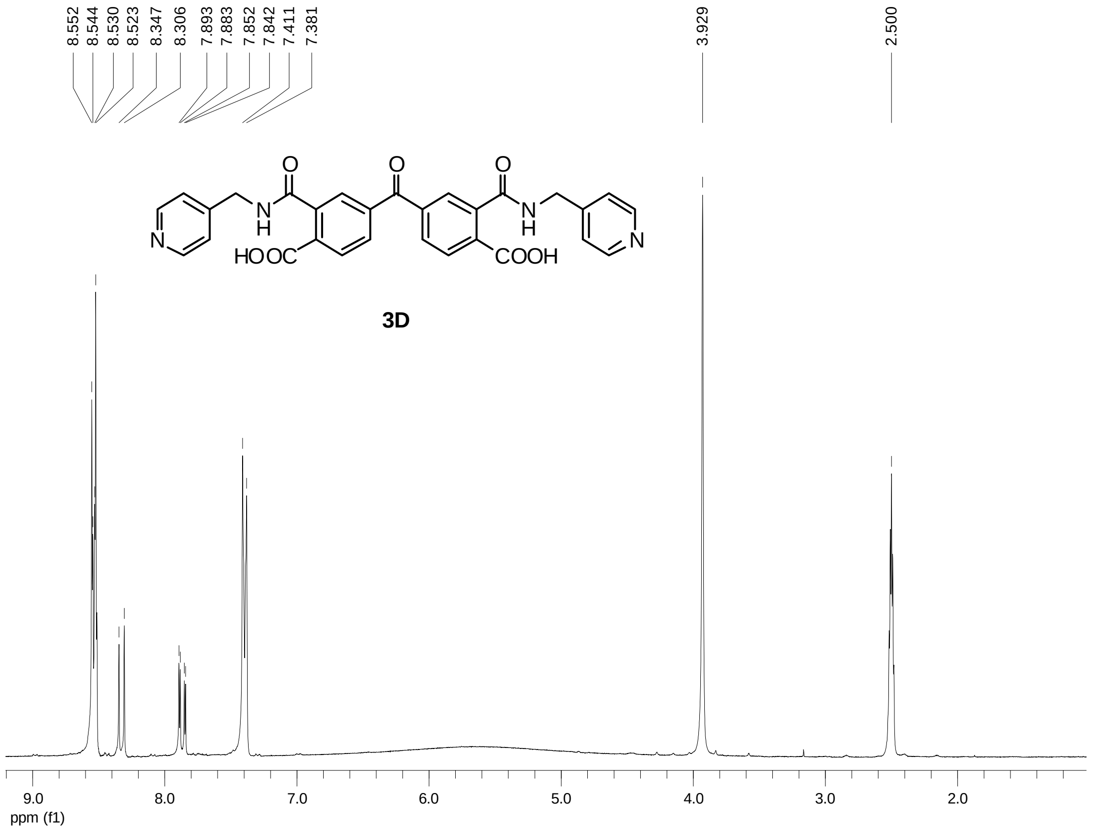
8.552
8.544
8.530
8.523
8.347
8.306
7.893
7.883
7.852
7.842
7.411
7.381
3.929
2.500
HOOC
COOH
3D
9.0
8.0
7.0
6.0
5.0
4.0
3.0
2.0
ppm (f1)

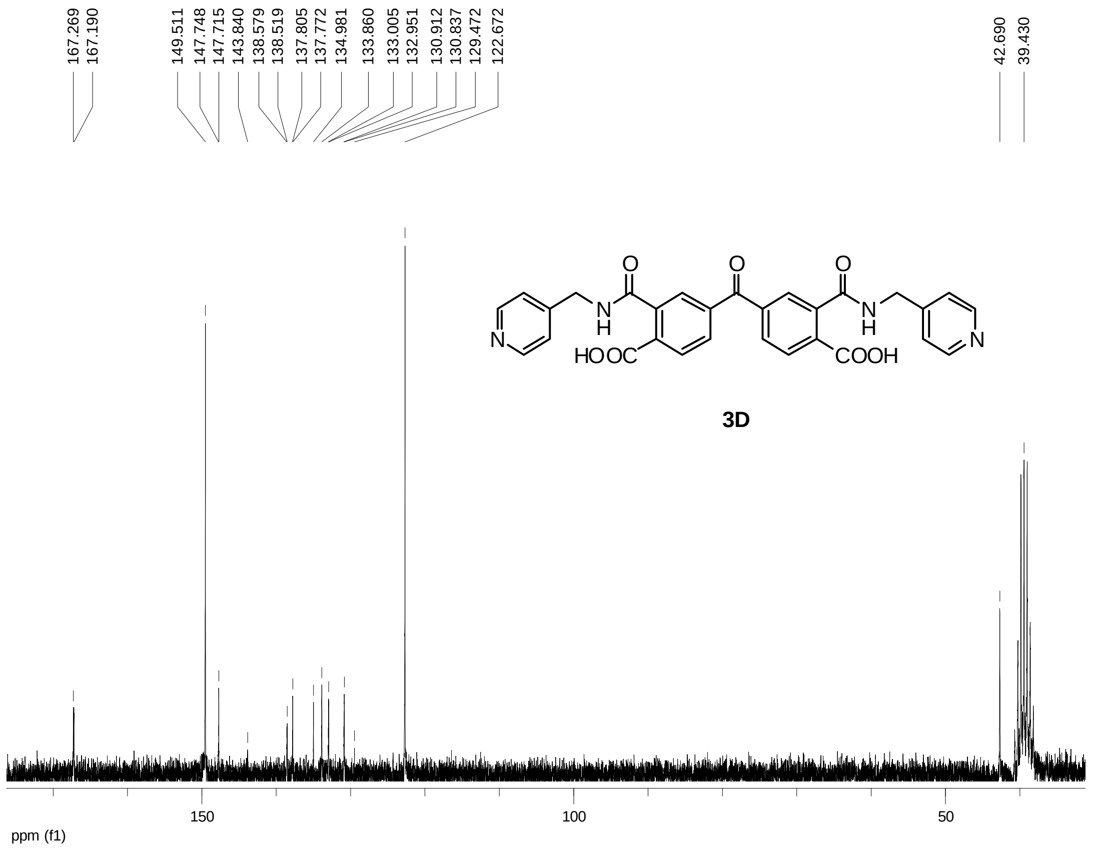
167.269
167.190
149.511
147.748
147.715
143.840
138.579
138.519
137.805
137.772
134.981
133.860
133.005
132.951
130.912
130.837
129.472
122.672
42.690
39.430
HOOC
COOH
3D
150
100
50
ppm (f1)

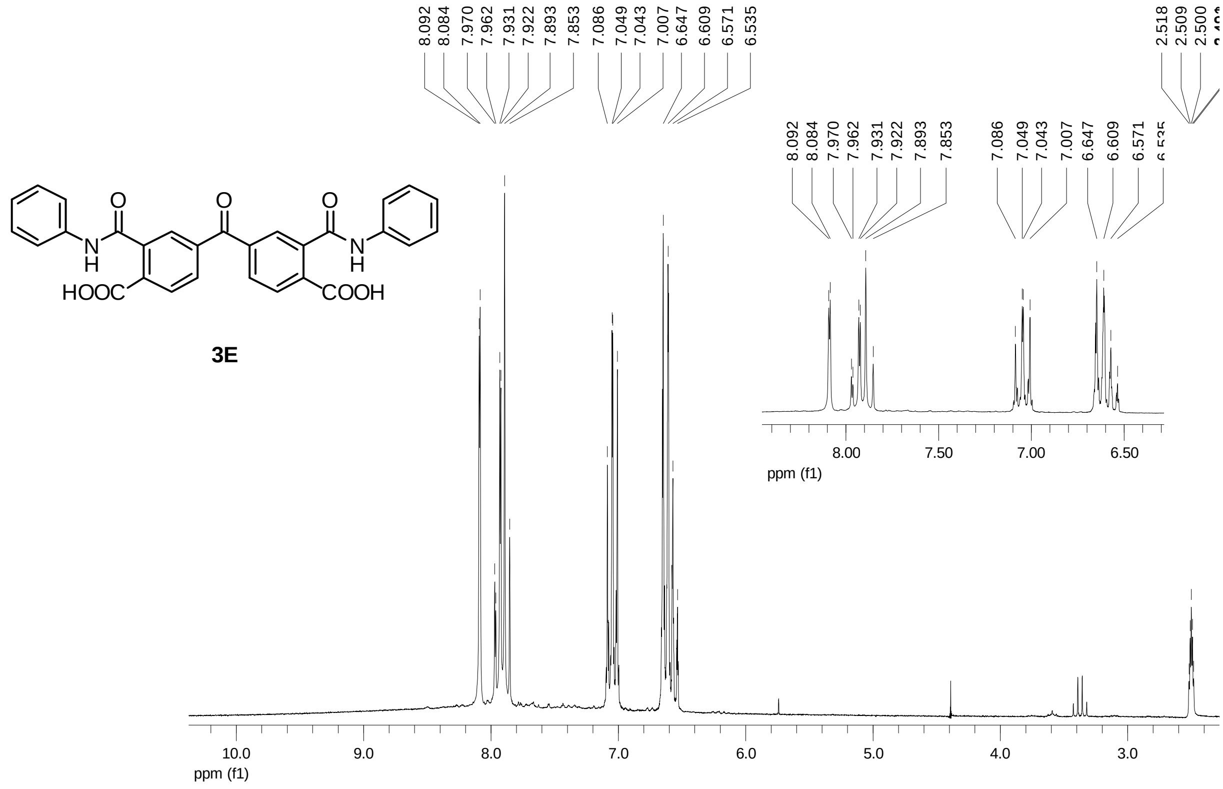

3E

With one drop of D_2O

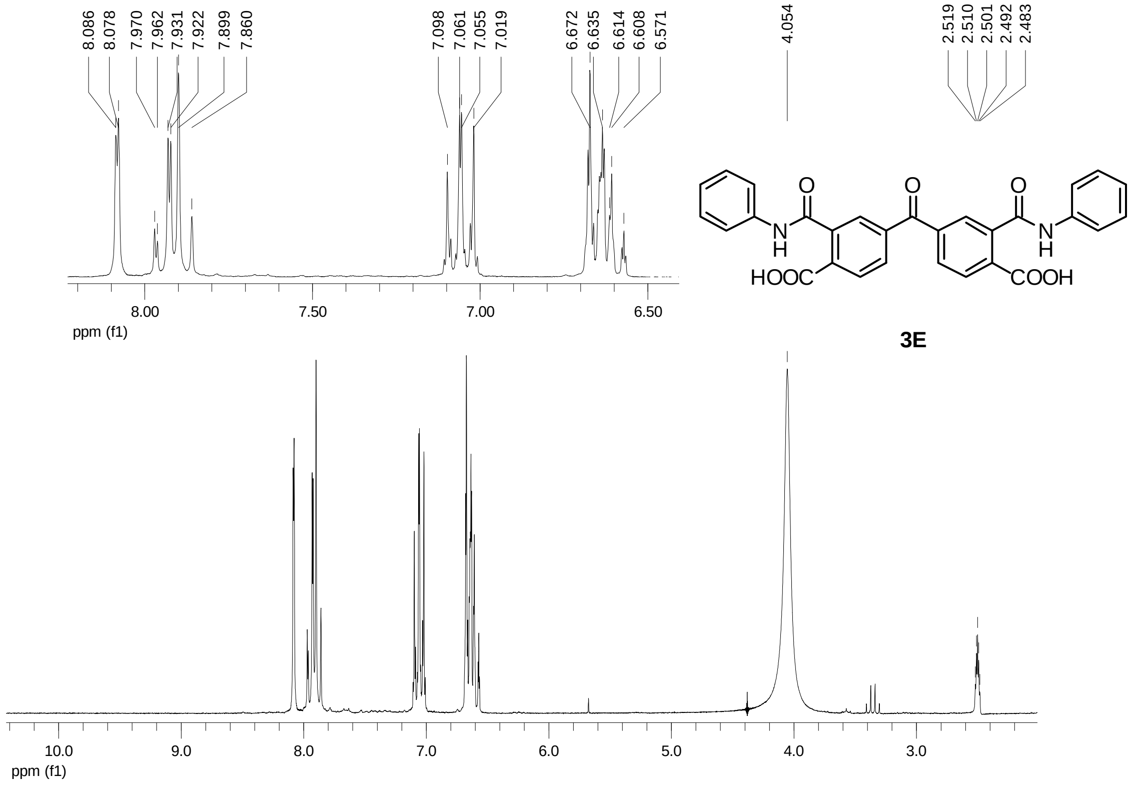

3E

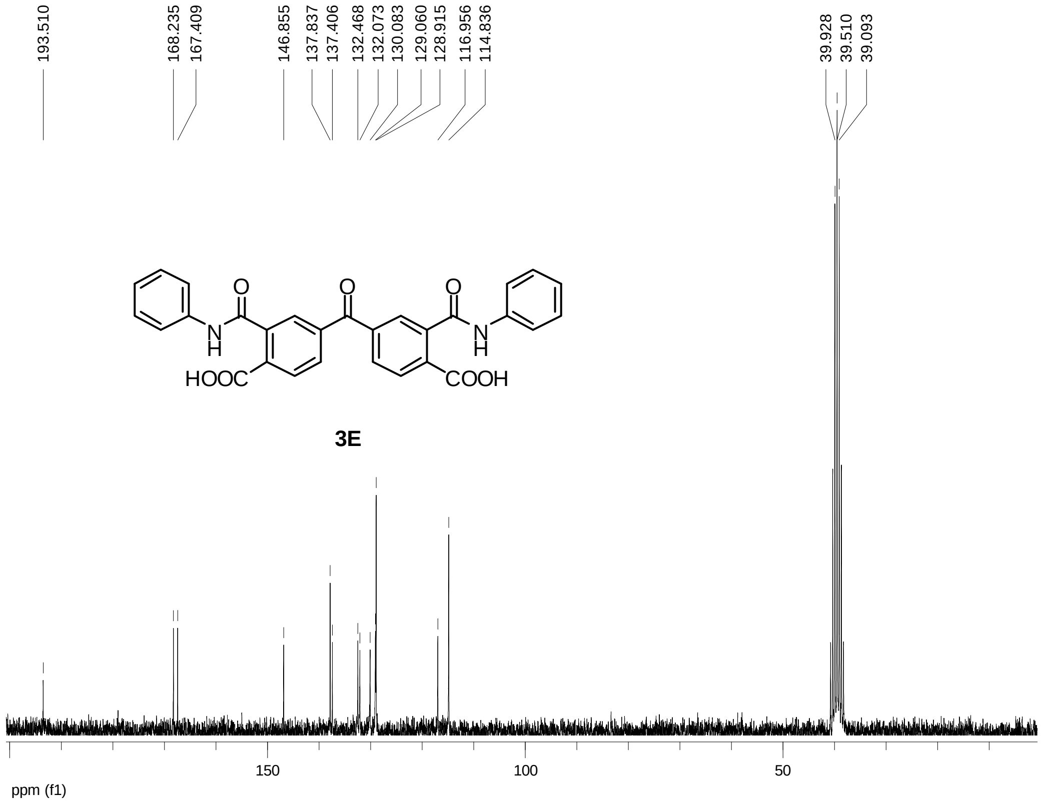
193.510
168.235
167.409
146.855
137.837
137.406
132.468
132.073
130.083
129.060
128.915
116.956
114.836
39.928
39.510
39.093
O
O
O
N
H
N
H
HOOC
COOH
3E
150
100
50
ppm (f1)

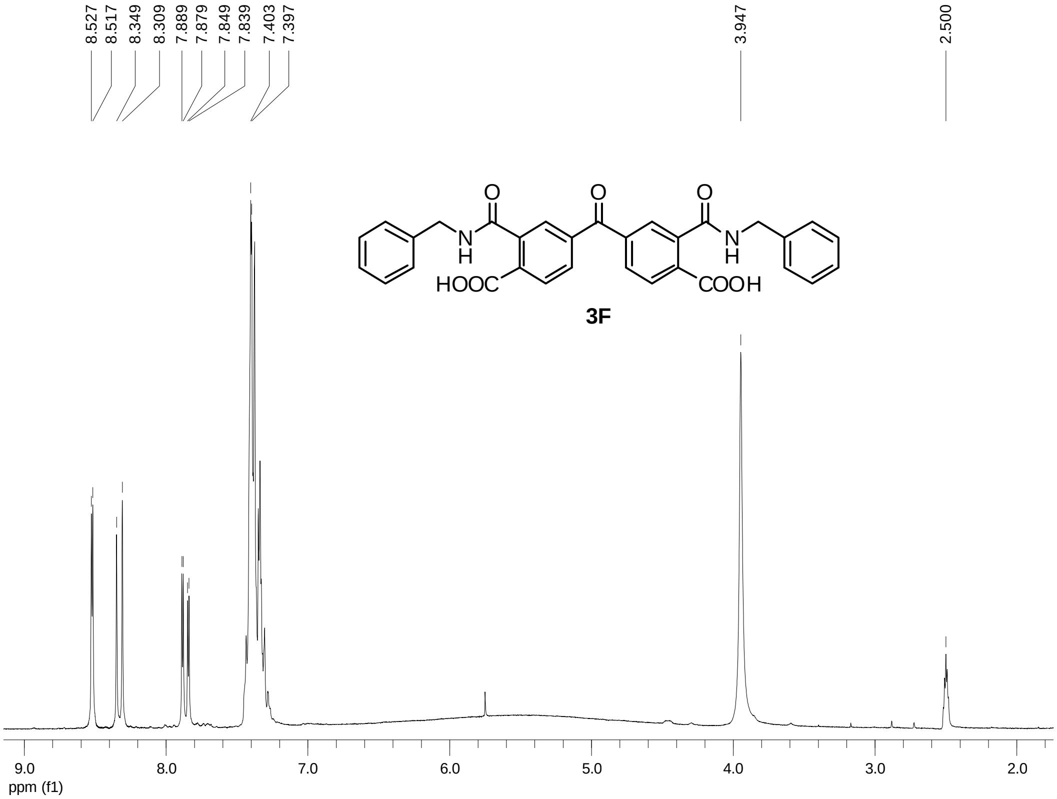

With one drop of D_2O

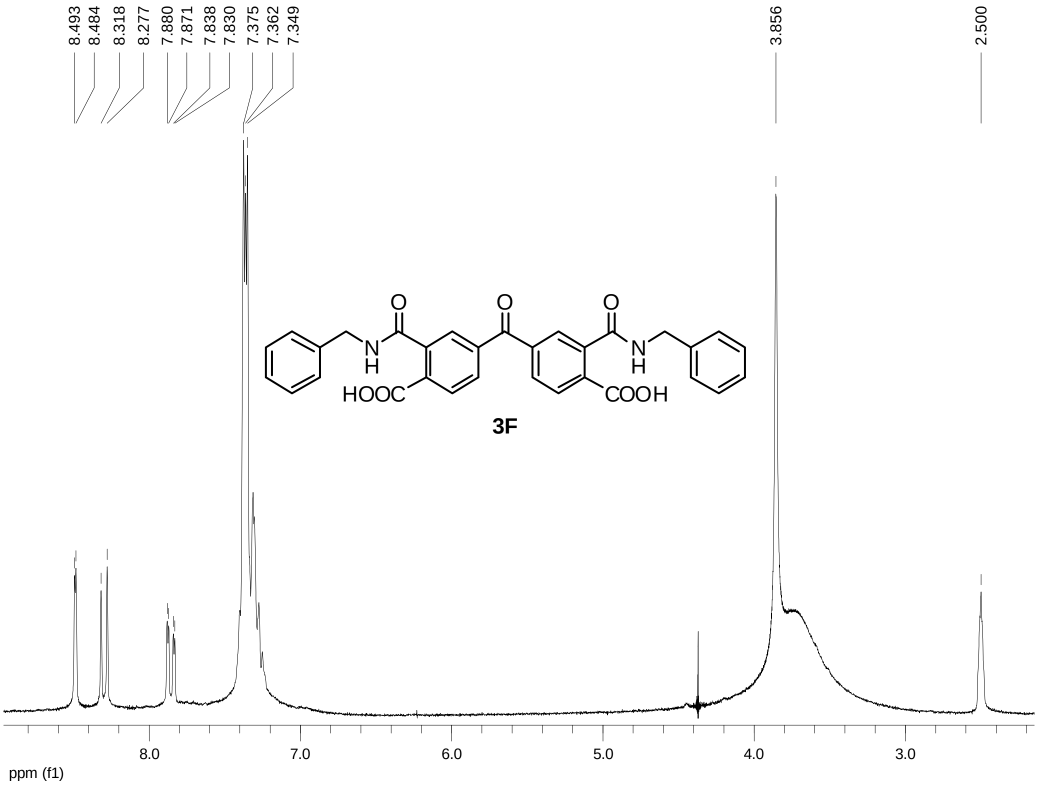

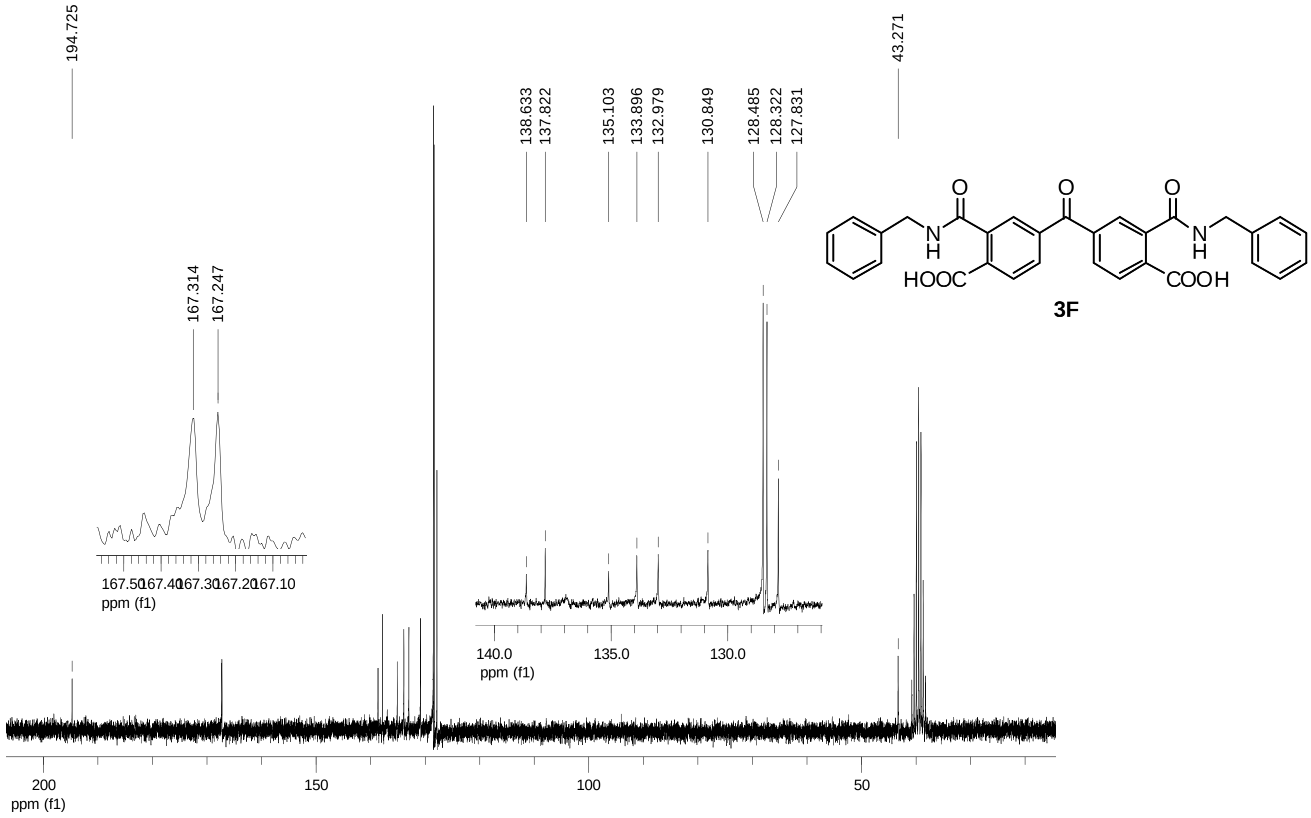
194.725
138.633
137.822
135.103
133.896
132.979
130.849
128.485
128.322
127.831
43.271
167.314
167.247
167.50 167.40 167.30 167.20 167.10
ppm (f1)
140.0 135.0 130.0
ppm (f1)
200 150 100 50
ppm (f1)
HOOC
COOH
3F

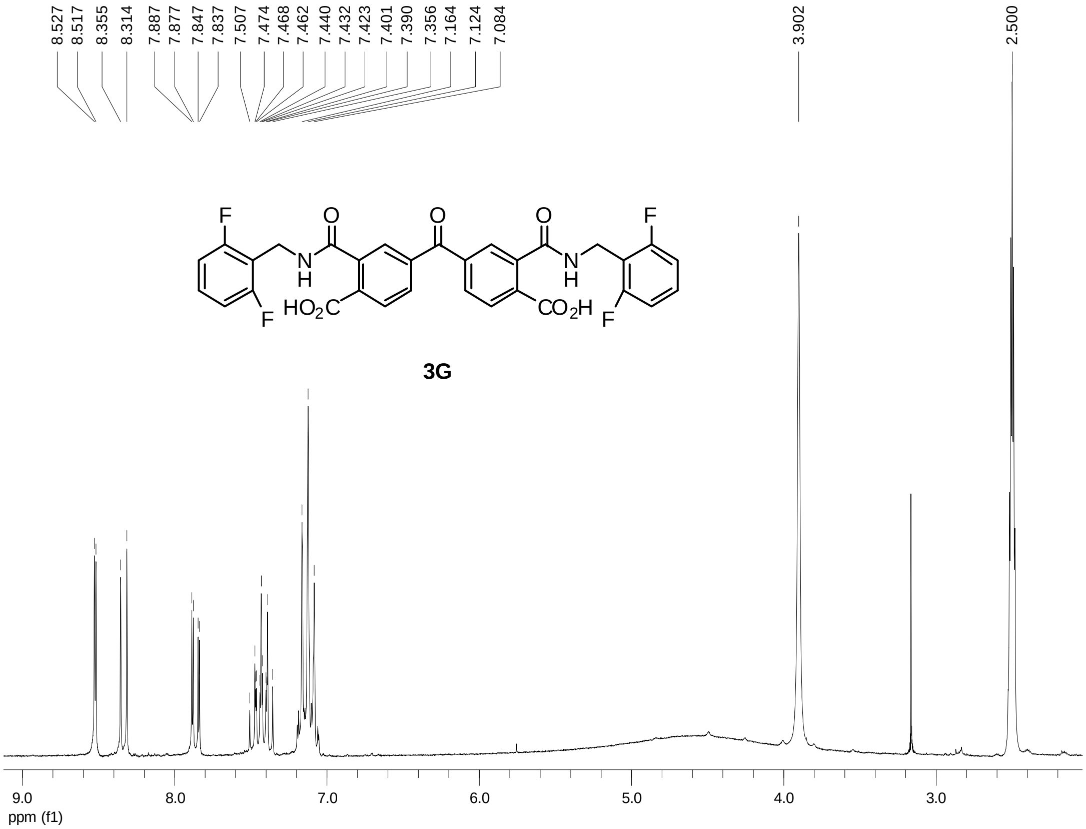
8.527
8.517
8.355
8.314
7.887
7.877
7.847
7.837
7.507
7.474
7.468
7.462
7.440
7.432
7.423
7.401
7.390
7.356
7.164
7.124
7.084
3.902
2.500
3G
9.0
8.0
7.0
6.0
5.0
4.0
3.0
ppm (f1)

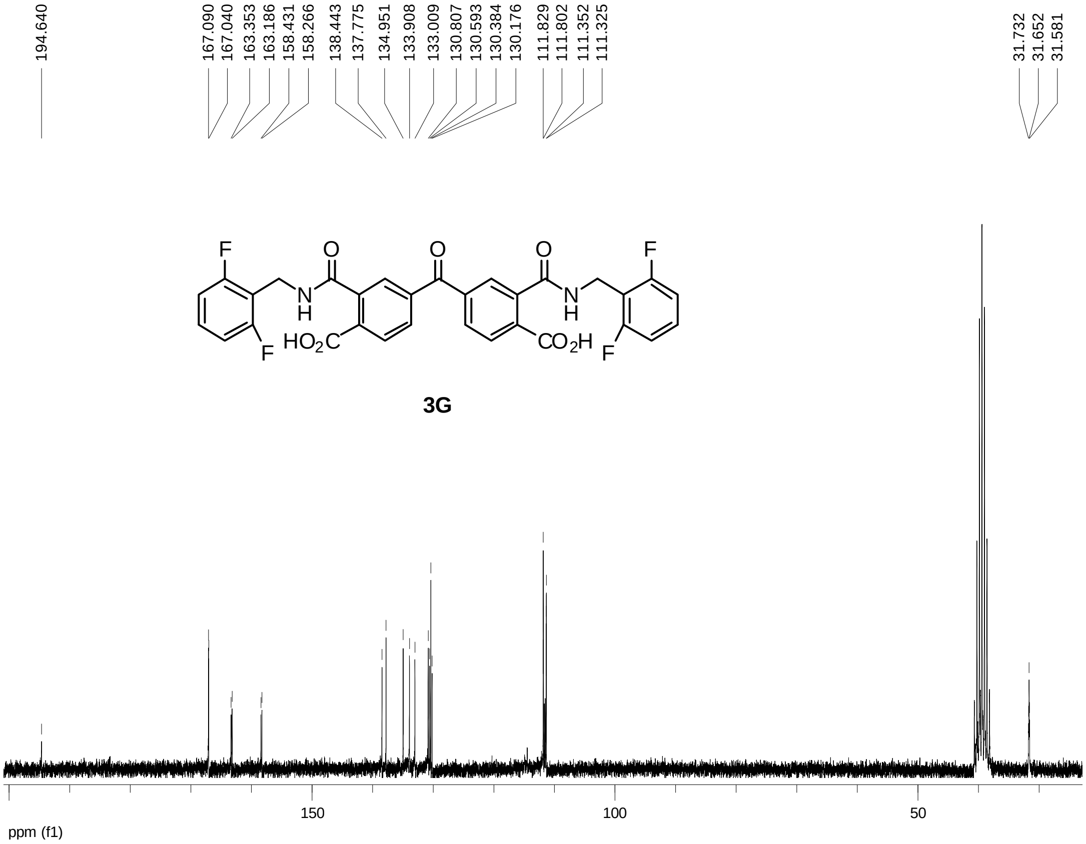
194.640
167.090
167.040
163.353
163.186
158.431
158.266
138.443
137.775
134.951
133.908
133.009
130.807
130.593
130.384
130.176
111.829
111.802
111.352
111.325
31.732
31.652
31.581
3G
150
100
50
ppm (f1)

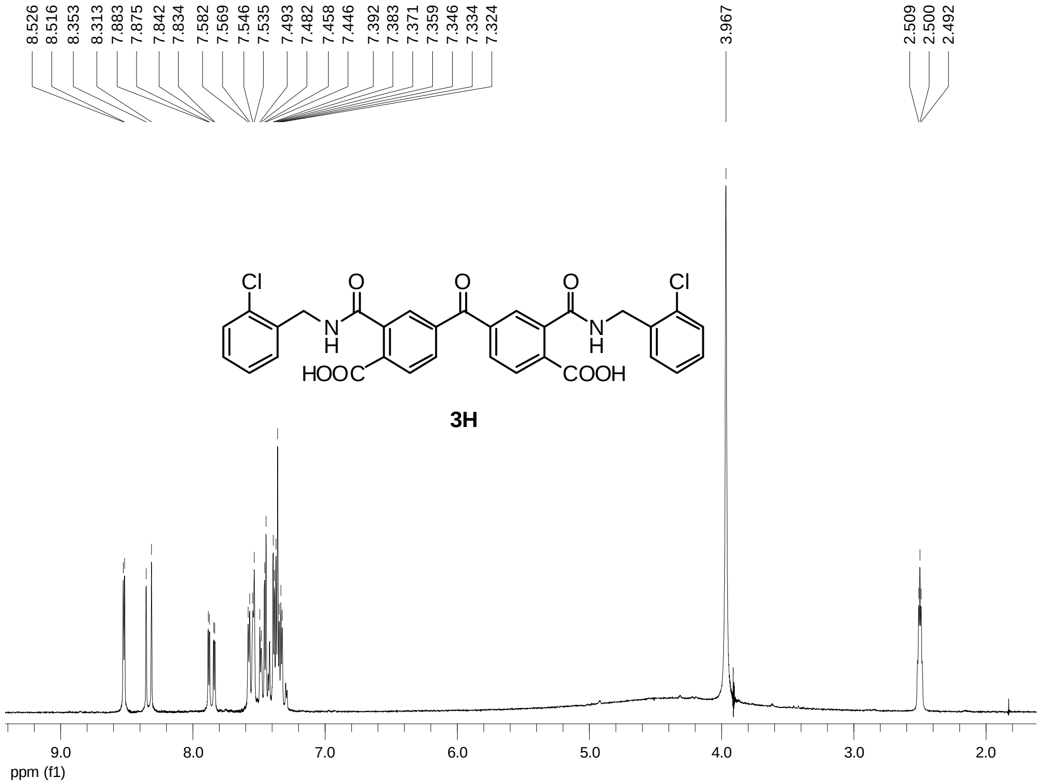

With two drops of D_2O added

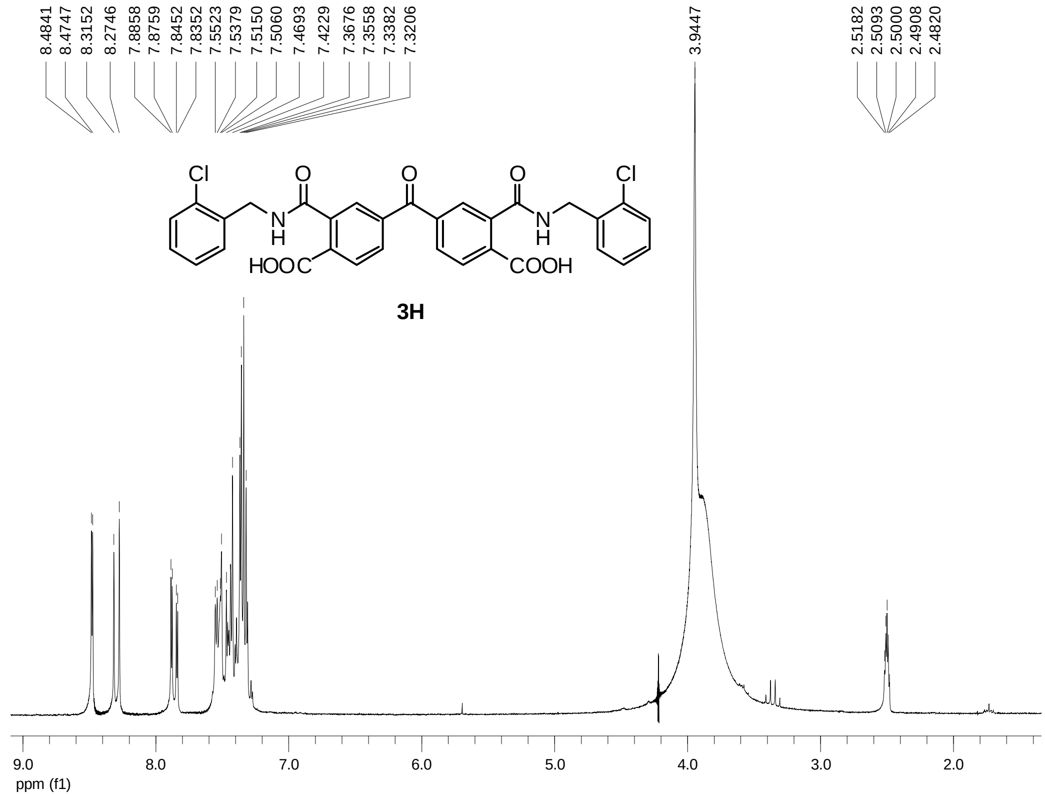

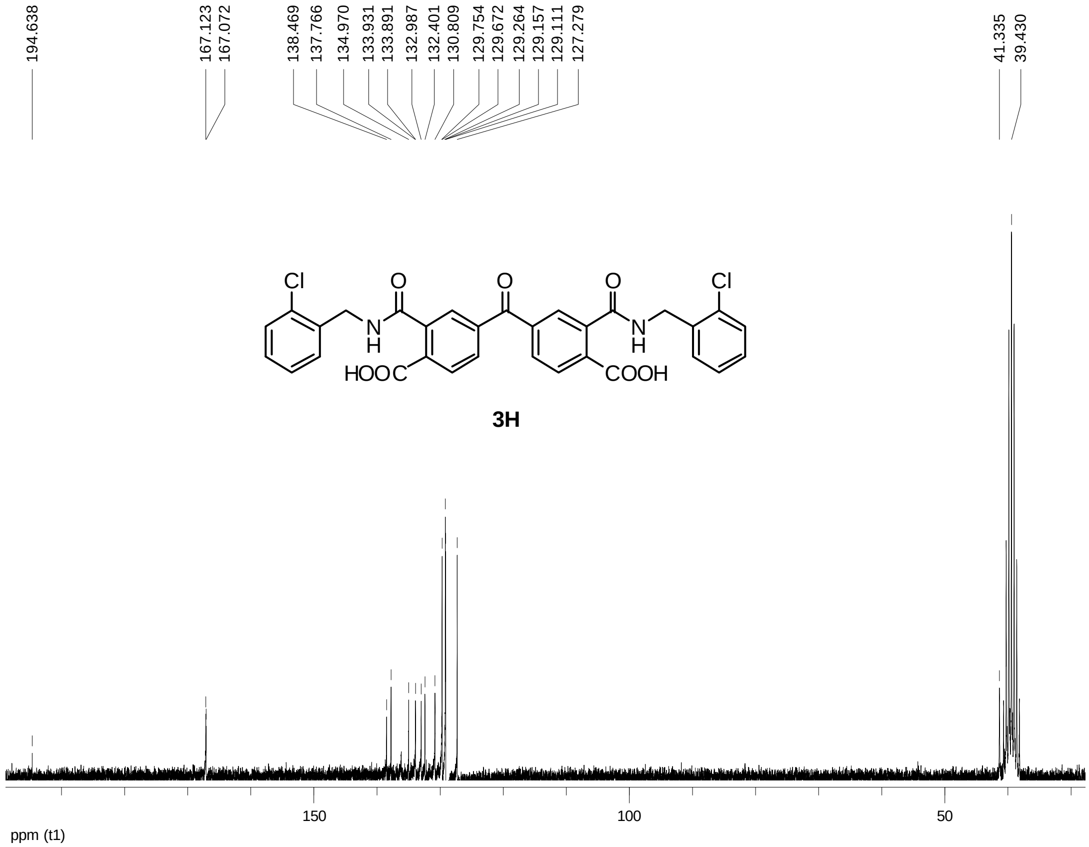
194.638
167.123
167.072
138.469
137.766
134.970
133.931
133.891
132.987
132.401
130.809
129.754
129.672
129.264
129.157
129.111
127.279
41.335
39.430
Cl
O
O
O
Cl
N
H
N
H
HOOC
COOH
3H
150
100
50
ppm (t1)

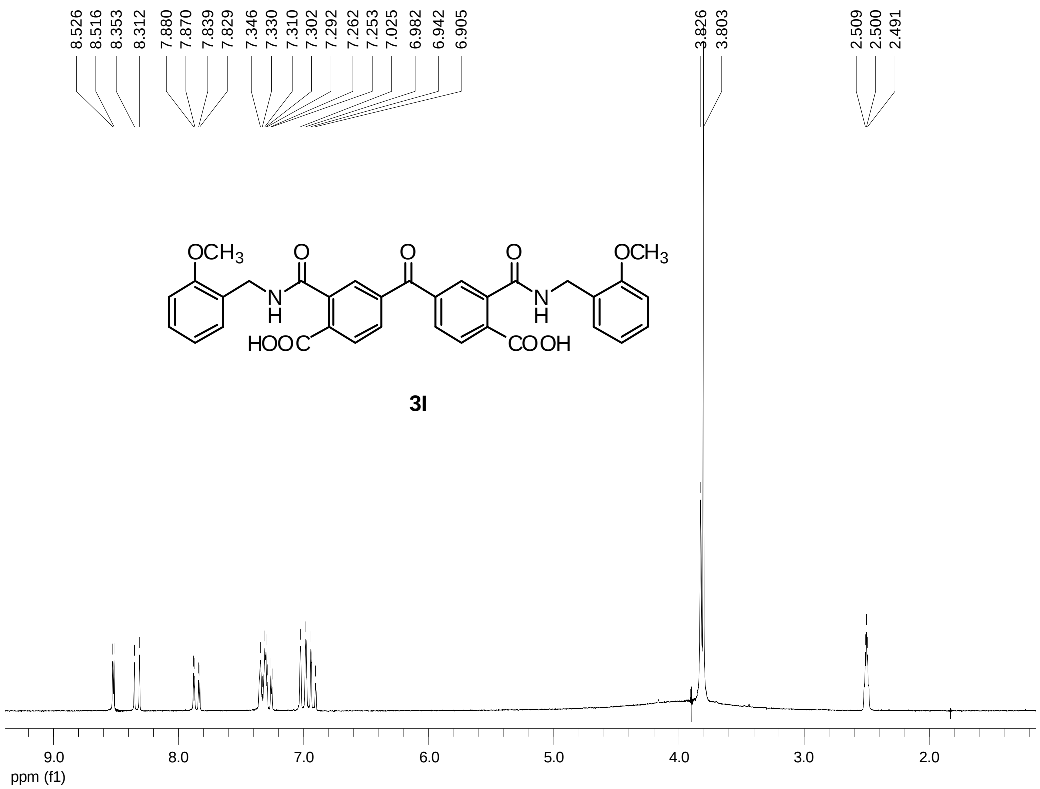
8.526
8.516
8.353
8.312
7.880
7.870
7.839
7.829
7.346
7.330
7.310
7.302
7.292
7.262
7.253
7.025
6.982
6.942
6.905
3.826
3.803
2.509
2.500
2.491
OCH3
O
O
O
OCH3
N
H
N
H
HOOC
COOH
3l
9.0
8.0
7.0
6.0
5.0
4.0
3.0
2.0
ppm (f1)

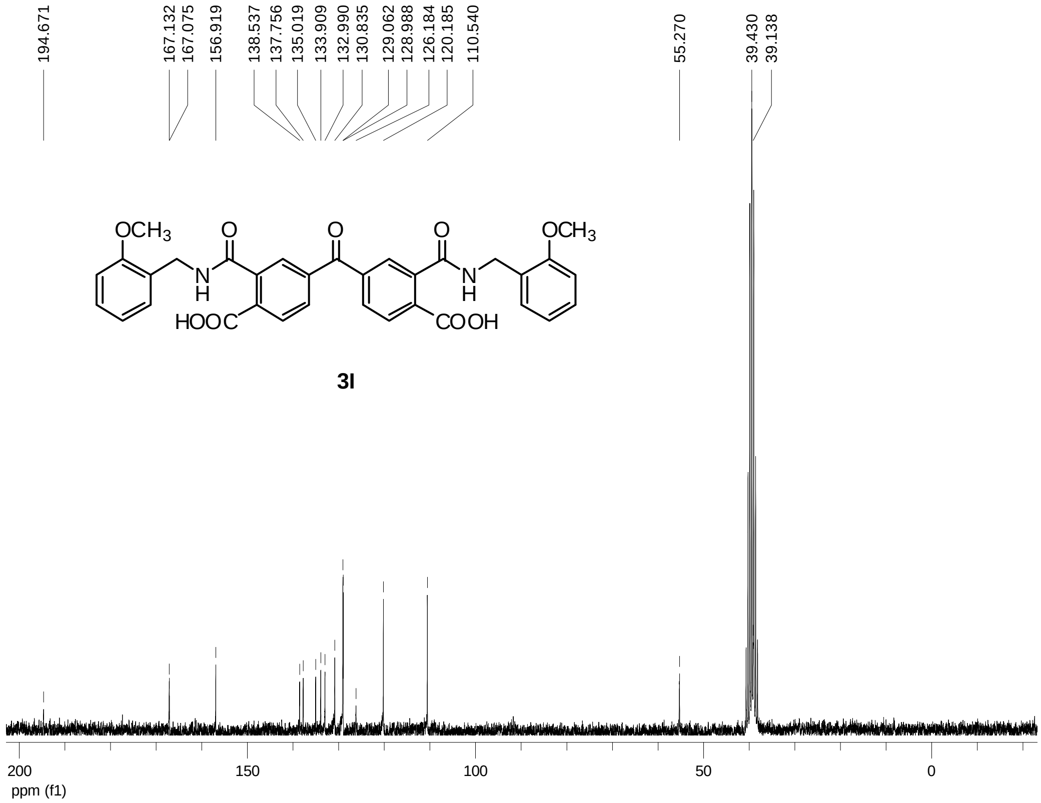
194.671
167.132
167.075
156.919
138.537
137.756
135.019
133.909
132.990
130.835
129.062
128.988
126.184
120.185
110.540
55.270
39.430
39.138
OCH3
O
O
O
OCH3
N
H
N
H
HOOC
COOH
3l
200
150
100
50
0
ppm (f1)

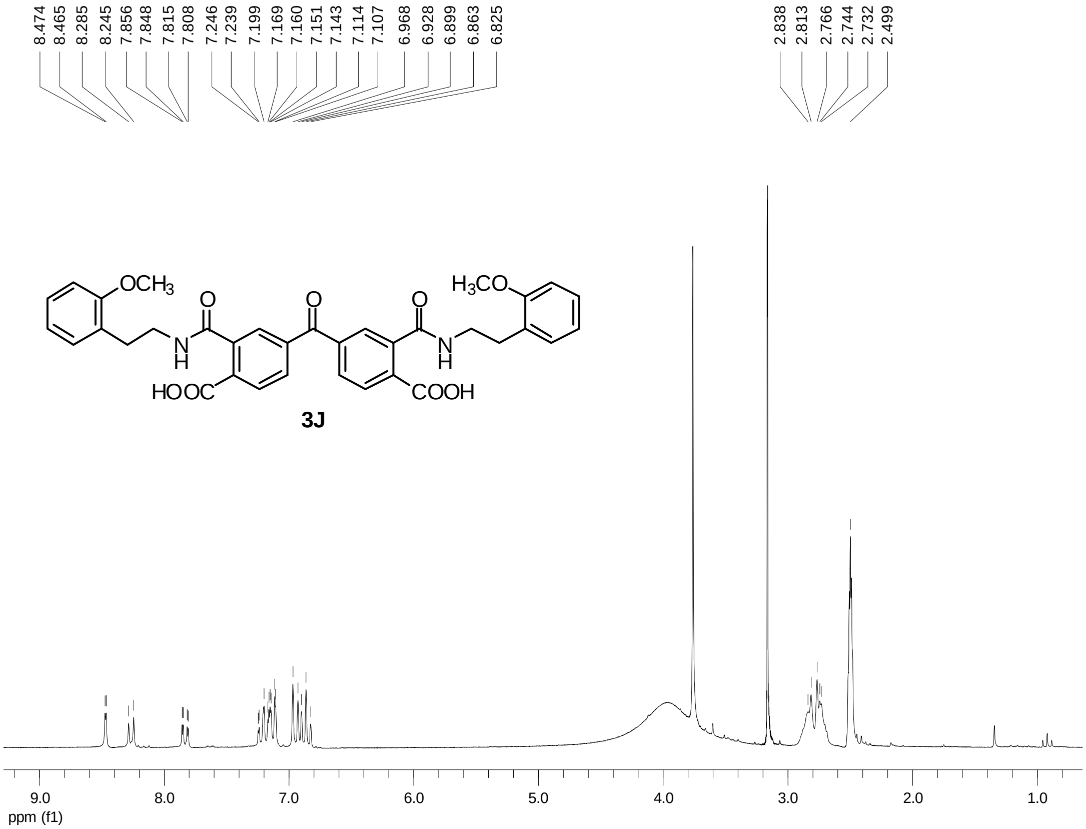
8.474
8.465
8.285
8.245
7.856
7.848
7.815
7.808
7.246
7.239
7.199
7.169
7.160
7.151
7.143
7.114
7.107
6.968
6.928
6.899
6.863
6.825
2.838
2.813
2.766
2.744
2.732
2.499
OCH3
H3CO
O
O
O
N
H
N
H
HOOC
COOH
3J
9.0
8.0
7.0
6.0
5.0
4.0
3.0
2.0
1.0
ppm (f1)

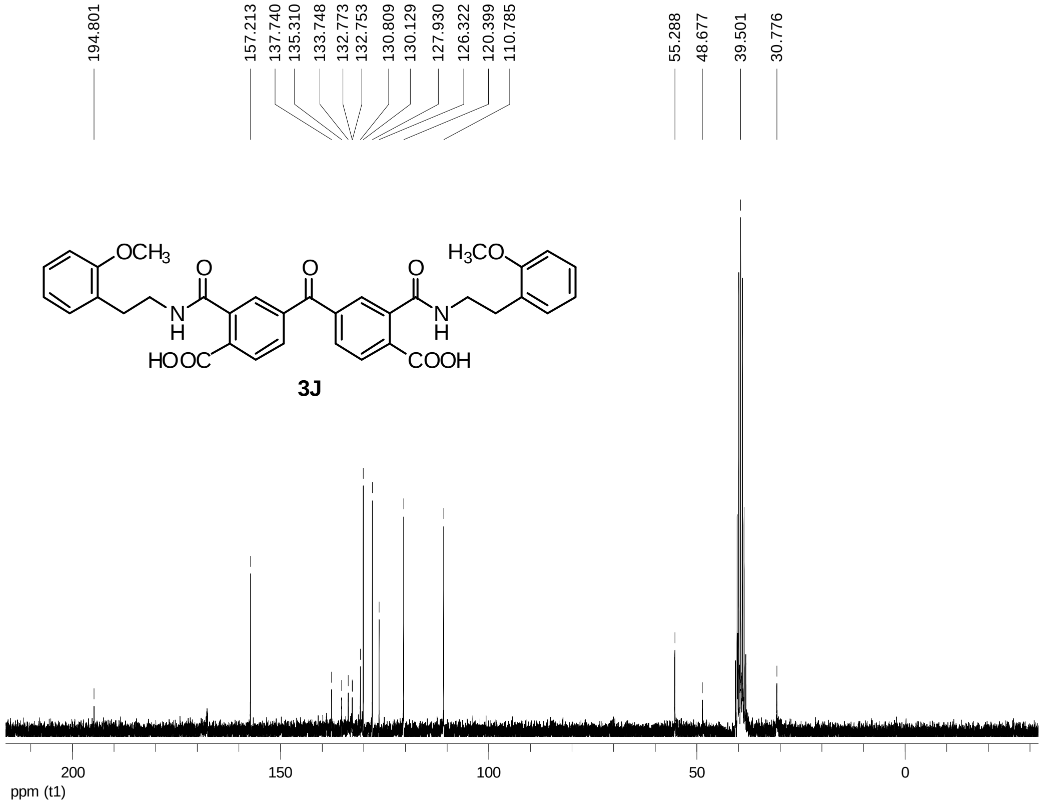
194.801
157.213
137.740
135.310
133.748
132.773
132.753
130.809
130.129
127.930
126.322
120.399
110.785
55.288
48.677
39.501
30.776
OCH3
H3CO
O
O
O
N
H
N
H
HOOC
COOH
3J
200
150
100
50
0
ppm (t1)

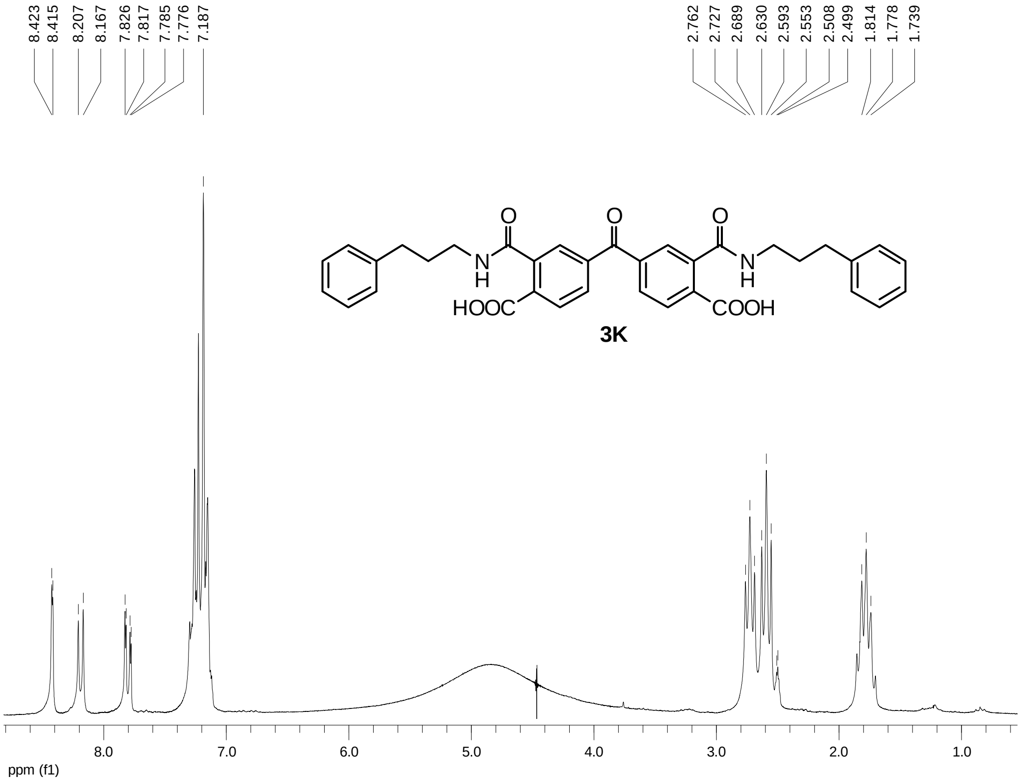
8.423
8.415
8.207
8.167
7.826
7.817
7.785
7.776
7.187
2.762
2.727
2.689
2.630
2.593
2.553
2.508
2.499
1.814
1.778
1.739
O
O
O
N
H
N
H
HOOC
COOH
3K
8.0
7.0
6.0
5.0
4.0
3.0
2.0
1.0
ppm (f1)

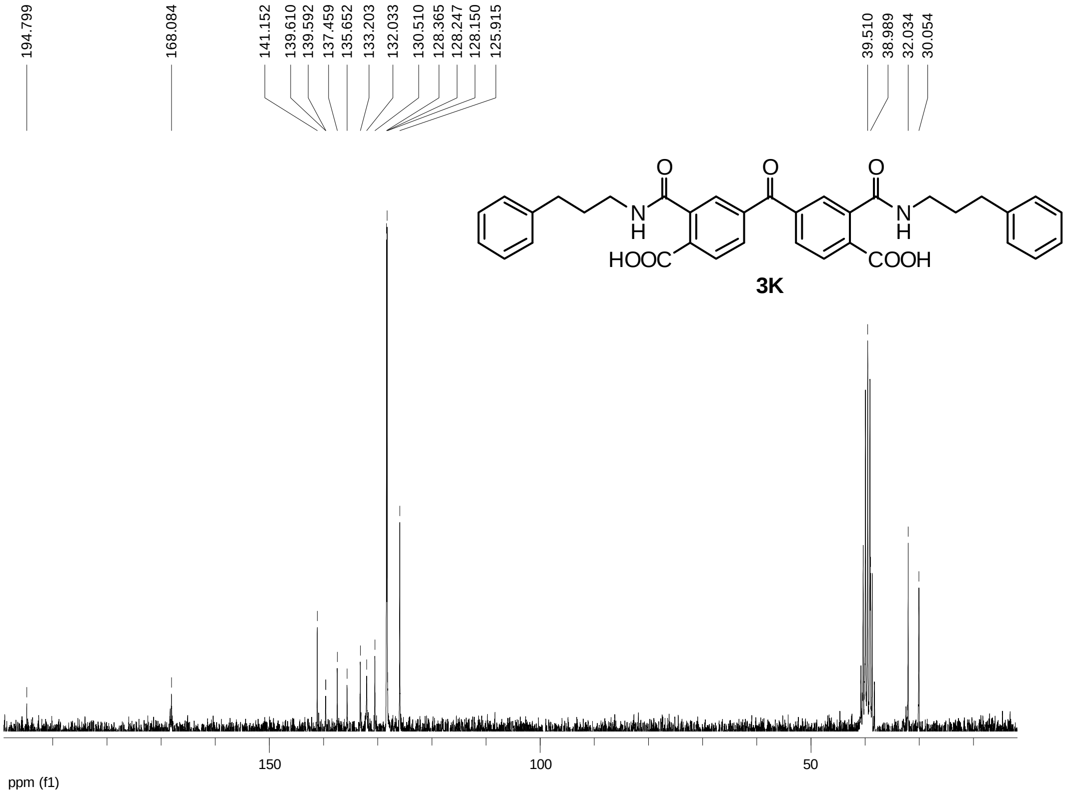
194.799
168.084
141.152
139.610
139.592
137.459
135.652
133.203
132.033
130.510
128.365
128.247
128.150
125.915
39.510
38.989
32.034
30.054
O
O
O
N
H
N
H
HOOC
COOH
3K
150
100
50
ppm (f1)

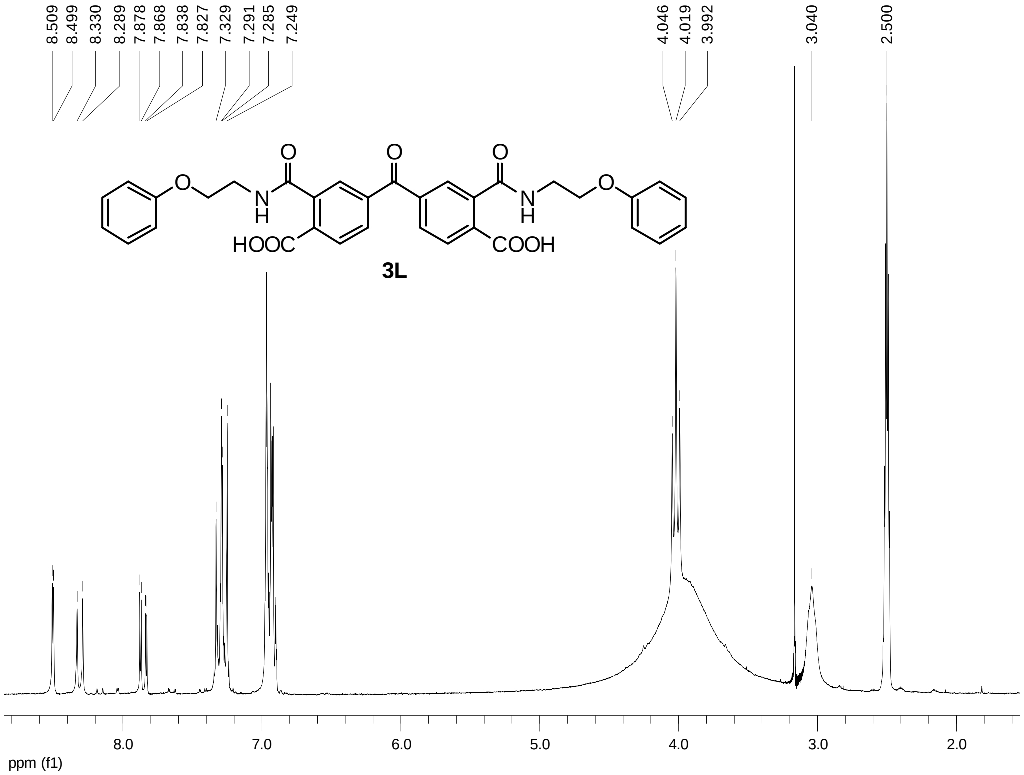
8.509 8.499 8.330 8.289 7.878 7.868 7.838 7.827 7.329 7.291 7.285 7.249
4.046 4.019 3.992
3.040
2.500
HOOC
COOH
3L
8.0 7.0 6.0 5.0 4.0 3.0 2.0
ppm (f1)

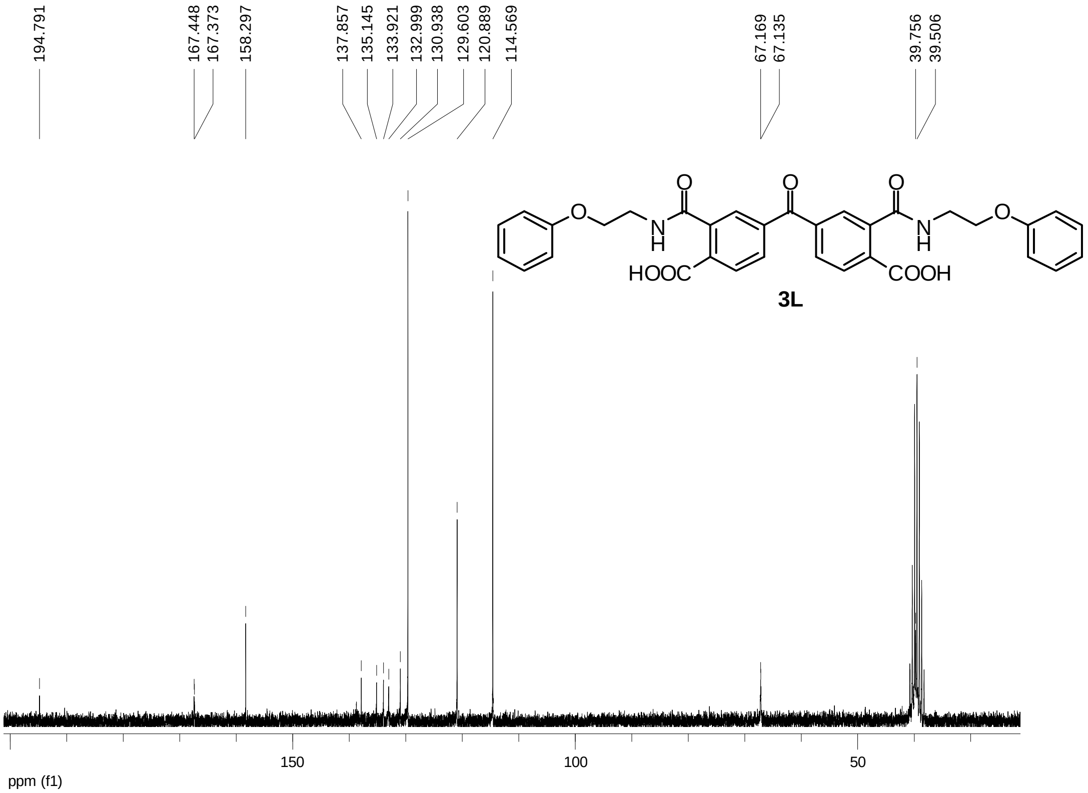
194.791
167.448 167.373 158.297
137.857 135.145 133.921 132.999 130.938 129.603 120.889 114.569
67.169 67.135
39.756 39.506
HOOC
COOH
3L
150 100 50
ppm (f1)

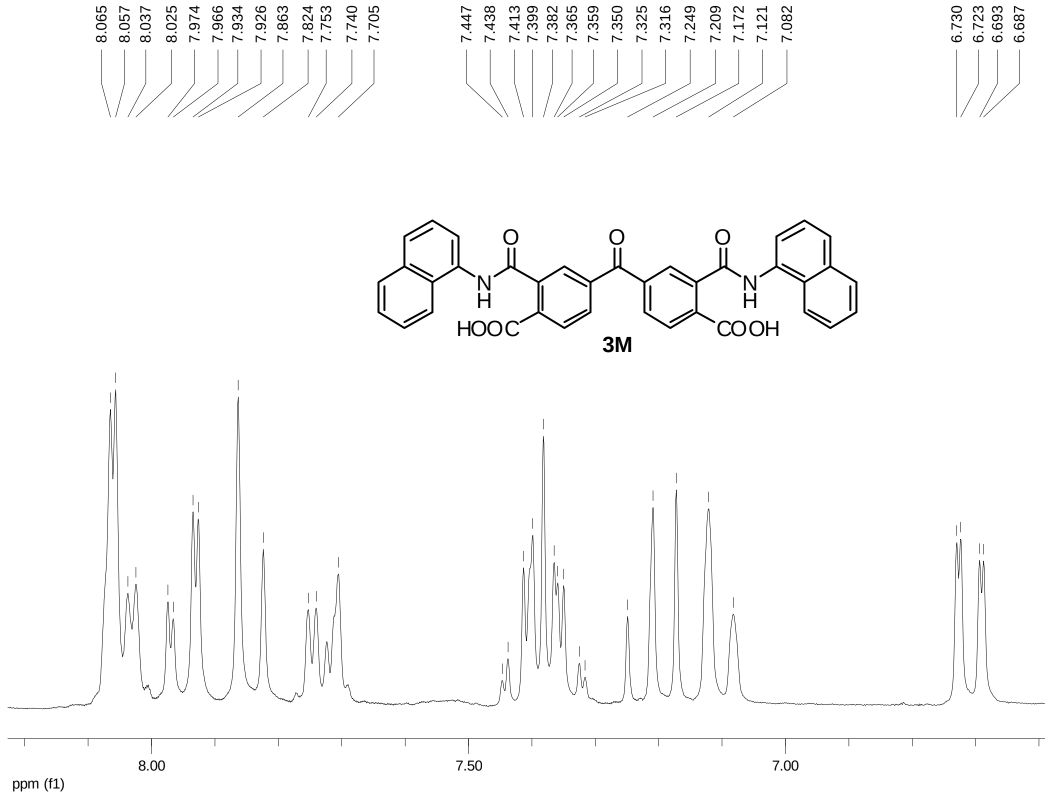
8.065
8.057
8.037
8.025
7.974
7.966
7.934
7.926
7.863
7.824
7.753
7.740
7.705
7.447
7.438
7.413
7.399
7.382
7.365
7.359
7.350
7.325
7.316
7.249
7.209
7.172
7.121
7.082
6.730
6.723
6.693
6.687
HOOC
COOH
3M
8.00
7.50
7.00
ppm (f1)

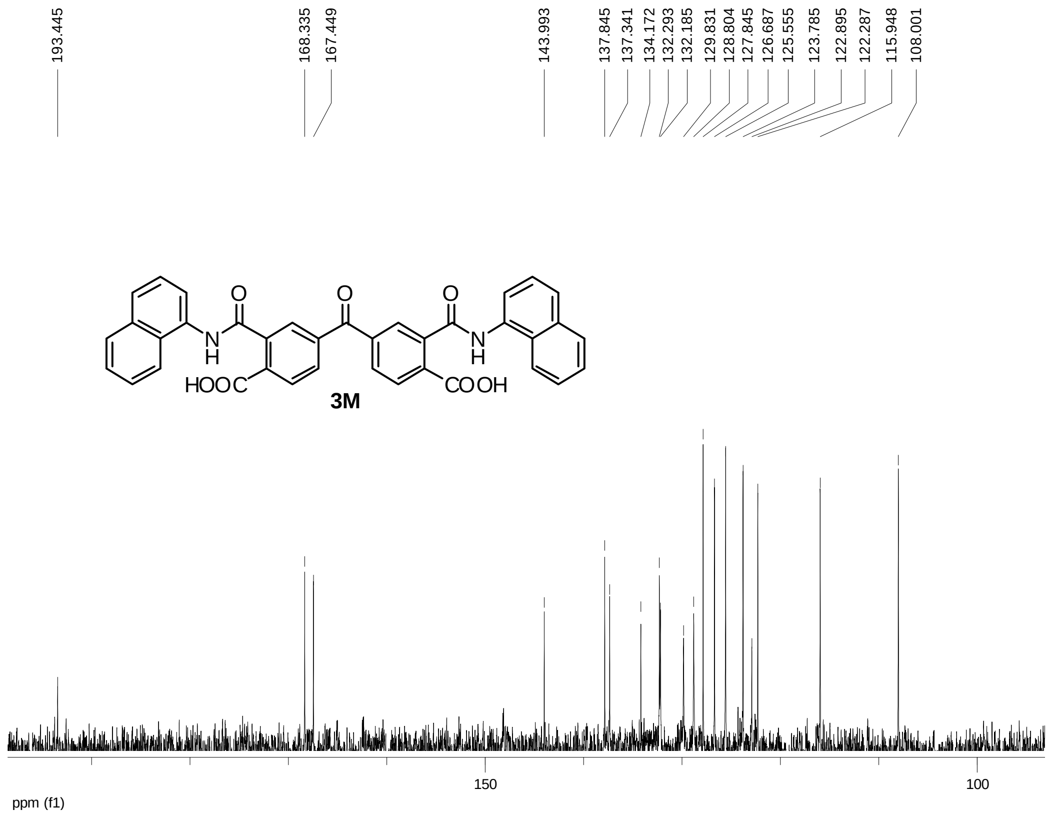
193.445
168.335
167.449
143.993
137.845
137.341
134.172
132.293
132.185
129.831
128.804
127.845
126.687
125.555
123.785
122.895
122.287
115.948
108.001
HOOC
COOH
3M
150
100
ppm (f1)

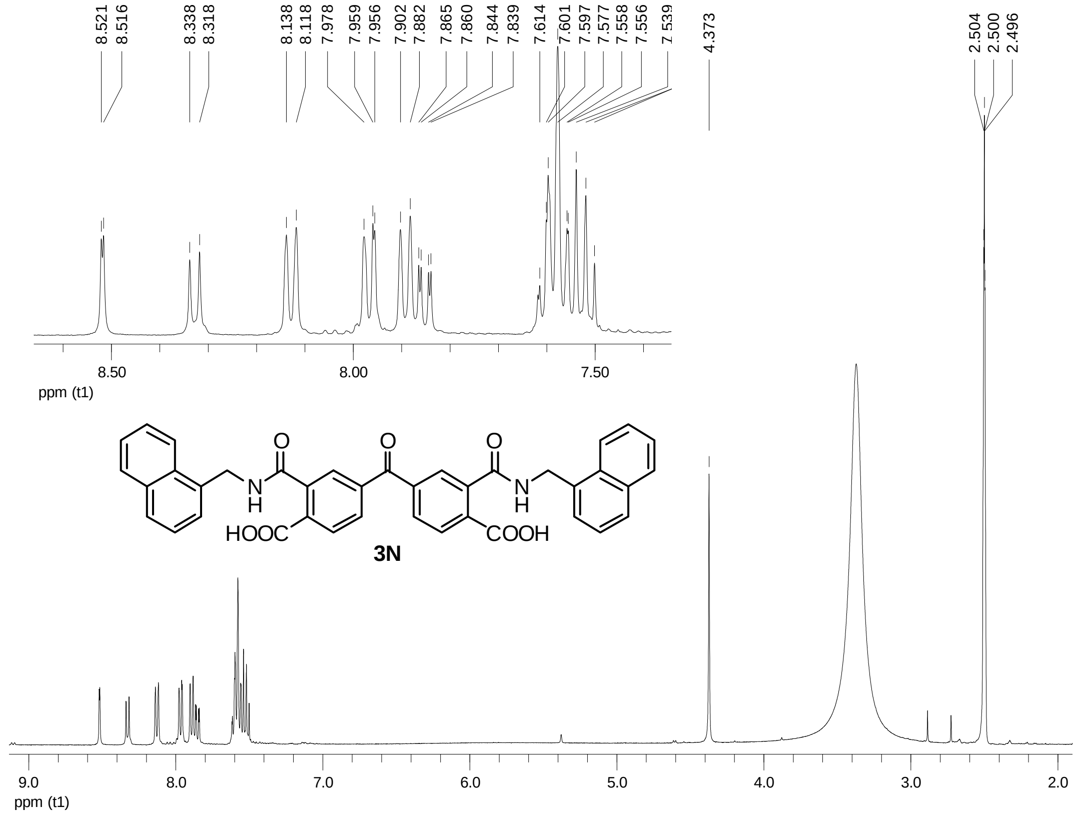
8.521 8.516 8.338 8.318 8.138 8.118 7.978 7.959 7.956 7.902 7.882 7.865 7.860 7.844 7.839 7.614 7.601 7.597 7.577 7.558 7.556 7.539 4.373 2.504 2.500 2.496
8.50 8.00 7.50
ppm (t1)
O O O
N H N H
HOOC COOH
3N
9.0 8.0 7.0 6.0 5.0 4.0 3.0 2.0
ppm (t1)

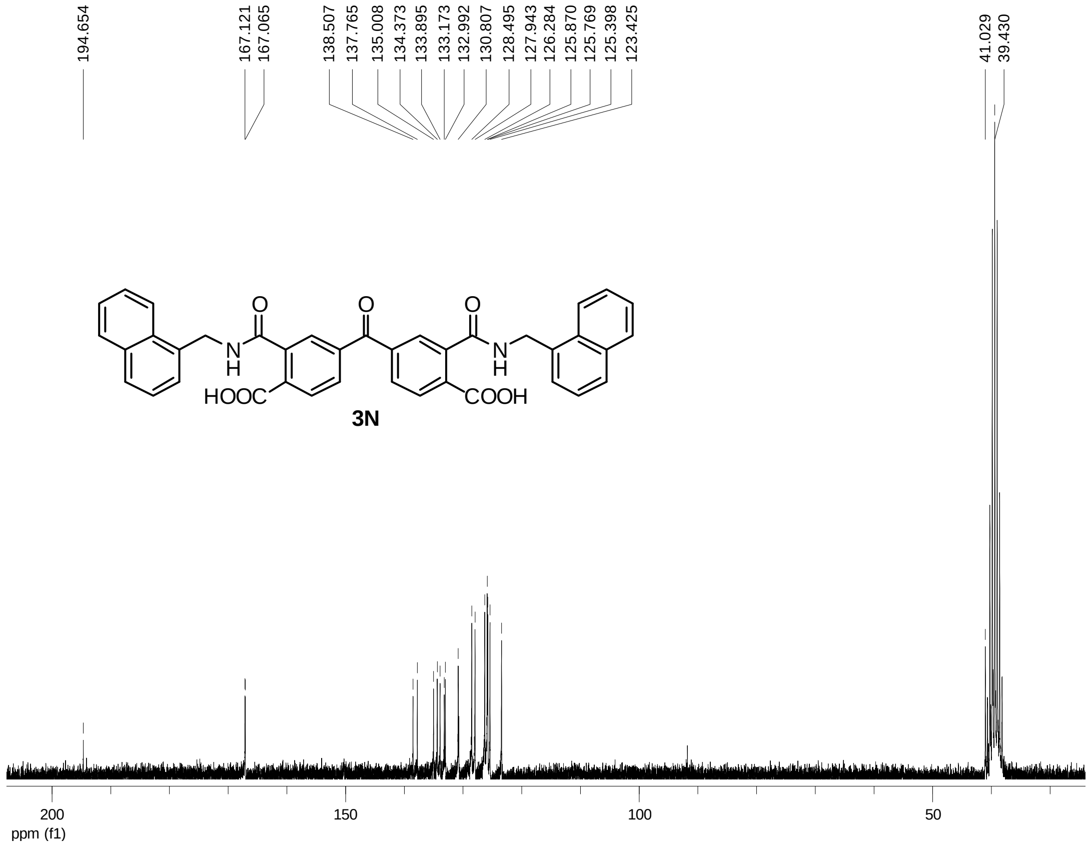
194.654 167.121 167.065 138.507 137.765 135.008 134.373 133.895 133.173 132.992 130.807 128.495 127.943 126.284 125.870 125.769 125.398 123.425 41.029 39.430
O O O
N H N H
HOOC COOH
3N
200 150 100 50
ppm (f1)

II. Selected NMR spectra of compounds 6

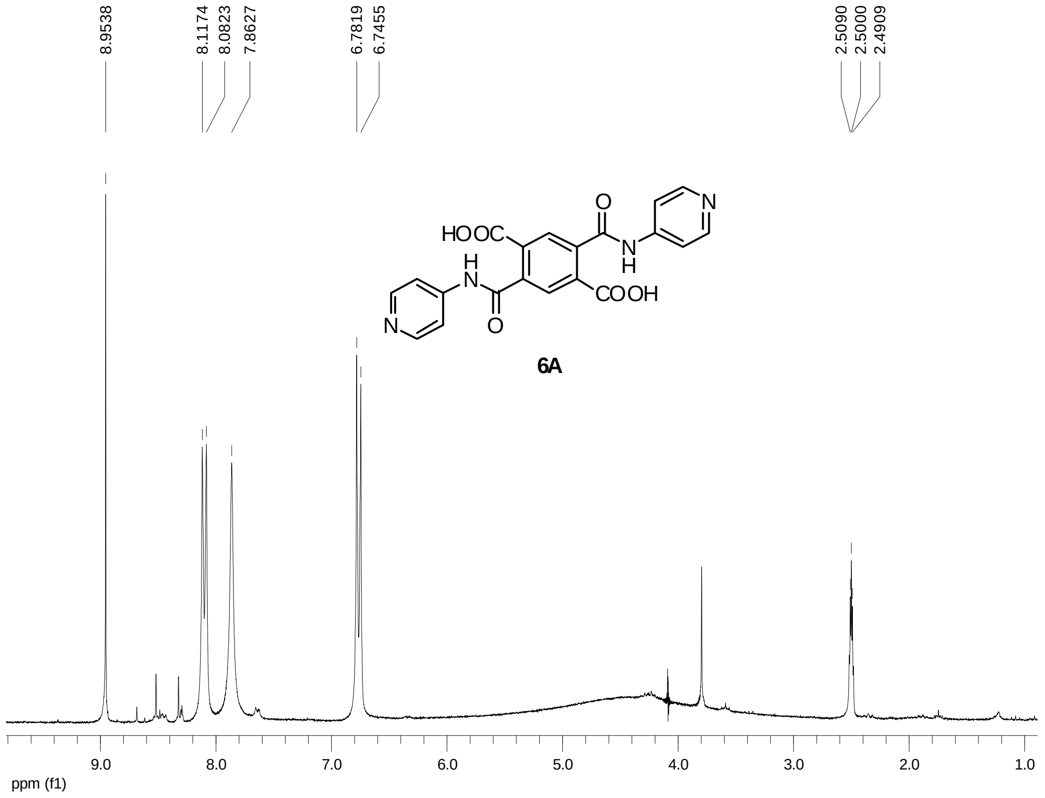

With two drops of D_2O added

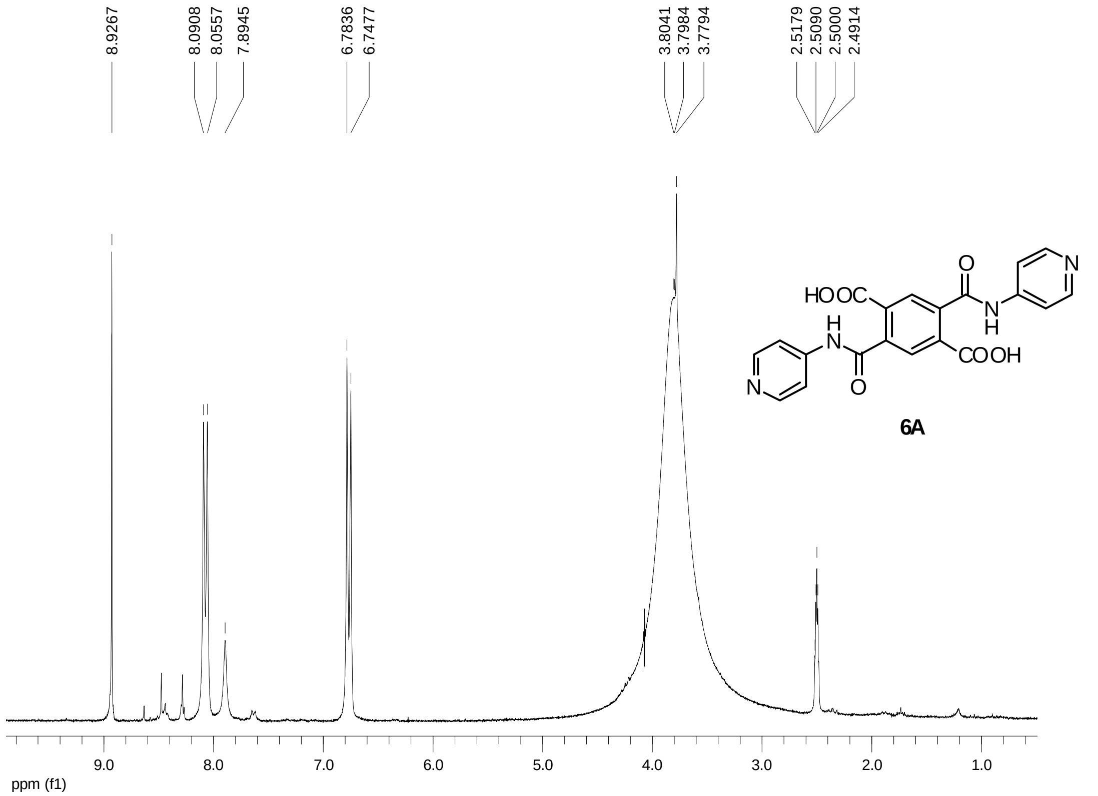

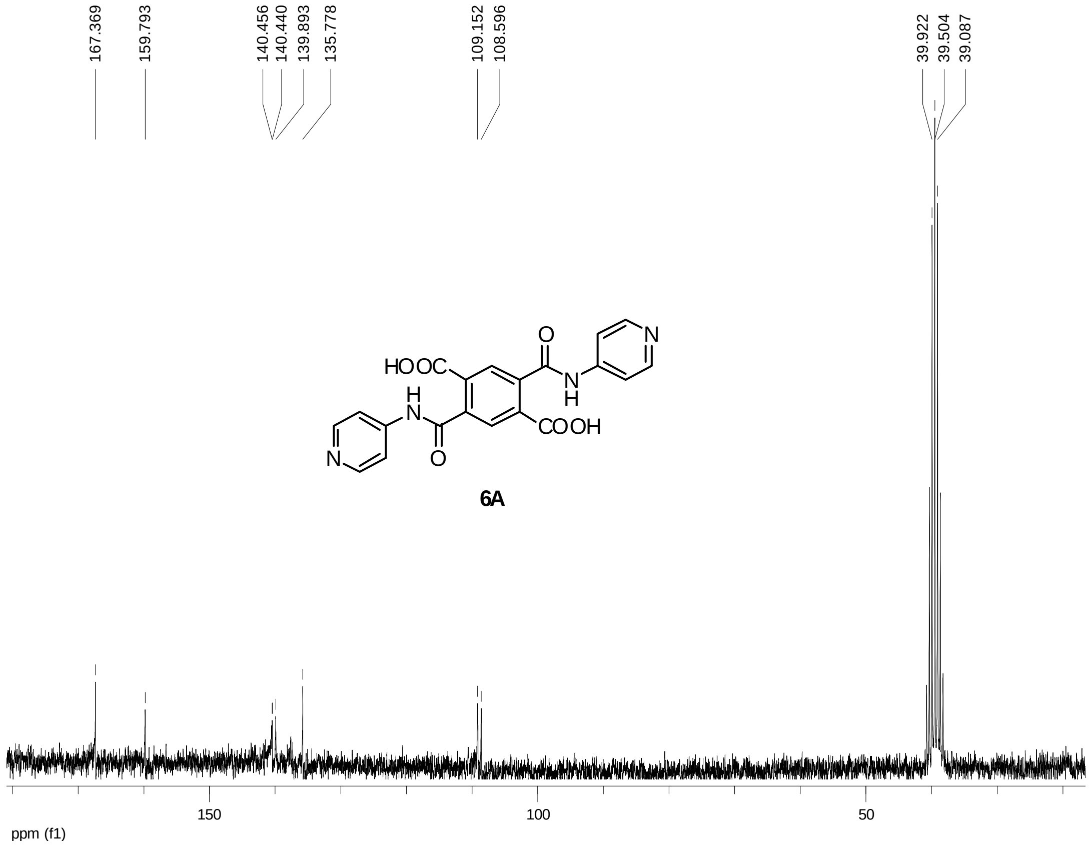
167.369
159.793
140.456
140.440
139.893
135.778
109.152
108.596
39.922
39.504
39.087
HOOC
COOH
N
H
O
6A
150
100
50
ppm (f1)

Measured in D_2O/DCl

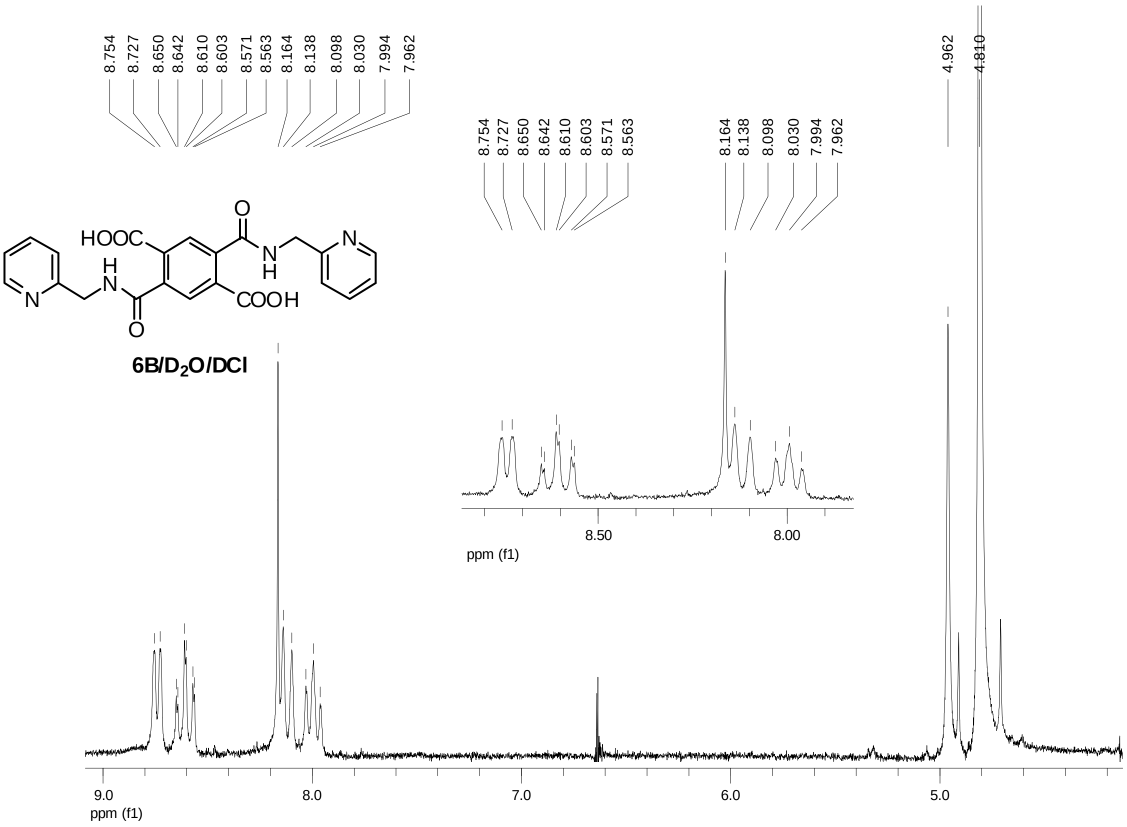

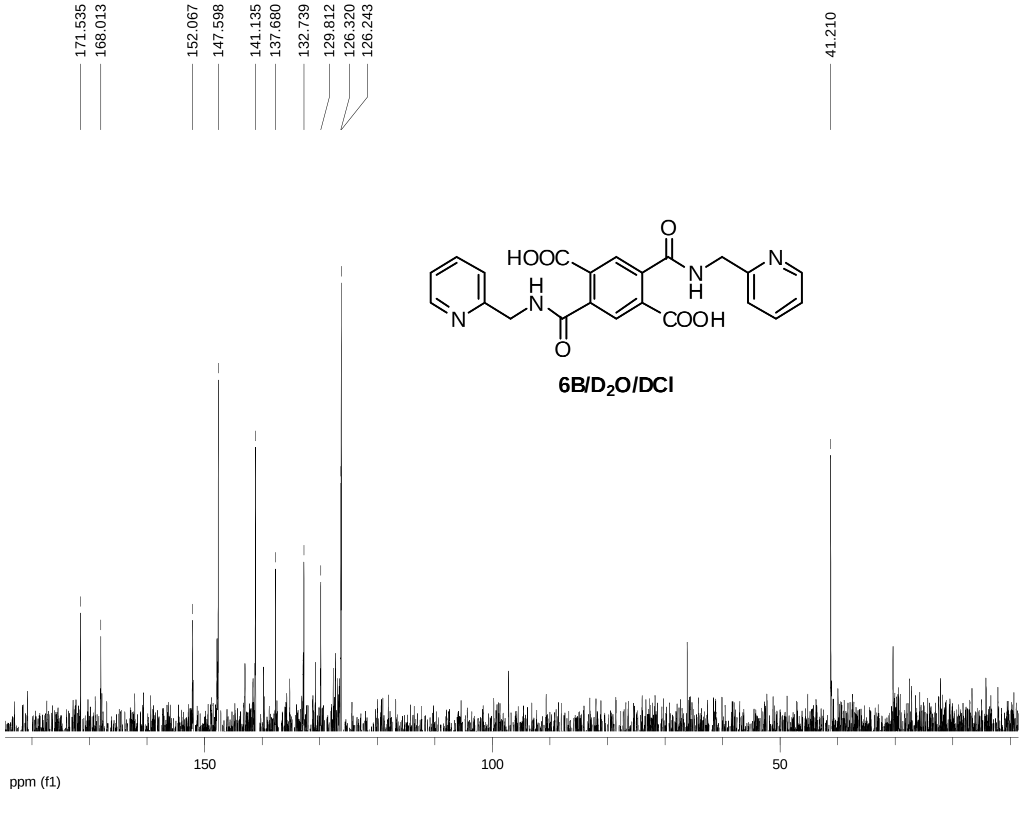

Measured in D_2O/DCl

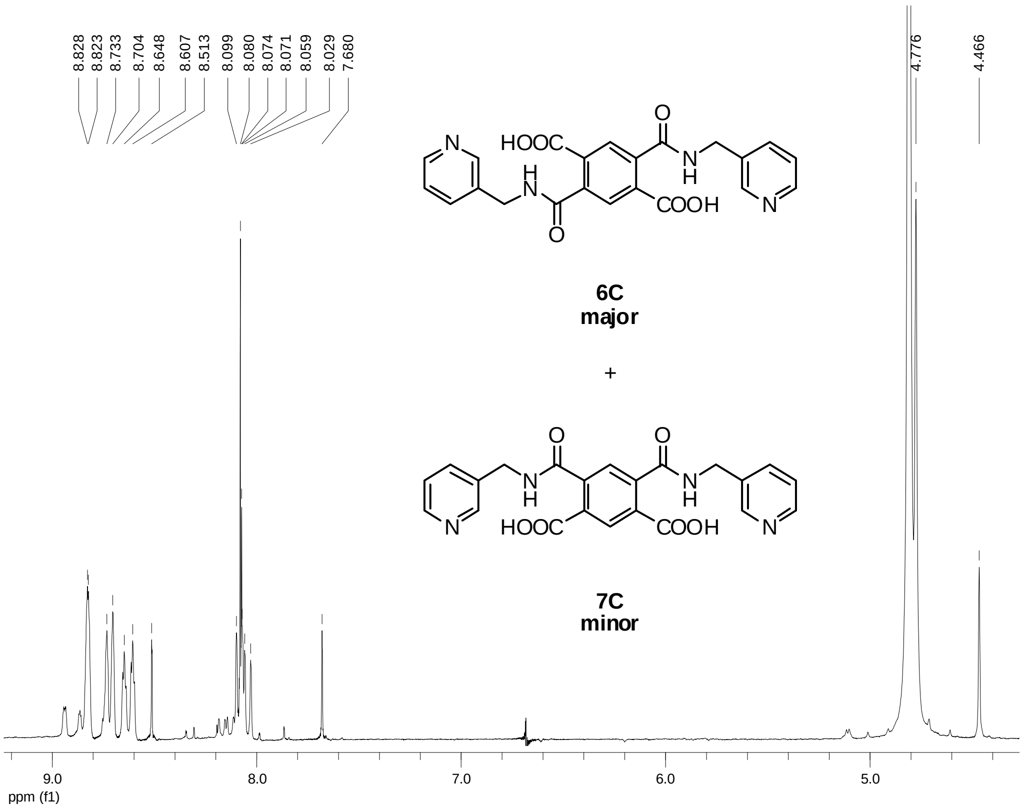

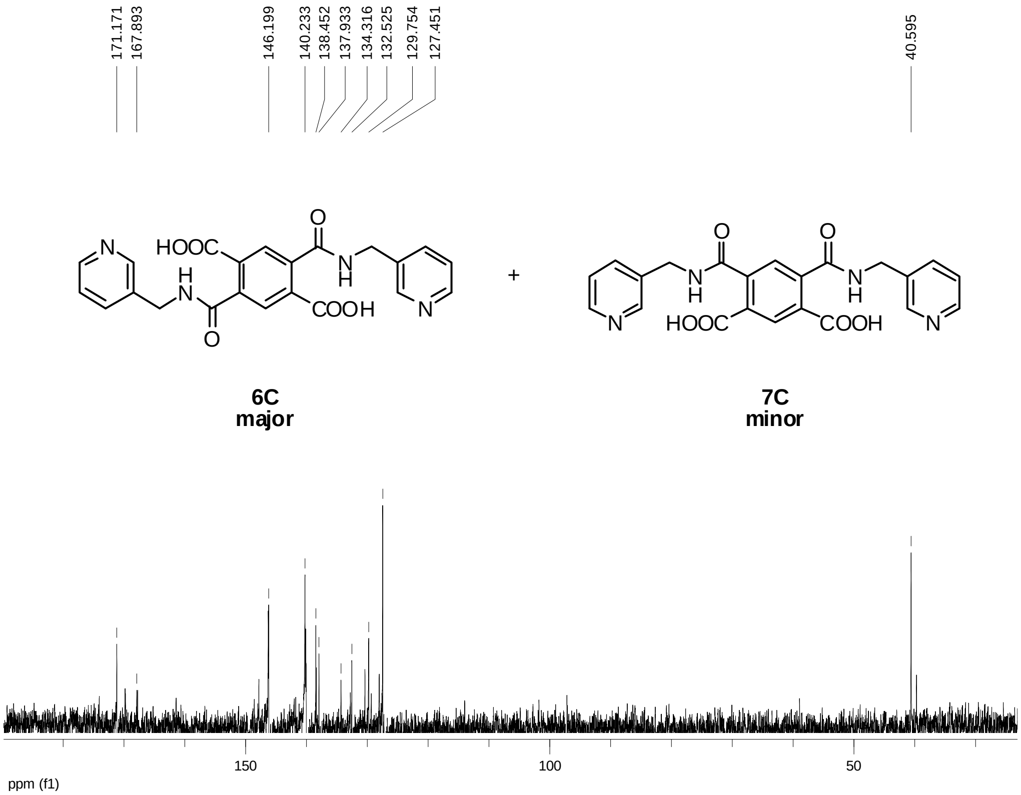

Measured in D_2O/DCl

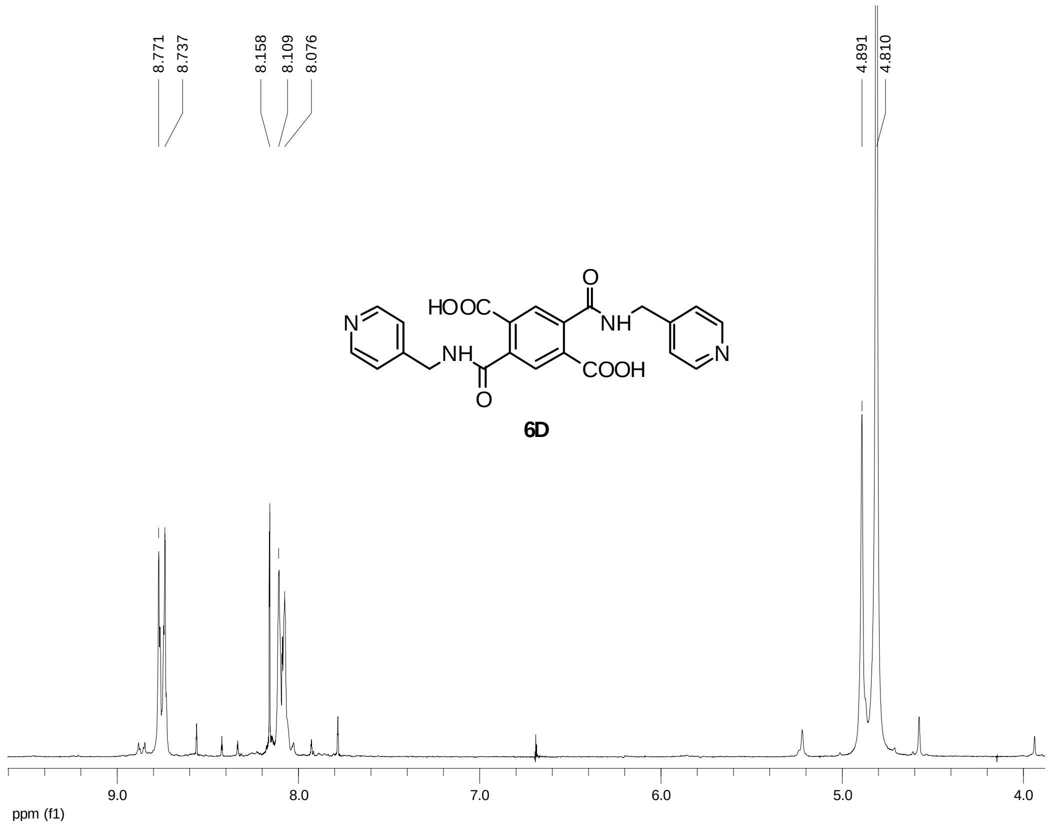

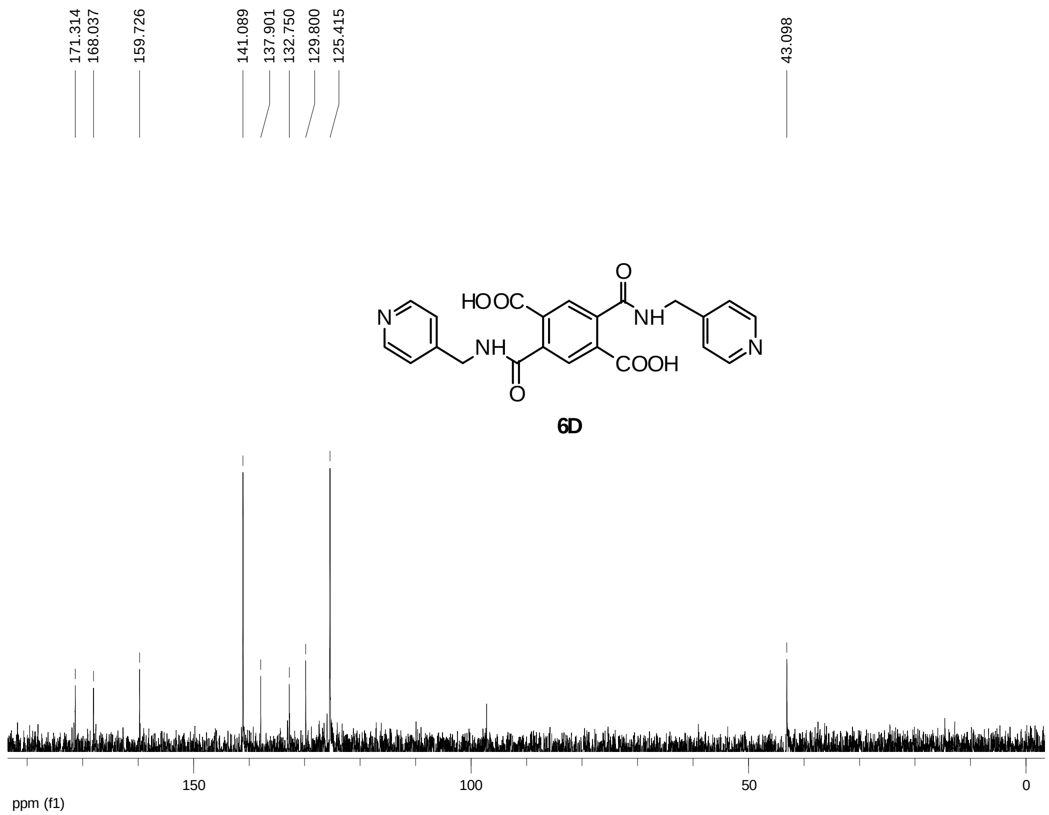

Reaction in THF

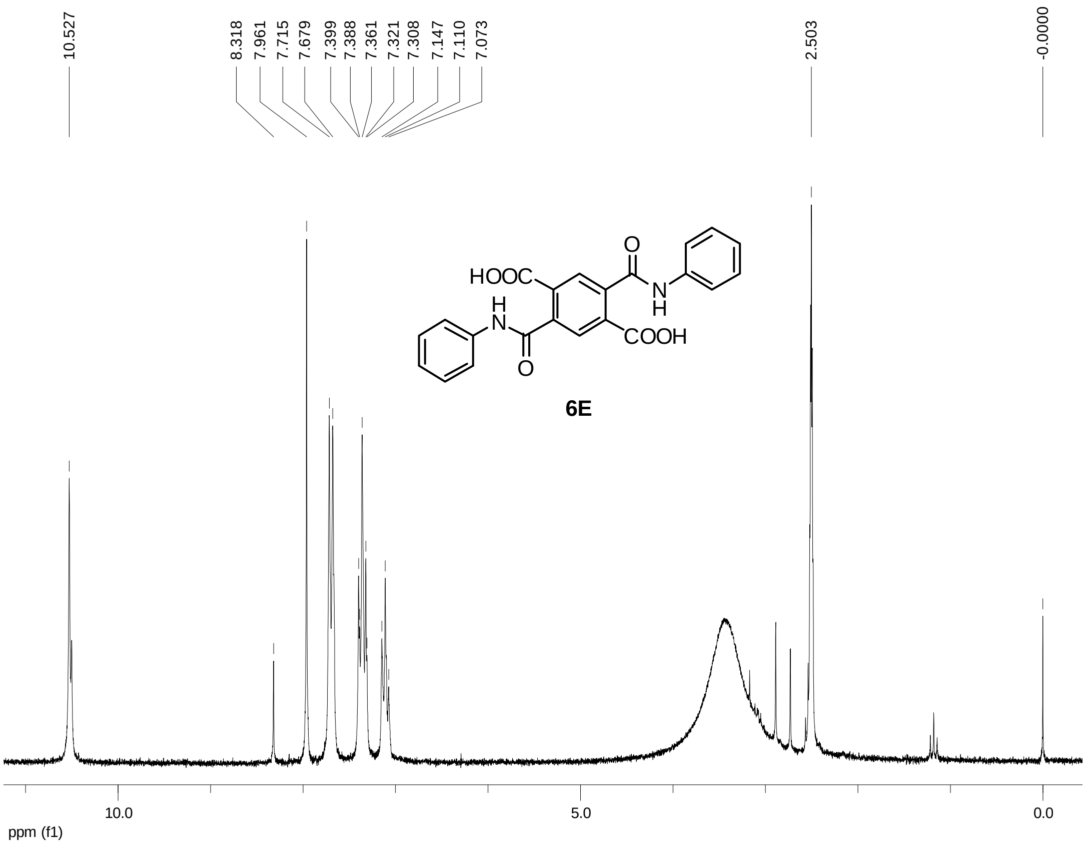

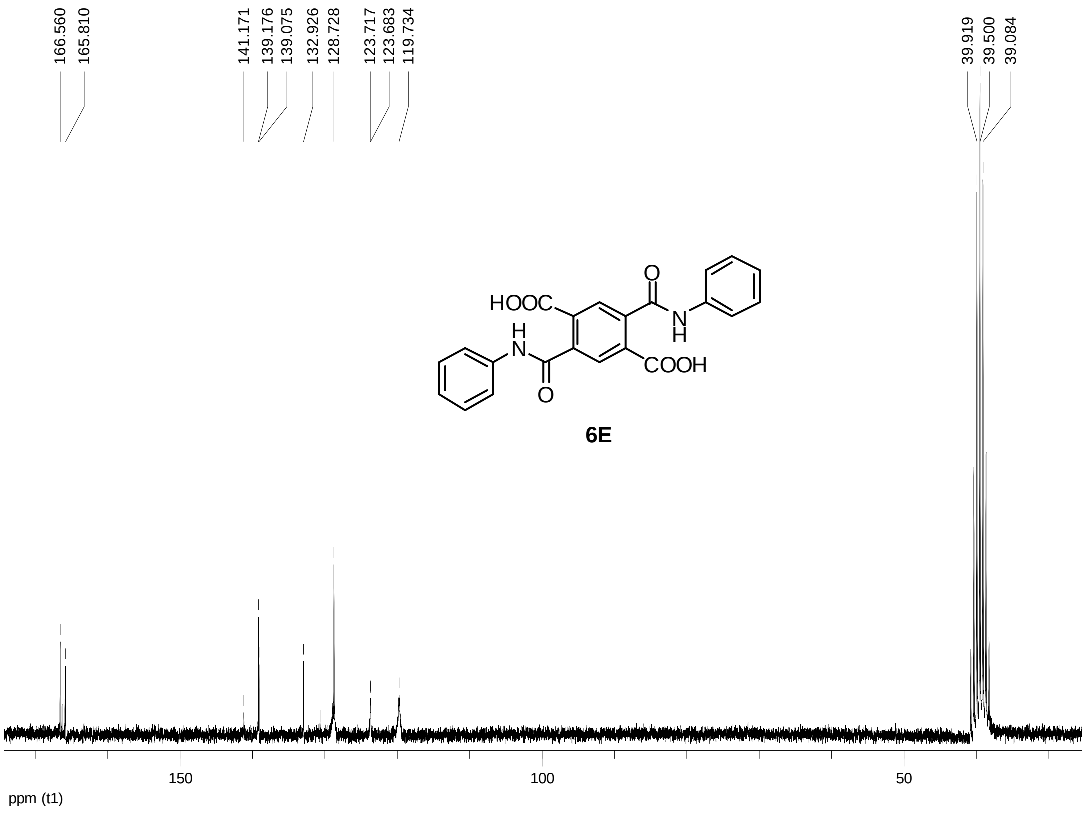

Reaction in Ethanol

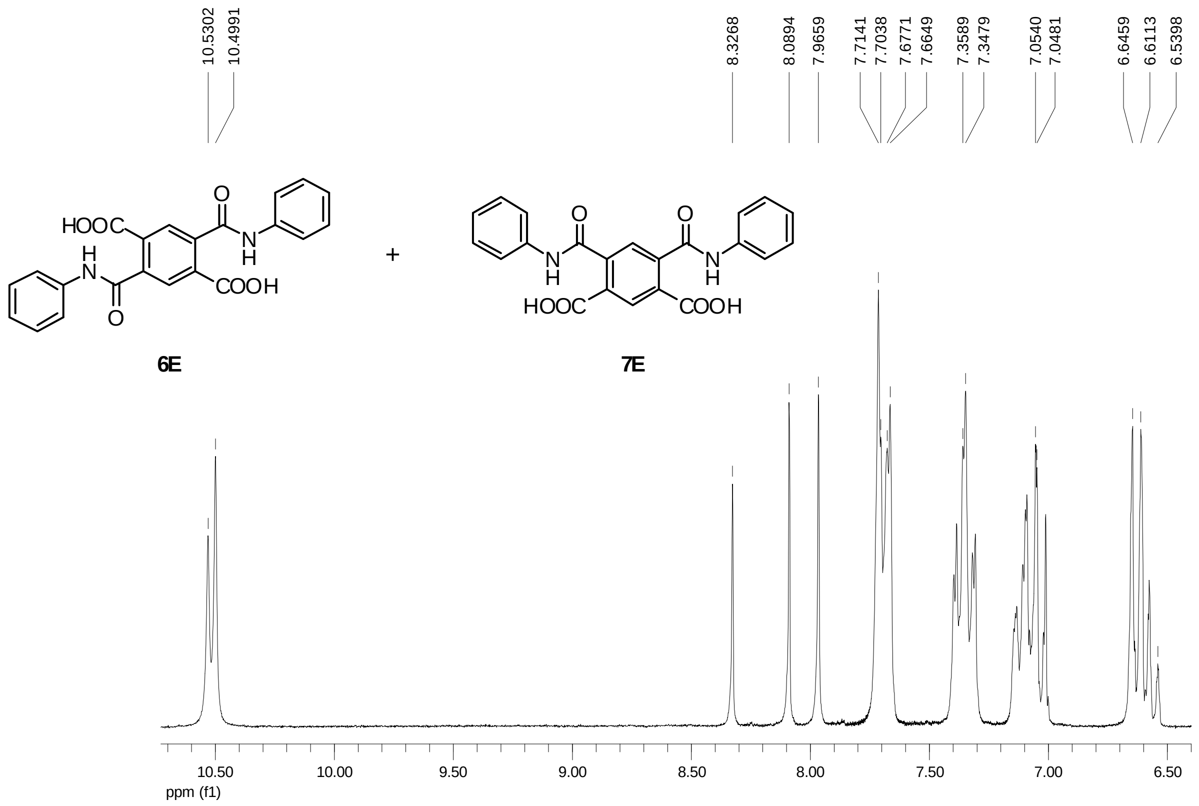

Reaction in THF

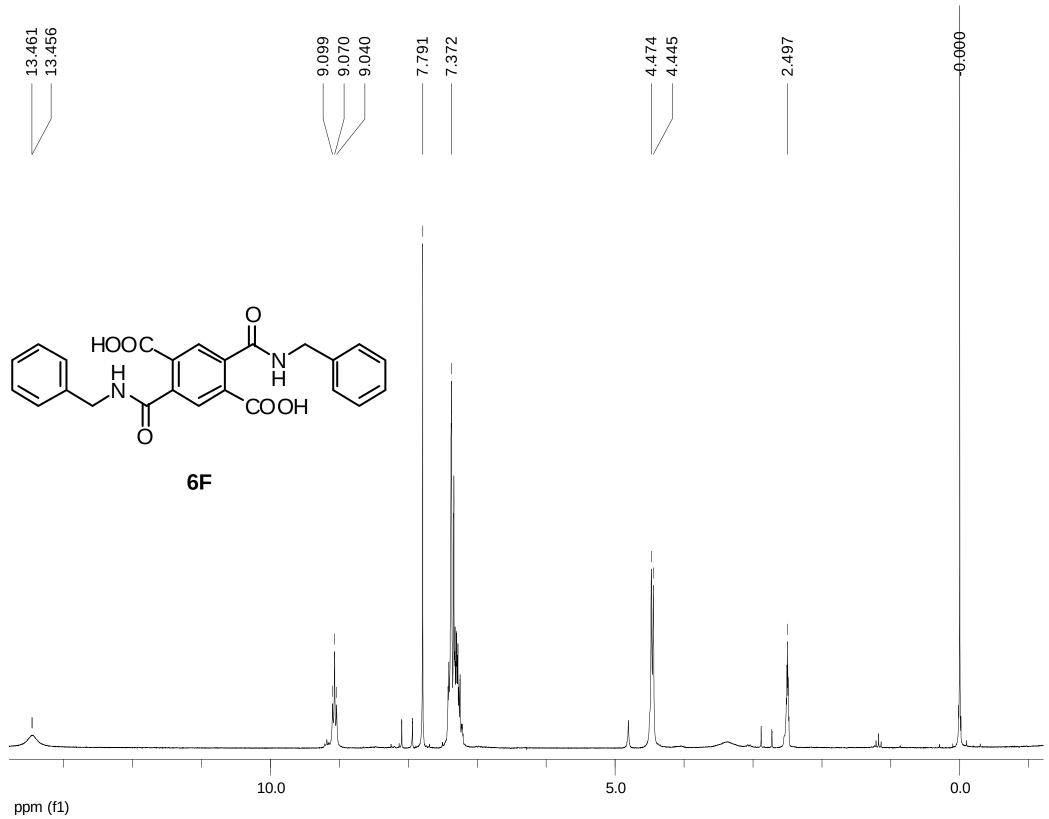

Reaction in Ethanol

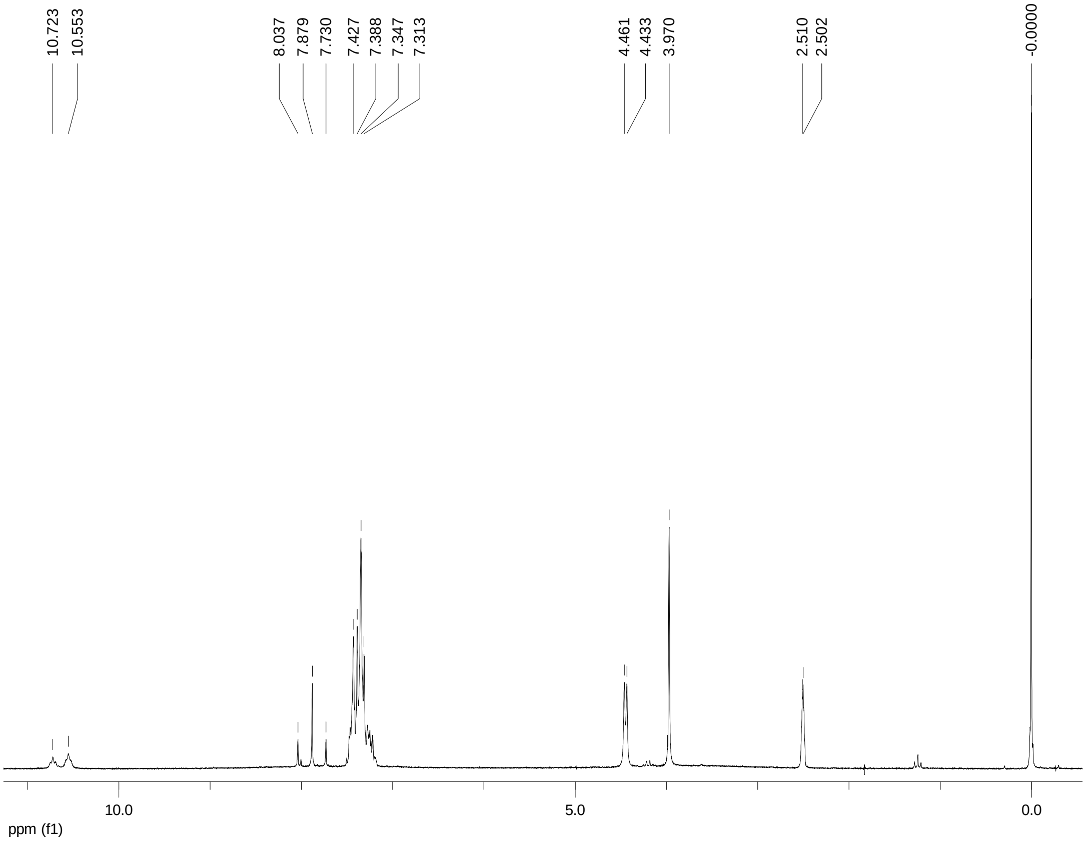

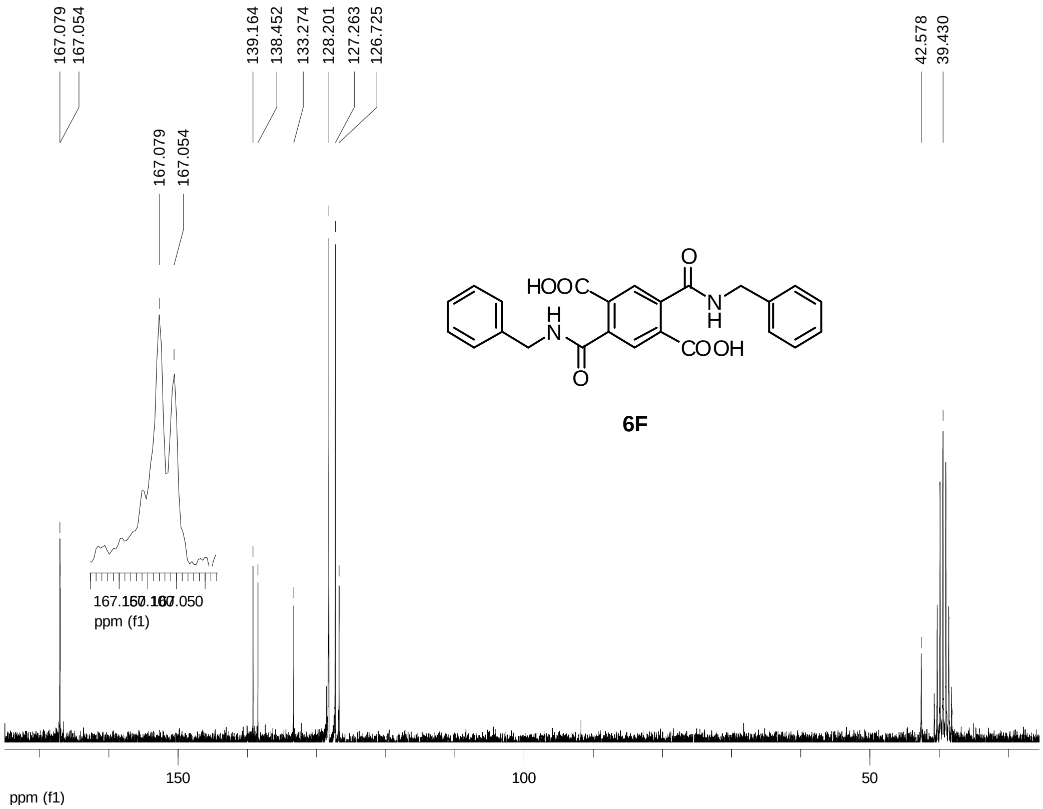
167.079
167.054
139.164
138.452
133.274
128.201
127.263
126.725
42.578
39.430
167.079
167.054
167.150
167.100
167.050
ppm (f1)
HOOC
H
N
O
N
H
COOH
6F
150
100
50
ppm (f1)

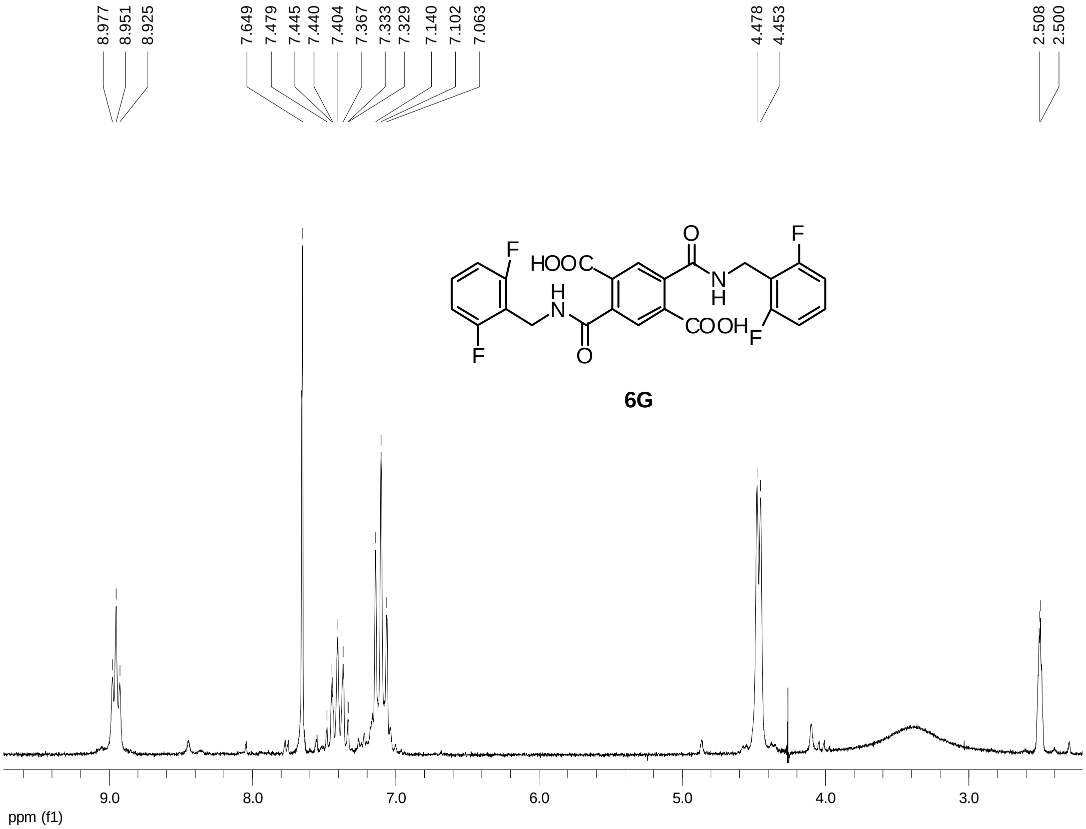
8.977
8.951
8.925
7.649
7.479
7.445
7.440
7.404
7.367
7.333
7.329
7.140
7.102
7.063
4.478
4.453
2.508
2.500
6G
9.0
8.0
7.0
6.0
5.0
4.0
3.0
ppm (f1)

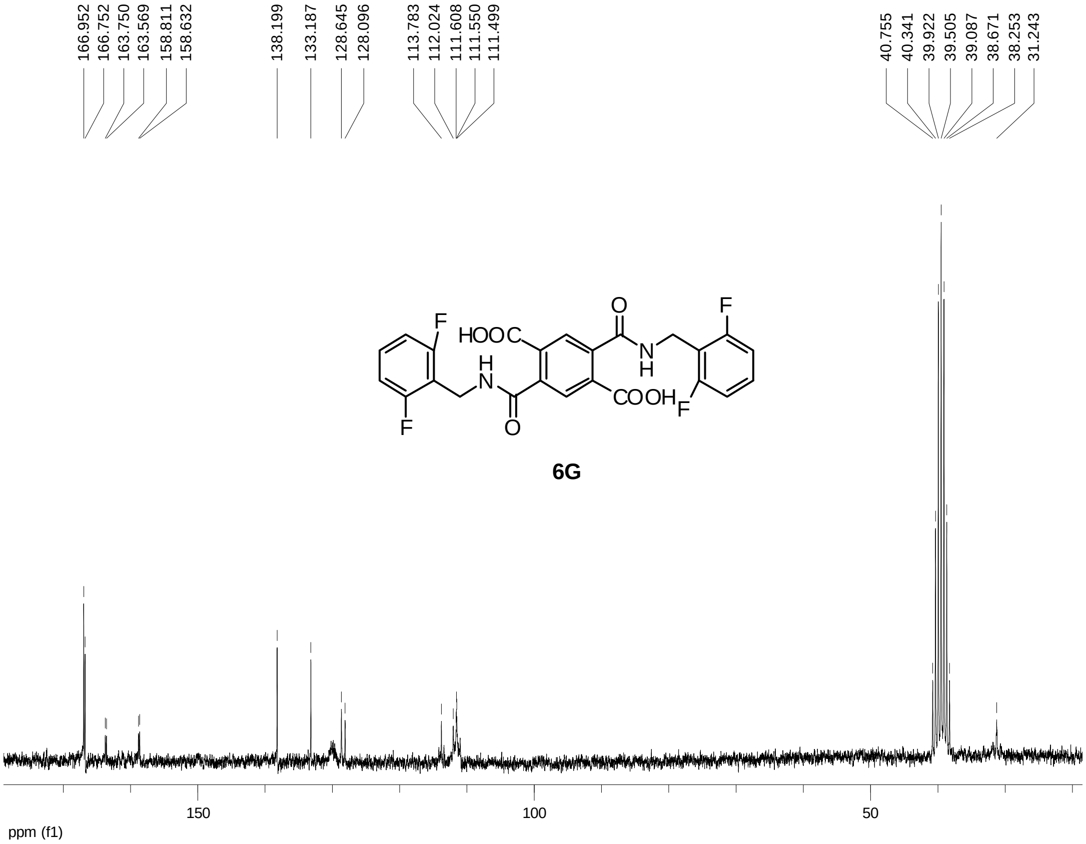
166.952
166.752
163.750
163.569
158.811
158.632
138.199
133.187
128.645
128.096
113.783
112.024
111.608
111.550
111.499
40.755
40.341
39.922
39.505
39.087
38.671
38.253
31.243
6G
150
100
50
ppm (f1)

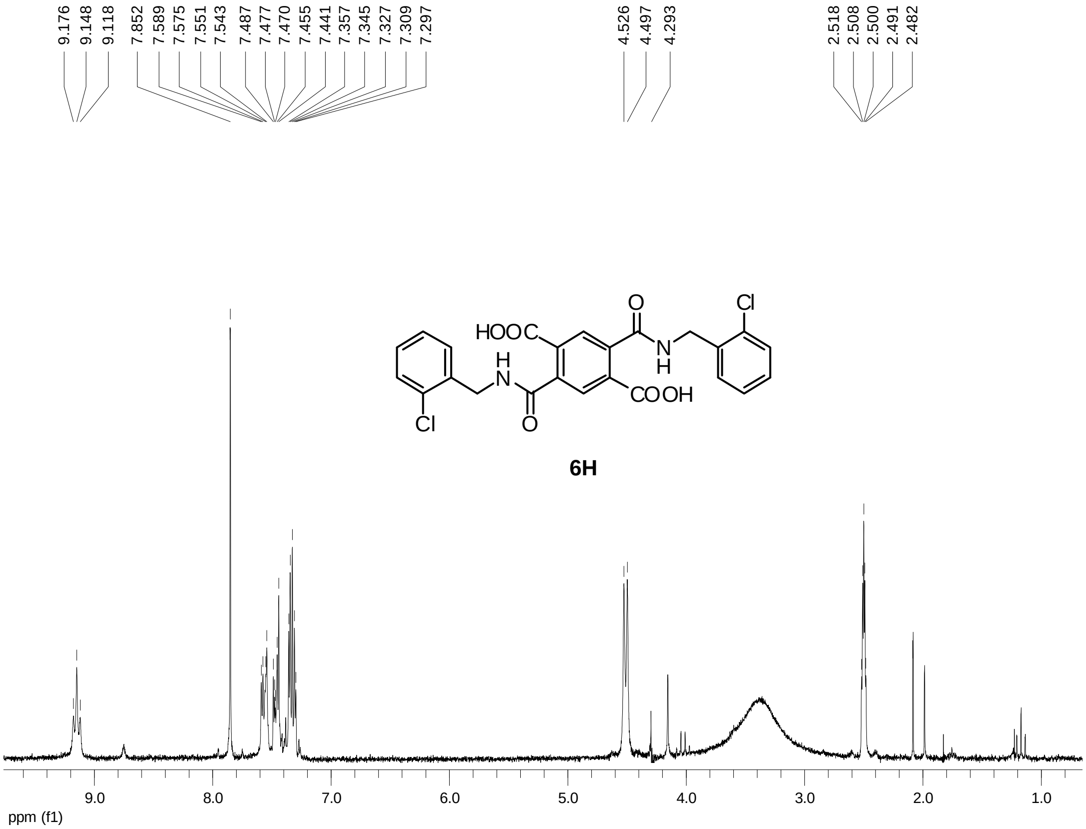
9.176
9.148
9.118
7.852
7.589
7.575
7.551
7.543
7.487
7.477
7.470
7.455
7.441
7.357
7.345
7.327
7.309
7.297
4.526
4.497
4.293
2.518
2.508
2.500
2.491
2.482
HOOC
COOH
Cl
Cl
O
O
H
N
N
H
6H
9.0
8.0
7.0
6.0
5.0
4.0
3.0
2.0
1.0
ppm (f1)

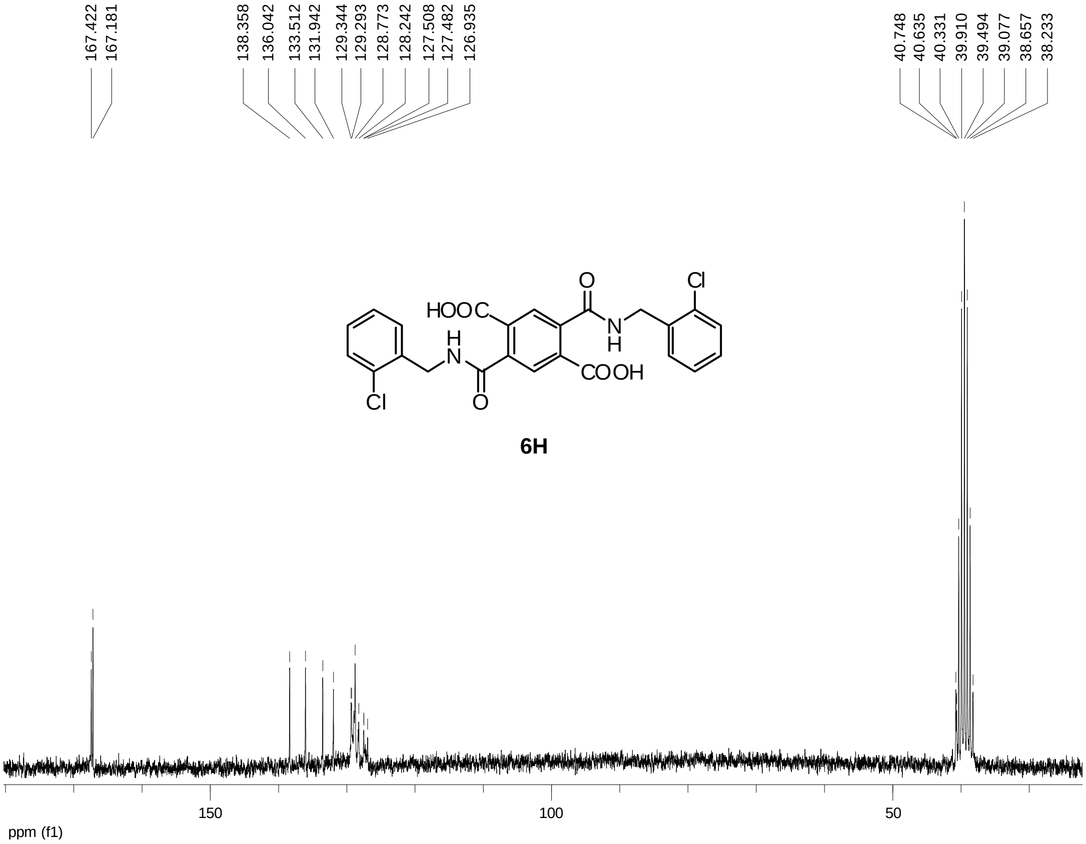
167.422
167.181
138.358
136.042
133.512
131.942
129.344
129.293
128.773
128.242
127.508
127.482
126.935
40.748
40.635
40.331
39.910
39.494
39.077
38.657
38.233
HOOC
COOH
Cl
Cl
O
O
H
N
N
H
6H
150
100
50
ppm (f1)

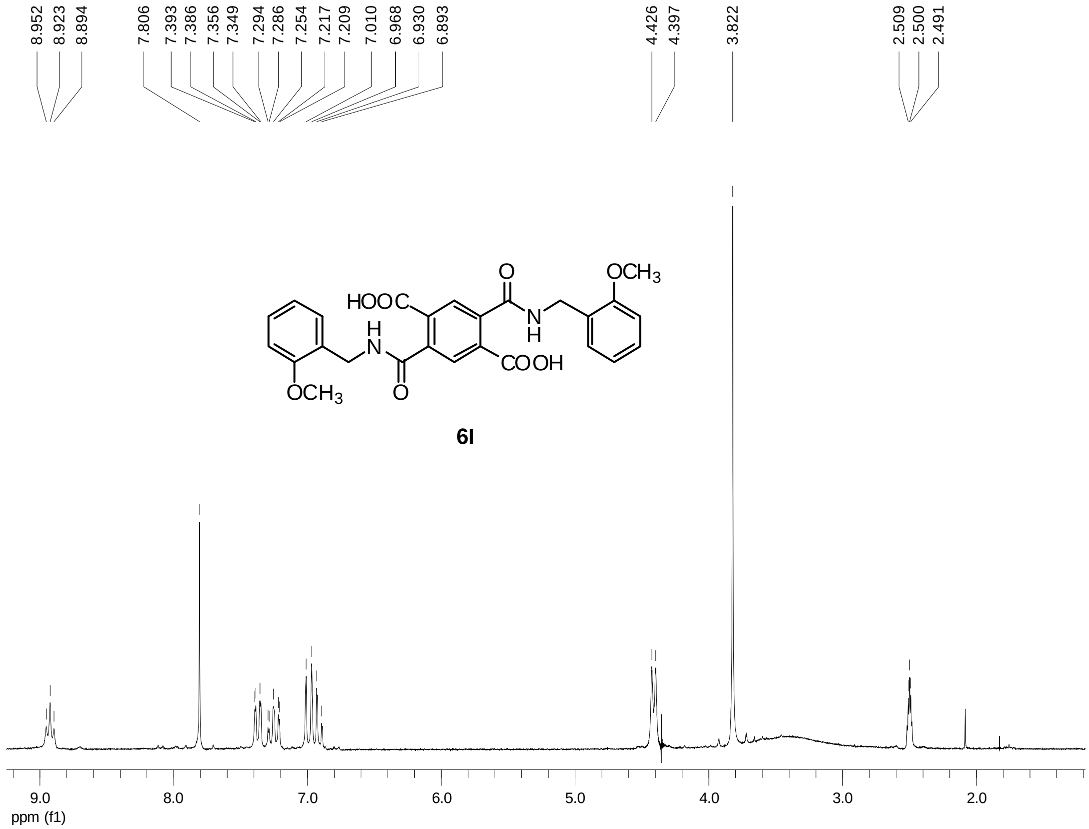
8.952
8.923
8.894
7.806
7.393
7.386
7.356
7.349
7.294
7.286
7.254
7.217
7.209
7.010
6.968
6.930
6.893
4.426
4.397
3.822
2.509
2.500
2.491
HOOC
COOH
OCH3
6l
9.0
8.0
7.0
6.0
5.0
4.0
3.0
2.0
ppm (f1)

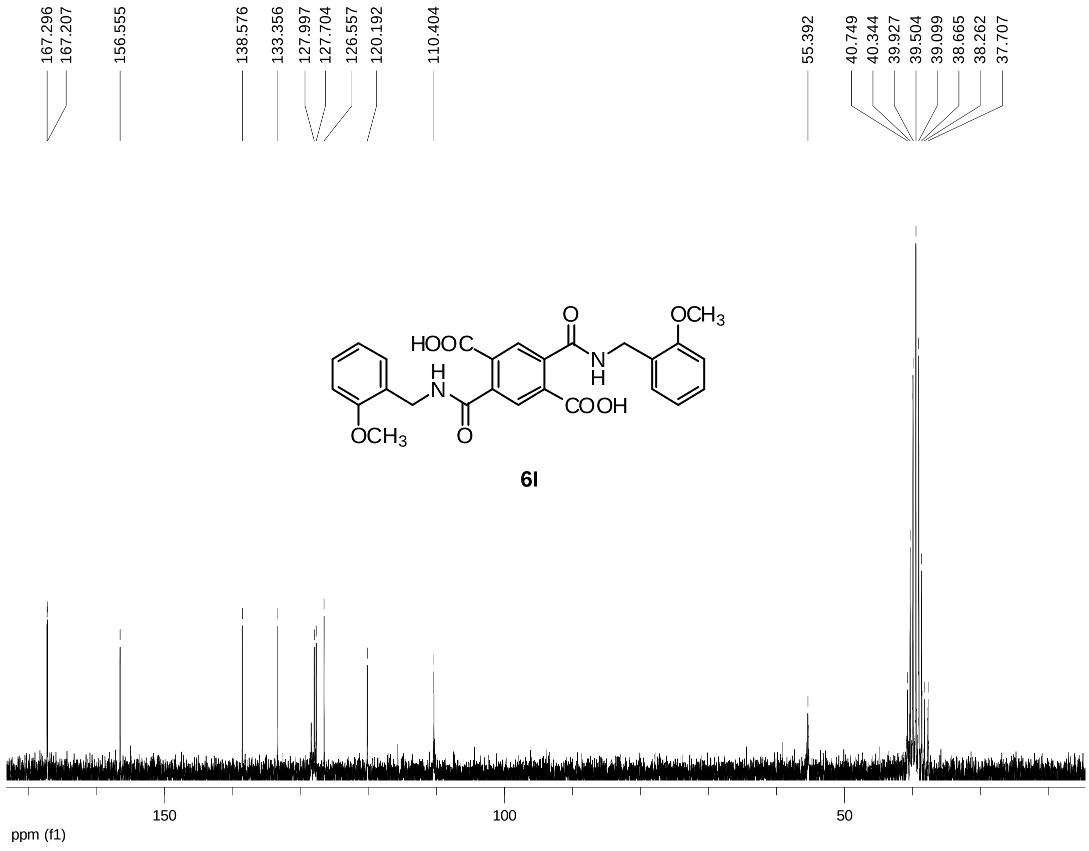
167.296
167.207
156.555
138.576
133.356
127.997
127.704
126.557
120.192
110.404
55.392
40.749
40.344
39.927
39.504
39.099
38.665
38.262
37.707
HOOC
COOH
OCH3
6l
150
100
50
ppm (f1)

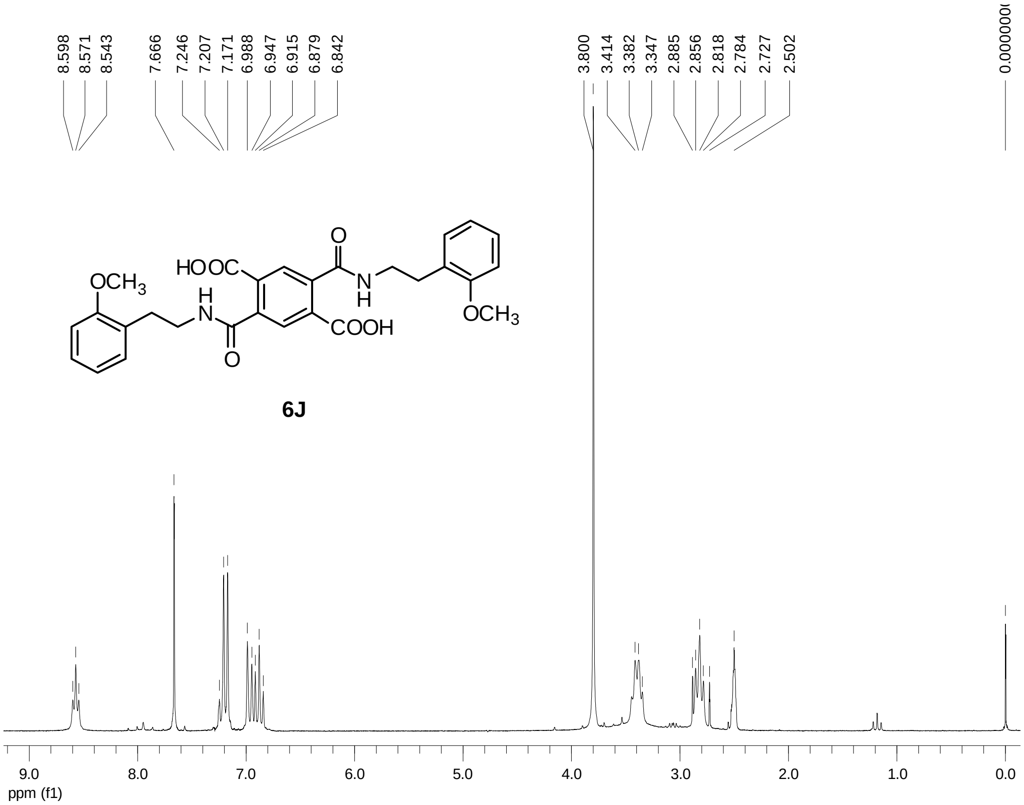
8.598
8.571
8.543
7.666
7.246
7.207
7.171
6.988
6.947
6.915
6.879
6.842
3.800
3.414
3.382
3.347
2.885
2.856
2.818
2.784
2.727
2.502
0.000000
HOOC
COOH
OCH3
N
H
O
6J
9.0
8.0
7.0
6.0
5.0
4.0
3.0
2.0
1.0
0.0
ppm (f1)

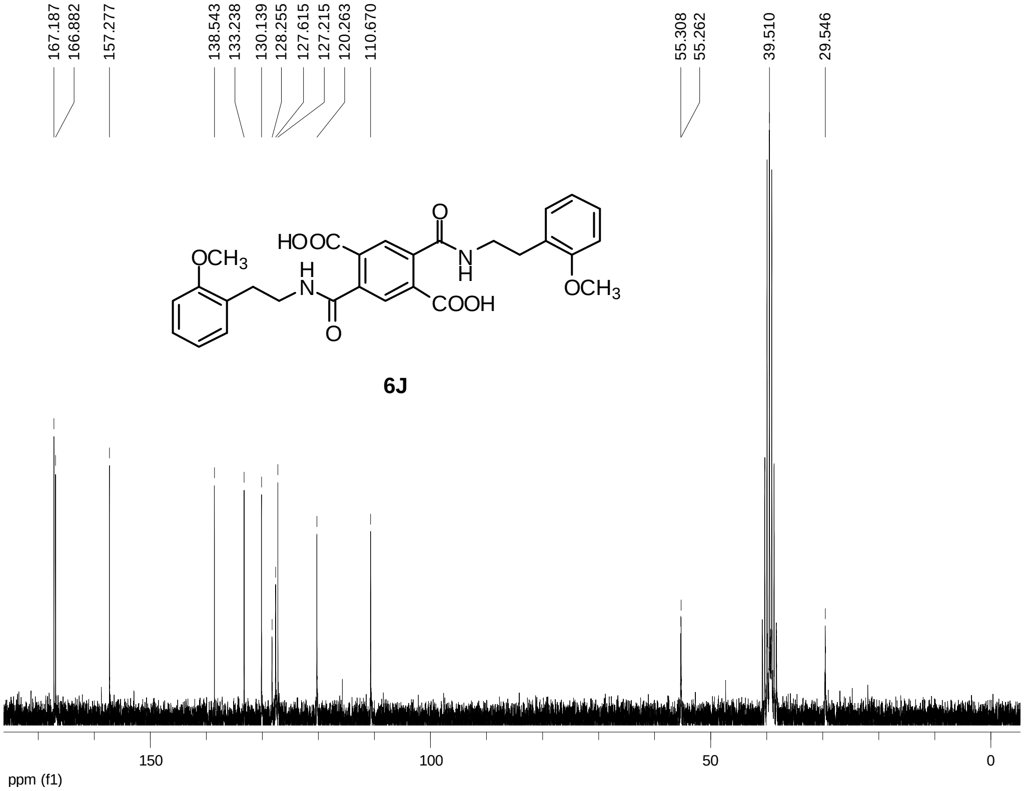
167.187
166.882
157.277
138.543
133.238
130.139
128.255
127.615
127.215
120.263
110.670
55.308
55.262
39.510
29.546
HOOC
COOH
OCH3
N
H
O
6J
150
100
50
0
ppm (f1)

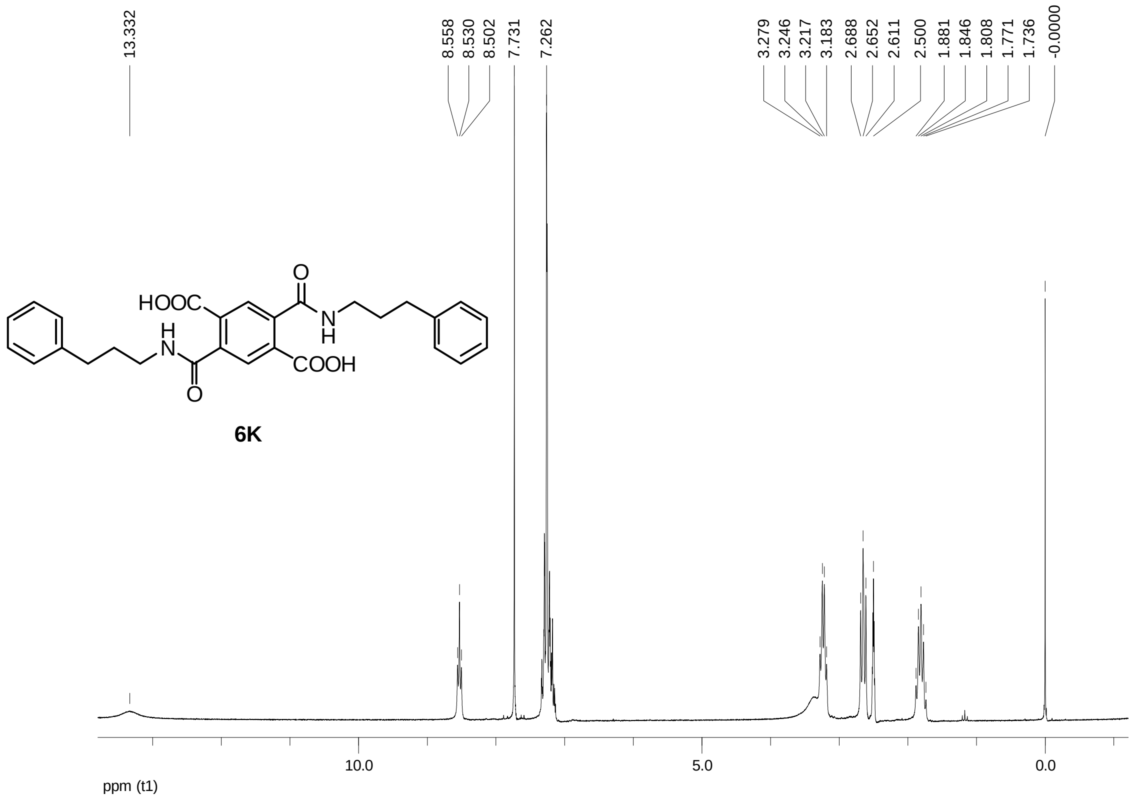
13.332
8.558
8.530
8.502
7.731
7.262
3.279
3.246
3.217
3.183
2.688
2.652
2.611
2.500
1.881
1.846
1.808
1.771
1.736
-0.0000
HOOC
COOH
6K
10.0
5.0
0.0
ppm (t1)

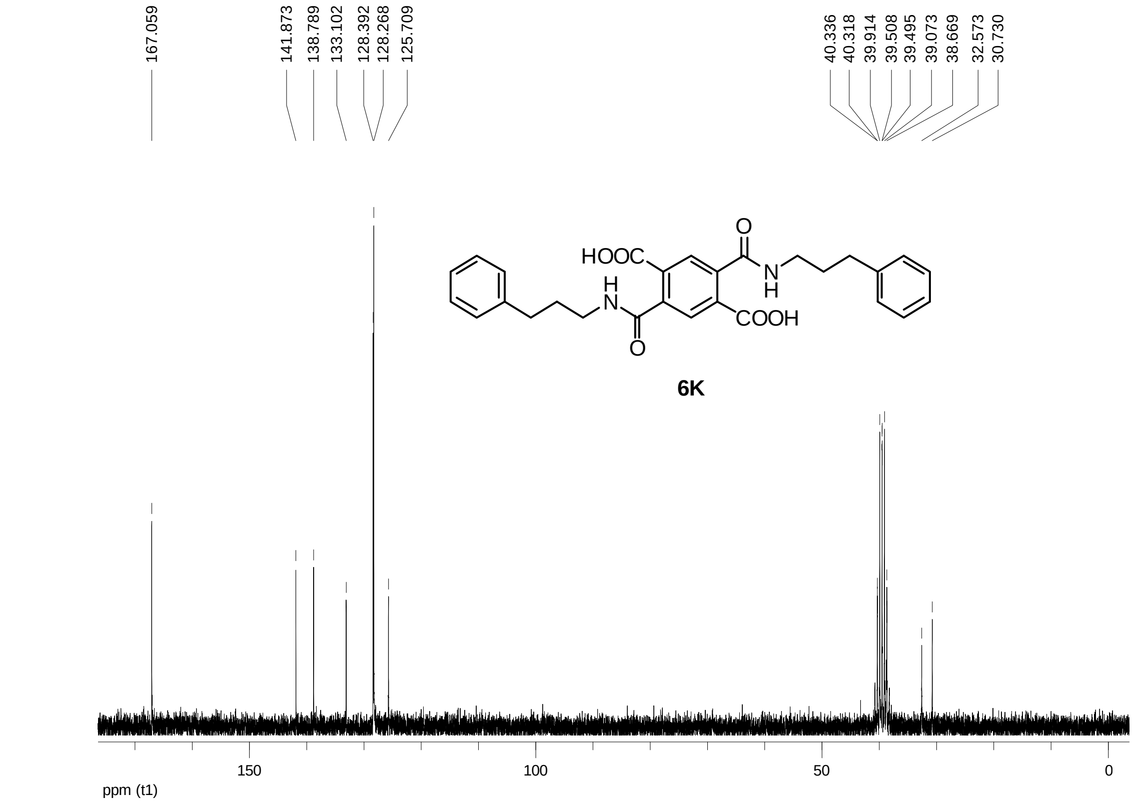
167.059
141.873
138.789
133.102
128.392
128.268
125.709
40.336
40.318
39.914
39.508
39.495
39.073
38.669
32.573
30.730
HOOC
COOH
6K
150
100
50
0
ppm (t1)

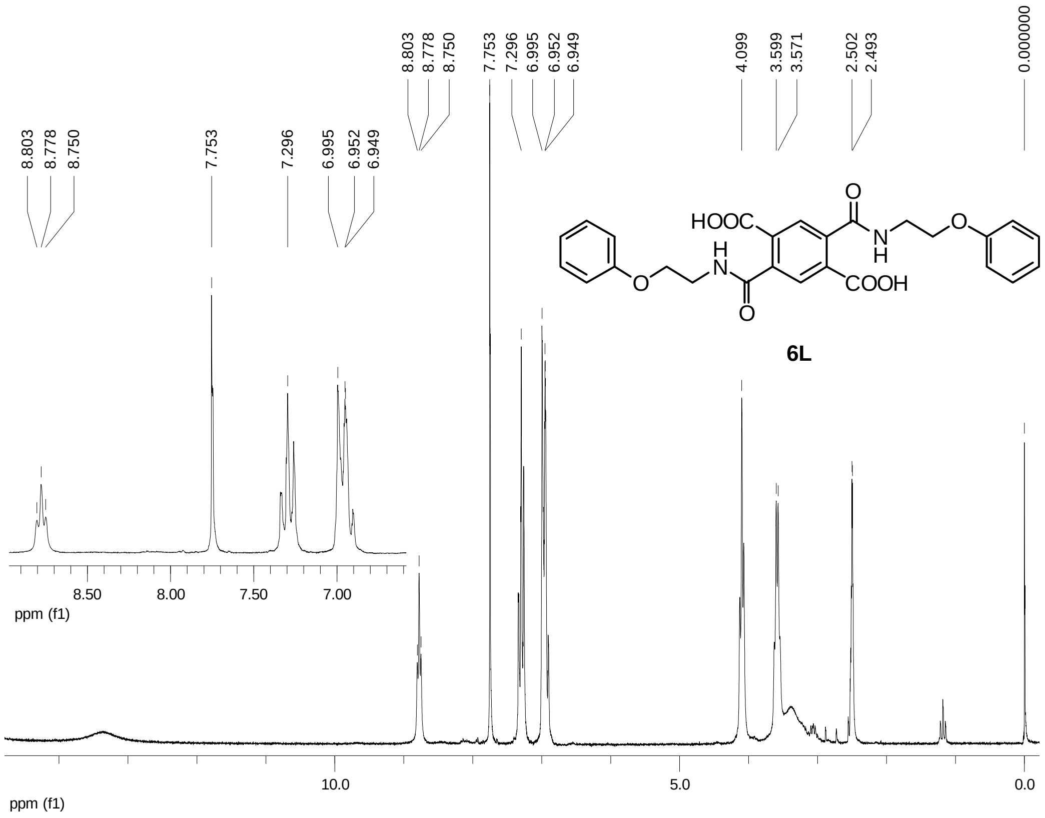
8.803
8.778
8.750
7.753
7.296
6.995
6.952
6.949
4.099
3.599
3.571
2.502
2.493
0.000000
HOOC
COOH
O
H
N
6L
8.50
8.00
7.50
7.00
ppm (f1)
10.0
5.0
0.0
ppm (f1)

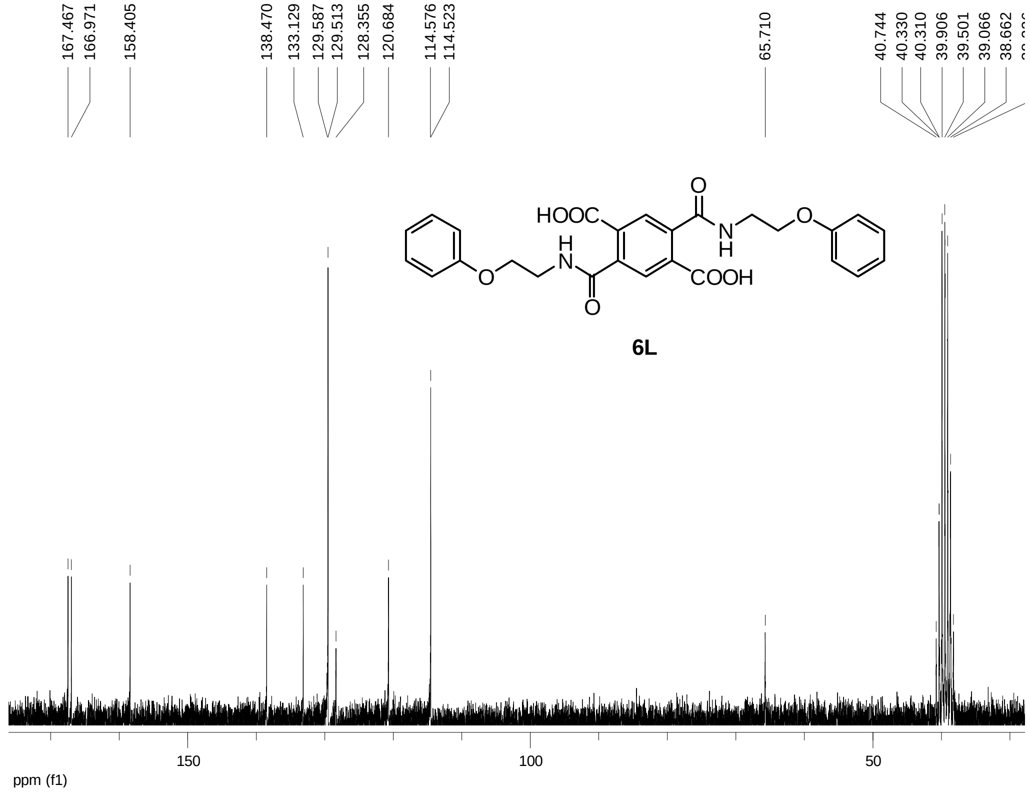
167.467
166.971
158.405
138.470
133.129
129.587
129.513
128.355
120.684
114.576
114.523
65.710
40.744
40.330
40.310
39.906
39.501
39.066
38.662
HOOC
COOH
O
H
N
6L
150
100
50
ppm (f1)

Reaction in Toluene

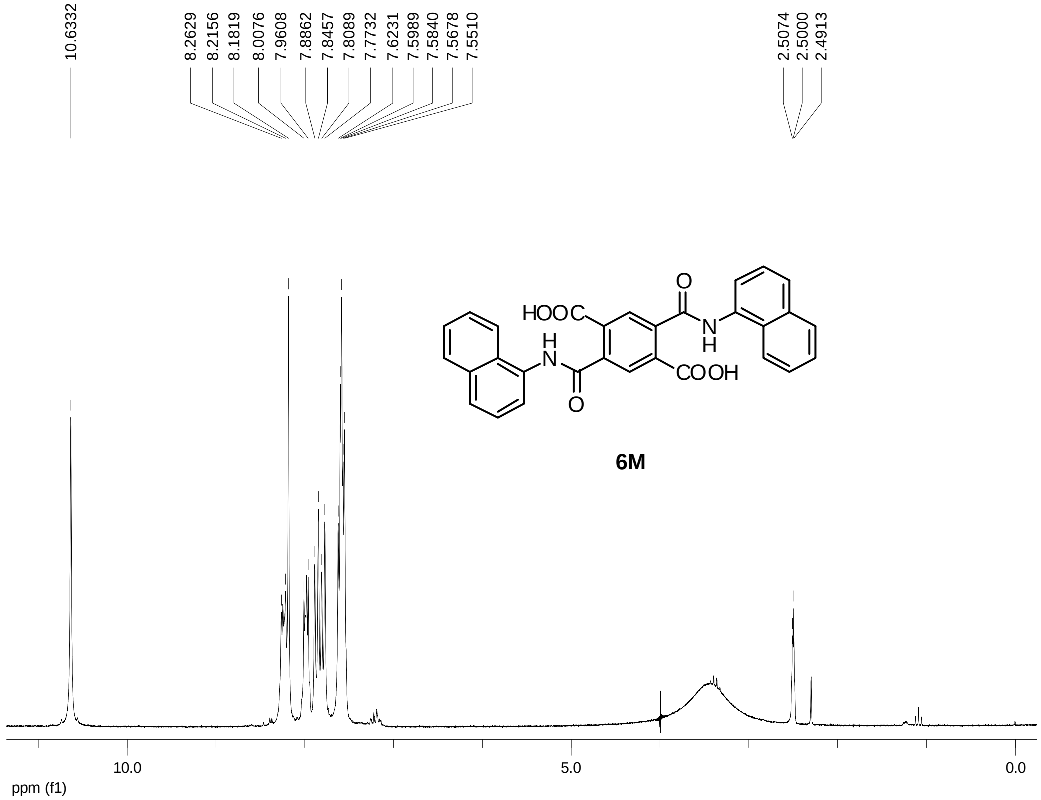

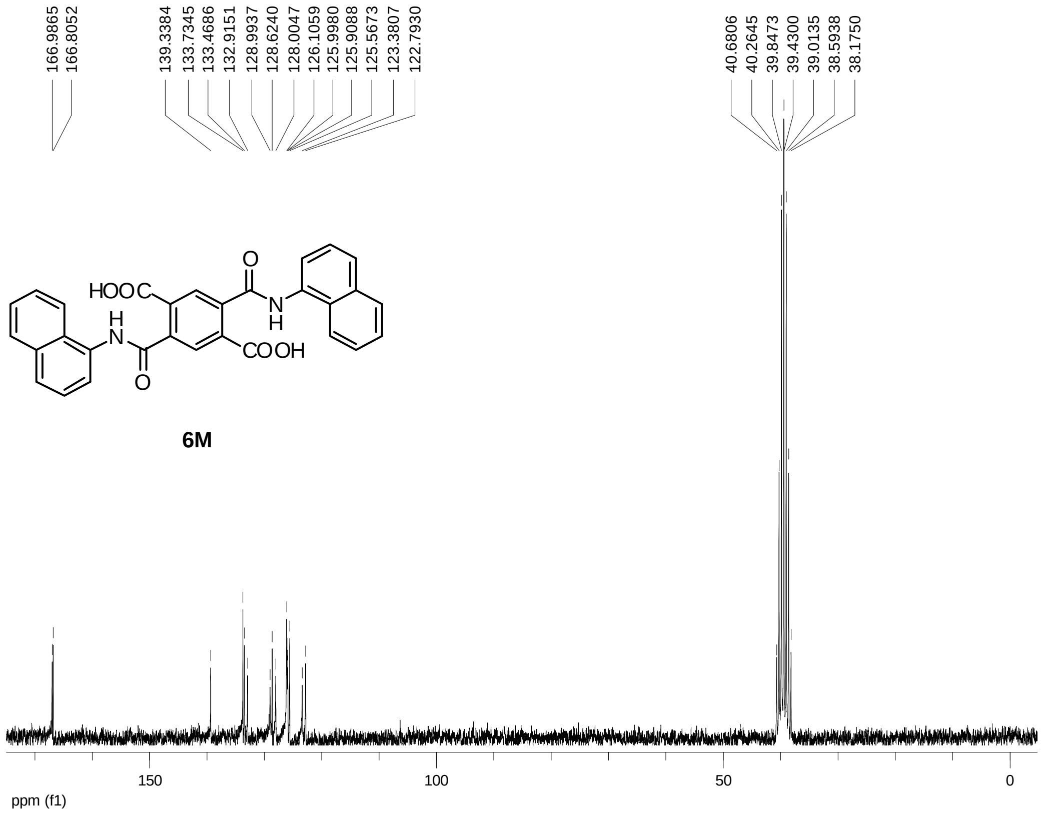

Reaction in Ethanol

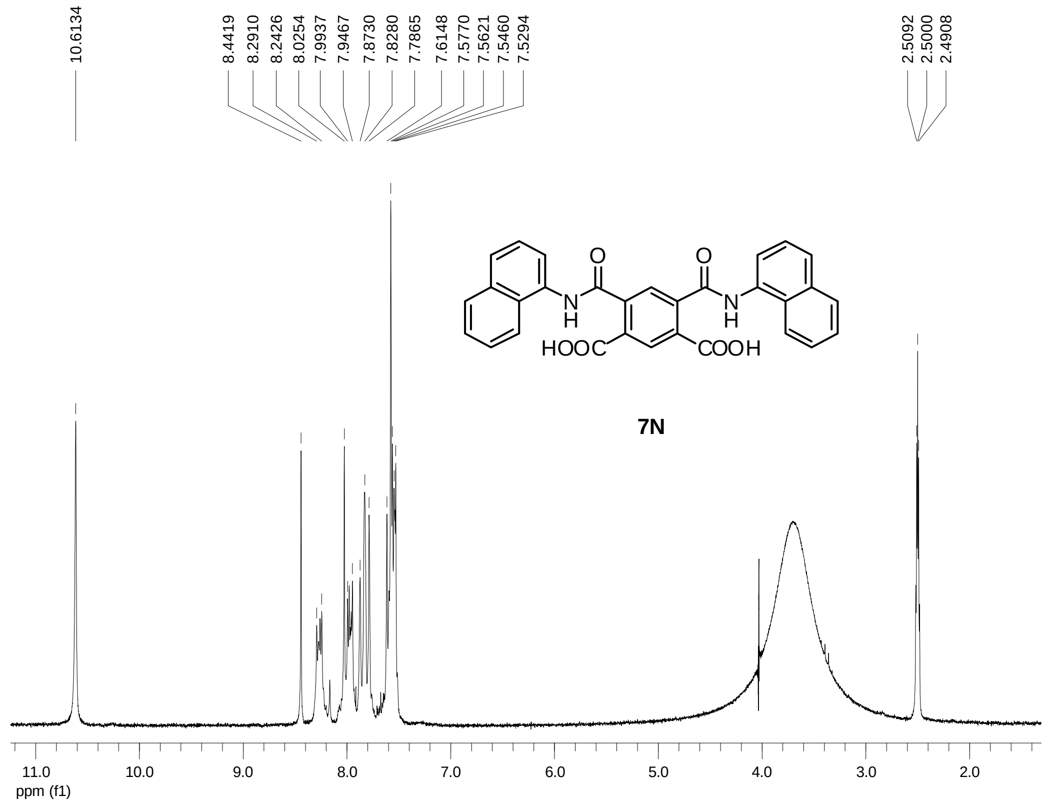

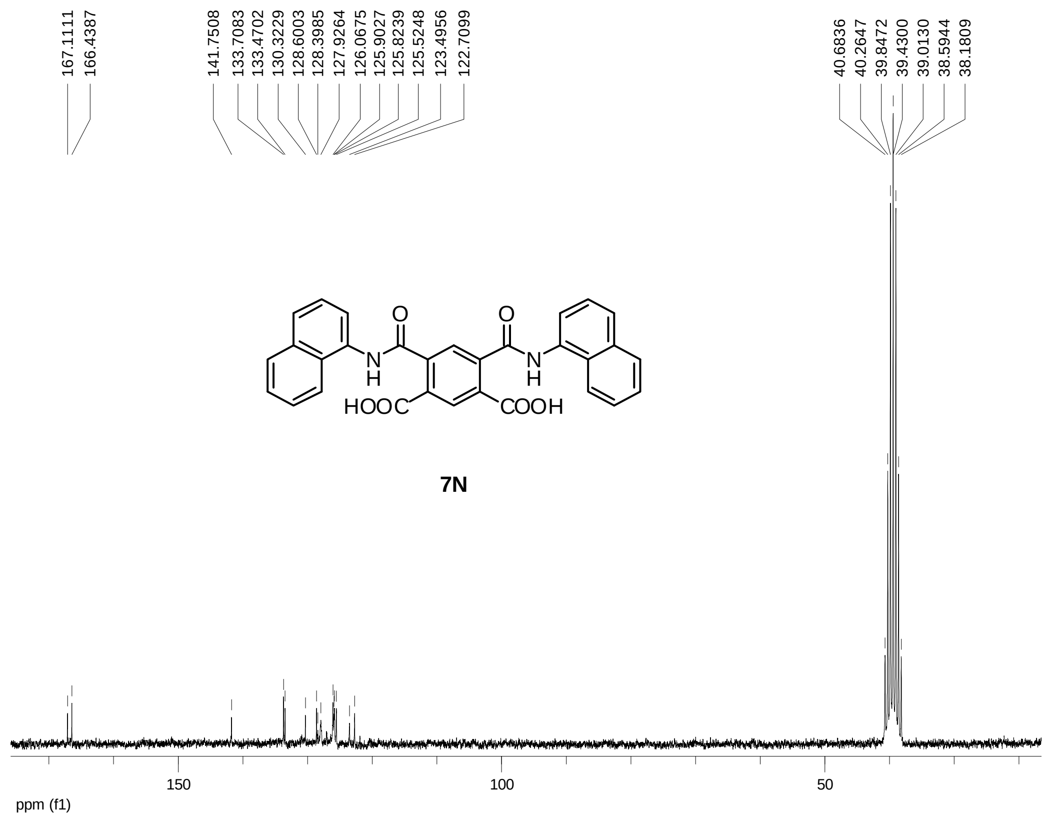

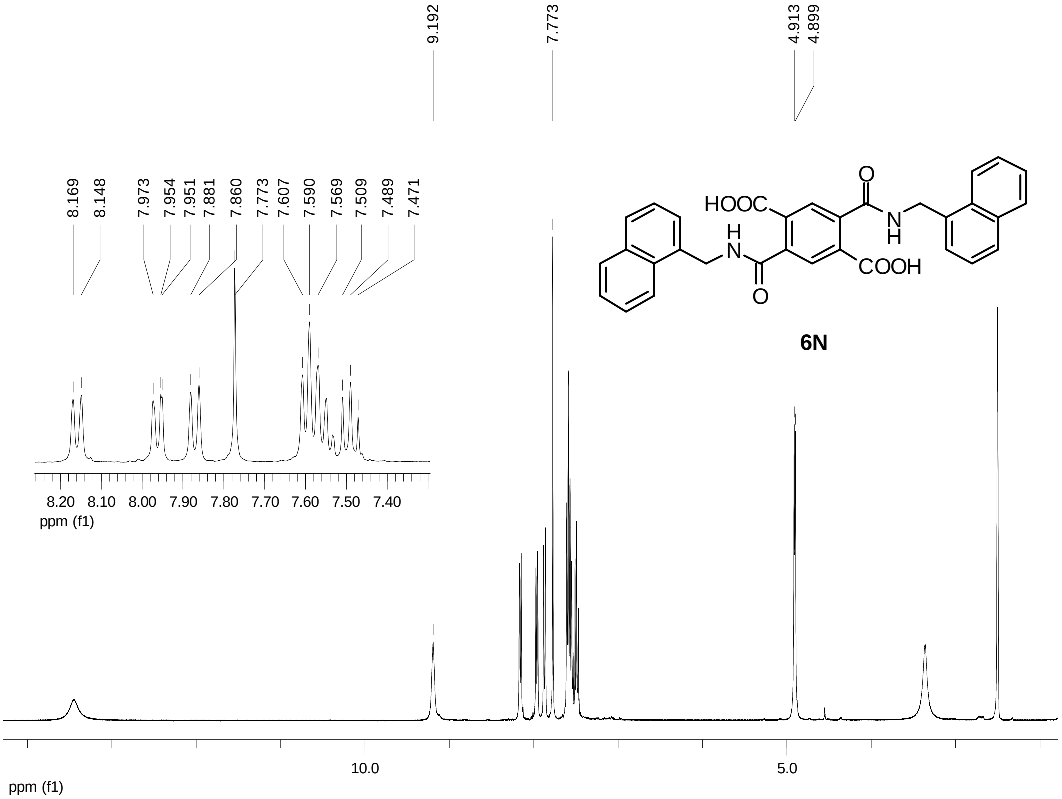
9.192
7.773
4.913
4.899
8.169
8.148
7.973
7.954
7.951
7.881
7.860
7.773
7.607
7.590
7.569
7.509
7.489
7.471
8.20 8.10 8.00 7.90 7.80 7.70 7.60 7.50 7.40
ppm (f1)
HOOC
COOH
O
H
N
6N
10.0
5.0
ppm (f1)

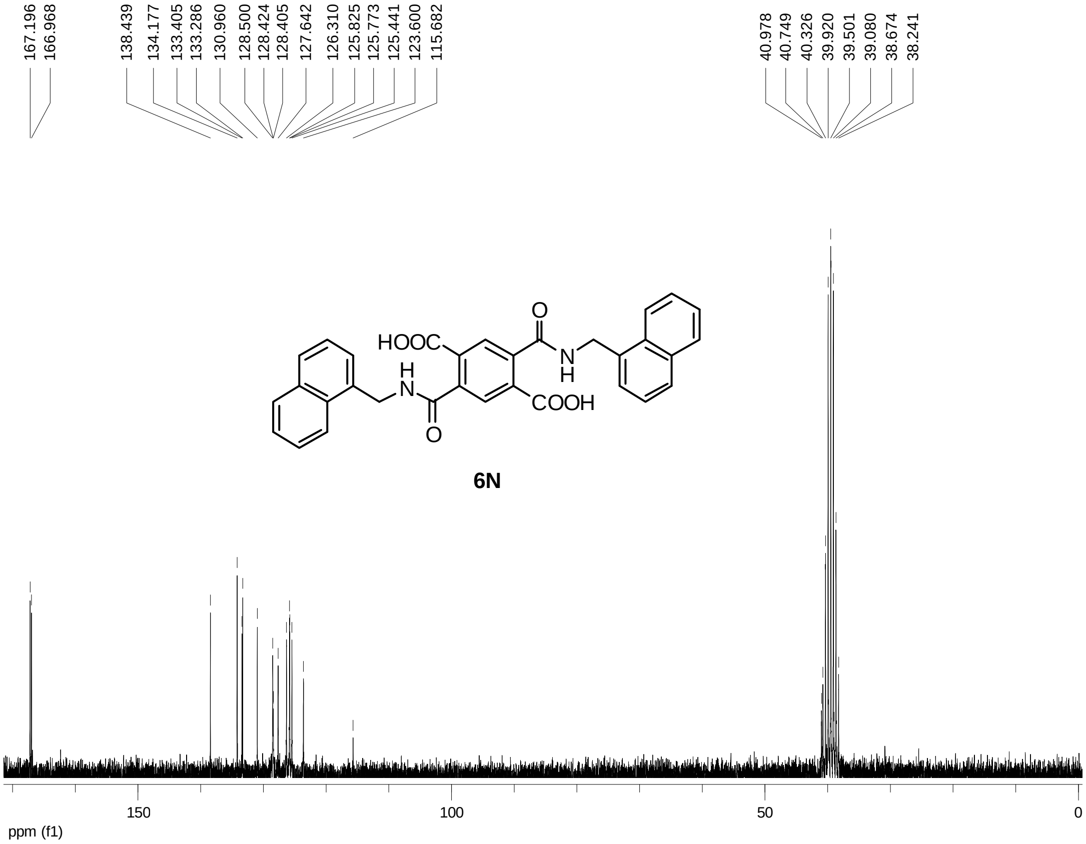
167.196
166.968
138.439
134.177
133.405
133.286
130.960
128.500
128.424
128.405
127.642
126.310
125.825
125.773
125.441
123.600
115.682
40.978
40.749
40.326
39.920
39.501
39.080
38.674
38.241
HOOC
COOH
O
H
N
6N
150
100
50
0
ppm (f1)
